# Opposing Wnt signals regulate cervical squamocolumnar homeostasis and emergence of metaplasia

**DOI:** 10.1038/s41556-020-00619-0

**Published:** 2021-01-18

**Authors:** Cindrilla Chumduri, Rajendra Kumar Gurumurthy, Hilmar Berger, Oliver Dietrich, Naveen Kumar, Stefanie Koster, Volker Brinkmann, Kirstin Hoffmann, Marina Drabkina, Panagiota Arampatzi, Dajung Son, Uwe Klemm, Hans-Joachim Mollenkopf, Hermann Herbst, Mandy Mangler, Jörg Vogel, Antoine-Emmanuel Saliba, Thomas F. Meyer

**Affiliations:** 1 Department of Molecular Biology, Max Planck Institute for Infection Biology, Berlin, Germany; 2 Chair of Microbiology, University of Würzburg, Würzburg, Germany; 3 Institute for RNA-based Infection Research (HIRI), Helmholtz Center for Infection Research (HZI), Würzburg, Germany; 4 Core Unit Systems Medicine, University of Würzburg, Würzburg, Germany; 5 Institute of Pathology, Vivantes Klinikum Berlin, Berlin, Germany; 6 Department of Gynecology, Charité University Medicine, Berlin, Germany; 7 Institute of Molecular Infection Biology, University of Würzburg, Würzburg, Germany; 8 Laboratory of Infection Oncology, Institute of Clinical Molecular Biology (IKMB), Christian Albrechts University of Kiel, Kiel, Germany; 9 Present Address: Klinik für Gynäkologie und Geburtsmedizin, Vivantes Auguste-Viktoria-Klinikum, Berlin, Germany

**Keywords:** Cancer, Developmental biology, Stem cells, Cell signalling

## Abstract

The transition zones of the squamous and columnar epithelia constitute hotspots for the emergence of cancer, often preceded by metaplasia, in which one epithelial type is replaced by another. It remains unclear how the epithelial spatial organization is maintained and how the transition zone niche is remodelled during metaplasia. Here we used single-cell RNA sequencing to characterize epithelial subpopulations and the underlying stromal compartment of endo- and ectocervix, encompassing the transition zone. Mouse lineage tracing, organoid culture and single-molecule RNA in situ hybridizations revealed that the two epithelia derive from separate cervix-resident lineage-specific stem cell populations regulated by opposing Wnt signals from the stroma. Using a mouse model of cervical metaplasia, we further show that the endocervical stroma undergoes remodelling and increases expression of the Wnt inhibitor Dickkopf-2 (DKK2), promoting the outgrowth of ectocervical stem cells. Our data indicate that homeostasis at the transition zone results from divergent stromal signals, driving the differential proliferation of resident epithelial lineages.

## Abstract (web summary)

Chumduri, Gurumurthy et al. show that cervical squamous and columnar epithelia derive from two stem cell populations, regulated by opposing Wnt signals, and that a Wnt-repressive environment can induce metaplasia.

## Main

Despite extensive self-organization abilities, the mucosal epithelial homeostasis is maintained by the local microenvironment, defined by complex interactions between epithelium and stroma. However, the microenvironment, when altered by various extrinsic and intrinsic factors, can result in disease. The boundaries between two different epithelial types (for example, between squamous stratified and columnar epithelia), termed transition zones, are found, for example, at endo–ectocervical and gastro–oesophageal junctions^1,2^. Transition zones are particularly susceptible to infections and development of neoplasia^1,2^. Cancers of transition zones occur as two major histological types: adenocarcinomas (ADC) and squamous cell carcinomas (SCC). Carcinogenesis at transition zones is often preceded by metaplasia—the replacement of one epithelium type by another^3^.

Cervical cancer is the fourth most common cancer in women^4^, more than 90% of which originate at the transition zone^5,6^. The cervix connects the uterine cavity with the vagina and consists of the ectocervix and the endocervix. The columnar epithelium lining the endocervix is contiguous with the uterine cavity and separated by the internal os, whereas the squamous epithelium of the ectocervix projects into the vagina. The external os marks the transition from the ectocervix to the endocervix, where the columnar epithelium meets the squamous epithelium, forming the squamocolumnar junction (SCJ). Under certain physiological or pathological conditions, the columnar epithelium at the SCJ is replaced by squamous metaplasia, creating a transformation zone^7–9^, but the underlying mechanisms remain unclear. Active metaplasia of the cervical transition zone is associated with risk of human papillomavirus (HPV) infection, an aetiological agent of cervical cancers^10–12^.

A diet deficient in vitamin A induces squamous metaplasia in mice^13^ and is associated with squamous metaplasia^14^ and a higher incidence of cervical cancer^15^ in humans. The earliest recognized sign of squamous metaplasia is the appearance of cuboidal cells beneath the columnar endocervical epithelium next to the transition zone. The origin of these cells has been variously assigned to undifferentiated subcolumnar reserve cells^16–18^, residual KRT7^+^ embryonic cells^19^, a basal cell ingrowth from the adjacent stratified epithelium, or even transdifferentiation of endocervical columnar or stromal cells^18^.

To identify the cellular subsets in the cervix and understand their interplay, we used single-cell RNA sequencing (scRNA-seq), to decipher regulatory relationships between individual cells in their niche context. Further, we used in vivo lineage tracing, tissue-mimetic epithelial 3D organoid models, single-molecule RNA in situ hybridization (smRNA-ISH) and a vitamin A-deficient mouse model of squamous metaplasia. We provide mechanistic insights into how cervical transition zone homeostasis is maintained, the cellular and molecular alterations that drive development of cervical squamous metaplasia and identify the cells that give rise to cervical ADC and SCC. Our study unravels the cervical cell subsets and reveals two committed adult epithelial stem cell types, which give rise to either squamous or columnar epithelial lineages. Homeostasis of these different epithelia at the SCJ is regulated by opposing Wnt gradients, and a shift towards a Wnt-repressive microenvironment drives squamous metaplasia.

## Results

### Distinct cellular origins of squamous and columnar epithelium

**Fig. 1:**
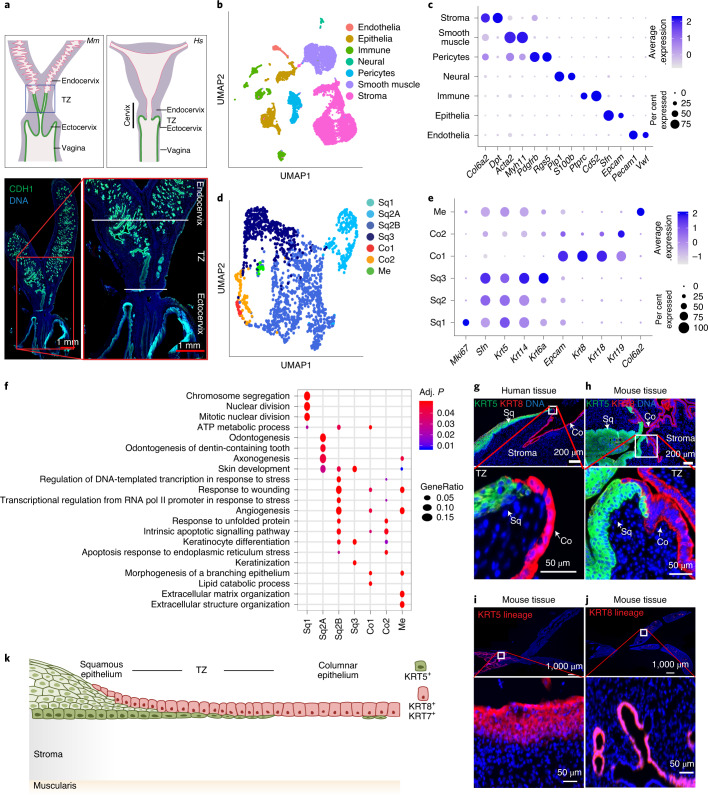
scRNA-seq of the cervix reveals distinct KRT5^+^ stratified and KRT8^+^ columnar epithelial lineages. **a**, Schematic of mouse (*Mm*) and human (*Hs*) female genital tract (FGT). Mouse FGT was outlined on the basis of immunostaining for the epithelial marker CDH1 (bottom) and markers in Fig. 8b and Extended Data Figs. 2b–d and 5. In humans and mice, the FGT consists of ovaries, fallopian tubes, uterus cervix and vagina. Mice (top left and bottom) have a bicornuate uterus (uterine horns), whereas humans (top right) have a single (simplex) uterus; both have a single vagina. Both species have ectocervix, endocervix and SCJ. The blue demarcation (top left), which includes the transition zone (TZ), serves to physically dissect this region from the endo- and ectocervical tissues. Boxed areas in the panel are enlarged; endocervix, transition zone and ectocervix are labelled; nuclei are labelled in blue. Data representative of *n* = 3 independent biological samples. **b**–**e**, scRNA-seq of healthy mouse ectocervix, endocervix and transition zone tissues. *n* = 3 biologically independent mouse experiments. **b**, Uniform manifold approximation and projection (UMAP) of the cellular subclusters. Single cells are colour coded by cluster annotation. **c**, Dot plot showing the expression of established markers associated with each cluster. Circle size indicates the percentage of cells in which the gene expression was detected. Fill colour depicts the normalized and scaled mean expression level. **d**, UMAP of epithelial subclusters colour coded by the cluster annotation (Co, columnar; Sq, squamous; Me, myoepithelial). **e**, Dot plot showing expression of markers within epithelial subclusters. Circle size and coloured scale as in **c**. **f**, Selection of gene ontology (GO) enrichment terms associated with each epithelial subcluster (Supplementary Table 3). Adj. *P*, adjusted *P*-value. **g**,**h**, Top: transition zone, including stratified and columnar epithelium from human (**g**) and mouse (**h**) cervix tissue sections immunolabelled with antibodies against KRT5 and KRT8; nuclei in blue. **i**,**j**, Top: tiled images of tissue sections from the genital system of 16-week-old *Krt5-Cre*^*ERT2*^*;Rosa26-tdTomato* (**i**) and *Krt8-Cre*^*ERT2*^*;Rosa26-tdTomato* (**j**) mice after tamoxifen induction at 4 weeks of age. **g**–**j**, Bottom: magnified view of boxed areas. **k**, Schematic representation of the stratified and columnar lineages and the transition zone of the cervix. Tiled images were acquired with an AxioScan imager. Data in **g**–**j** are representative of *n* = 3 biologically independent mice or human samples.

**Extended Data Fig. 1:**
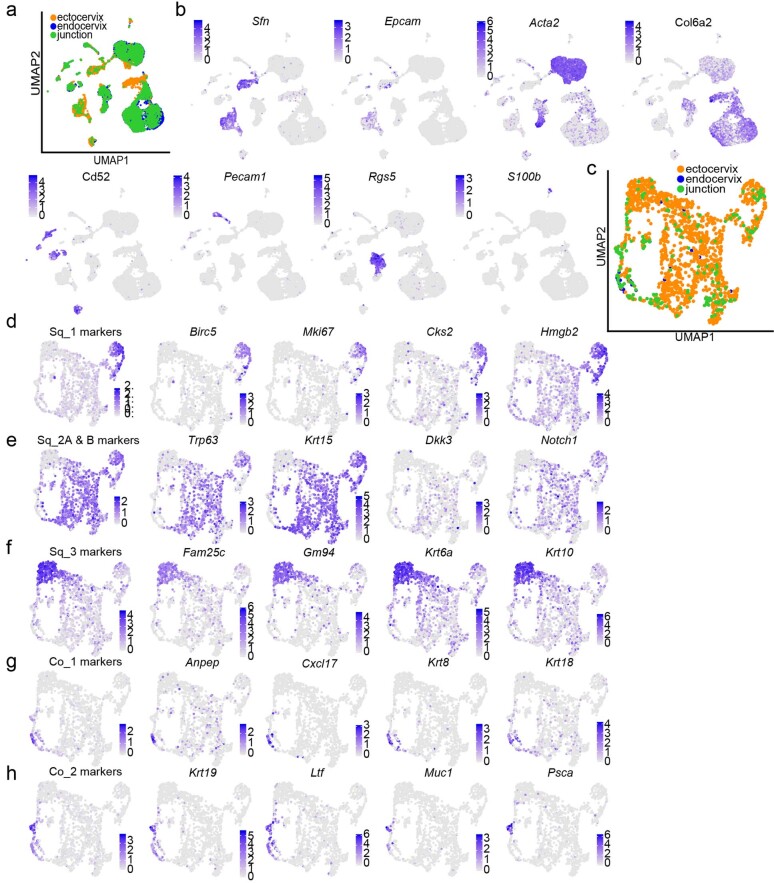
scRNA-seq profiling of the cervix. **a**, UMAP of single cell transcriptomes obtained from healthy mouse endocervix, ectocervix and TZ as in Fig. 1b. Here, cells are labelled by the tissue of origin. **b**, Normalized expression of selected markers (*Sfn, Epcam, Acta2, Col6a2, Cd52, Pcam1, Rgs5* and *S100b*) colour-coded on the UMAP, representing cellular sub-clusters. **c**, UMAP of epithelial sub-clusters colour-coded by the tissue of origin. **d-h**, Normalized expression values of selected markers colour coded on the UMAP representing key epithelial sub-clusters as in Fig. 1d. Color maps of squamous type (Sq_1): *Birc5, Mki67, Cks2, Hmgb2*; Sq_2A and Sq_2B: *Trp63, Krt5, Dkk3*, *Notch1*; Sq_3: *Fam25c, Gm94, Krt6*, *Krt10*; Columnar type (Co_1): *Anpep, Cxcl17, Krt8, Krt18*; Co_2: *Krt19, Ltf, Muc1, Psca*; n = 3 biologically independent mice experiments.

To determine the basic cellular features of the mouse endocervix, the ectocervix and the transition zone, we performed scRNA-seq of isolated cells from each tissue region (Fig. 1a, left). The generated data were combined to perform unsupervised clustering, and cluster identity was assigned on the basis of cell-type-specific marker expression (Fig. 1b,c, Extended Data Fig. 1a,b and Supplementary Table 1). Whereas the pattern of cell types in the endocervix was similar to that of the transition zone, subpopulations of epithelial, stromal, immune and smooth muscle cells in the ectocervix showed distinct transcriptional profiles (Fig. 1b and Extended Data Fig. 1a). An independent cluster analysis on the epithelial cell population to characterize their heterogeneity revealed two major epithelial cell types: *Krt5*^hi^/*Krt14*^hi^ squamous (Sq) and *Krt8*^hi^/*Krt*19^hi^ columnar (Co) cells. These were further divided into six transcriptionally distinct subclusters (Sq1, Sq2A, Sq2B, Sq3, Co1 and Co2) (Fig. 1d,e, Extended Data Fig. 1c and Supplementary Table 2). We designated myoepithelial cells (Me) as a *Krt5*^hi^/*Krt*14^hi^ subcluster that also expressed fibroblast markers, including *Col6a2* (Fig. 1d,e). The Sq1 subcluster was enriched for mitotic genes and expressed *Trp63, Birc5, Mki67, Cks2* and *Hmgb2*, indicating basal stem cells of the stratified ectocervix (Fig. 1f and Extended Data Fig. 1d–f). The Sq2A and Sq2B subclusters were enriched for skin development and stress or wounding response genes and expressed *Trp63, Krt15, Dkk3* and *Notch1*. The Sq3 subcluster was enriched for keratinocyte differentiation and skin development genes and expressed *Fam25c, Gm94, Krt6a* and *Krt10*, indicating differentiated squamous cells. The Co1 subcluster was enriched for genes for morphogenesis of branching epithelium and lipid catabolic processes, and expressed *Anpep, Cxcl17, Krt8* and *Krt18*. The Co2 subcluster was enriched for genes for stress response, apoptosis and response to unfolded proteins, and expressed *Krt19, Ltf, Muc1* and *Psca* (Fig. 1f, Extended Data Fig. 1g,h and Supplementary Table 3).

**Extended Data Fig. 2:**
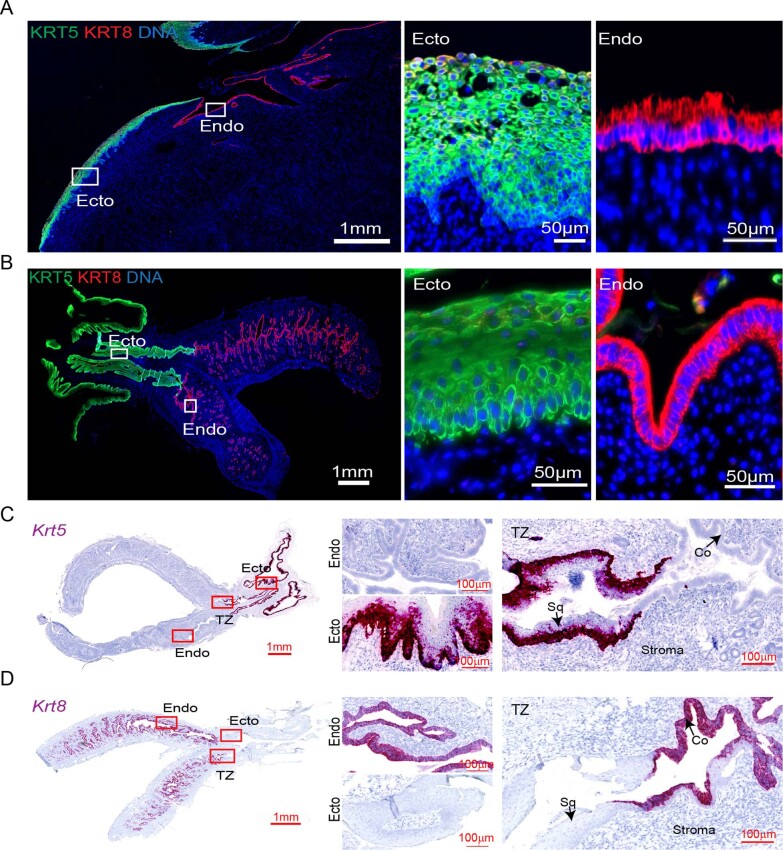
Cervix consists of KRT5^+^ stratified and KRT8^+^ columnar epithelium. (**a-b**) Human (**a**) and mouse (**b**) tiled images of cervix tissue sections, including stratified and columnar epithelium, immunolabeled against KRT5 and KRT8; nuclei in blue. **c-d**, Tiled brightfield images of sections showing the entire mouse female genital system labelled with smRNA-ISH for *Krt5* (**c**) and *Krt8* (**d**); nuclei in blue. Boxed areas are magnified on the right. Images are representative of n = 3 biologically independent mice or human samples.

Fluorescence immunohistochemistry and smRNA-ISH confirmed that KRT5 and KRT8 mark the entire squamous stratified and columnar epithelia, respectively (Fig. 1g,h and Extended Data Fig. 2a–d). To test whether these two epithelia originate from distinct cell types, we used *Krt5-Cre*^*ERT2*^*;Rosa26-tdTomato* and *Krt8-Cre*^*ERT2*^*;Rosa26-tdTomato* mice to perform lineage tracing. Twelve weeks after induction, KRT5^+^ cells exclusively labelled the stratified epithelium, whereas KRT8^+^ cells exclusively labelled the endocervical epithelium (Fig. 1i,j). Both epithelia merged at the transition zone, with KRT5^+^ cells appearing to displace overlying KRT8^+^ columnar cells (Fig. 1k and Extended Data Fig. 2a,b). Thus, these two major epithelial cell types of the postnatal cervix originate from two distinct lineages.

### Opposing stromal Wnt signals define the epithelial borders at the transition zone

**Fig. 2:**
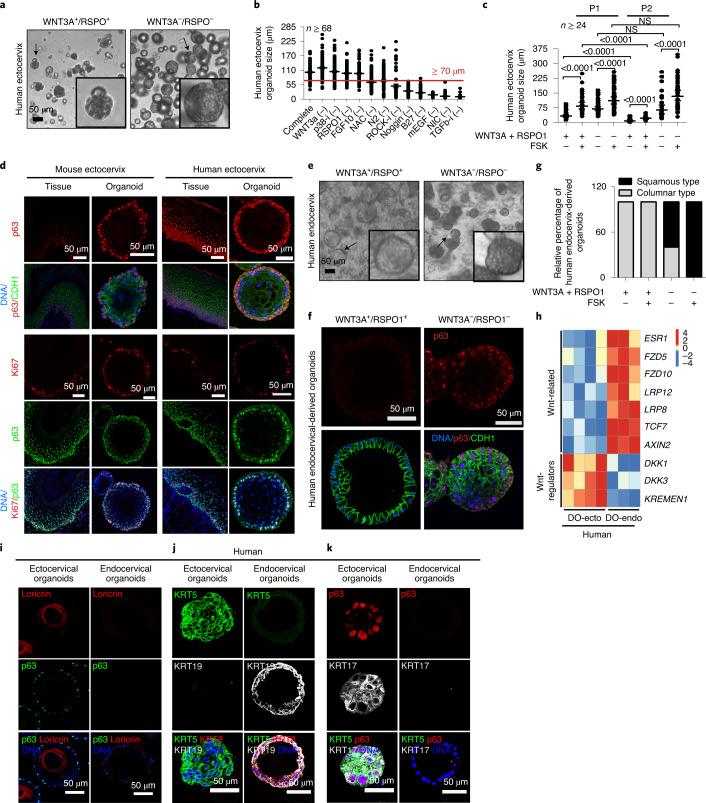
Wnt-signalling pathway agonists and antagonists have a crucial role in ecto- and endocervical development. **a**, Bright-field images of human ectocervical organoids. Cells isolated from ectocervical tissue were grown in Matrigel with different factors. Efficient squamous stratified organoid formation was dependent on the absence of WNT3A and RSPO1. Magnified images of organoids indicated with an arrow are included as insets. **b**, Mouse ectocervical organoid formation quantified by area in the absence of different components in the growth medium. Red line, 70 µm diameter; *n* represents the number of organoids quantified. p38-i, SB202190 (p38 inhibitor); NAC, *N*-acetyl-l-cysteine (inhibits reactive oxygen species formation); ROCK-i, Y-27632 (ROCK inhibitor); mEGF, mouse EGF; NIC, nicotinamide; TGFb-i, TGF-β receptor kinase inhibitor IV. **c**, Effect of growth factors on the ectocervical organoid size from passage (P)1 to P2. Data are mean ± s.e.m and *P*-values are shown on the graph. Statistical significance was determined using a two-tailed Student’s *t*-test. *n* represents the number of organoids quantified. **d**, Confocal images of sections from human and mouse cervix and organoids immunolabelled for CDH1, Ki67 and p63; nuclei are labelled in blue. Images are representative of *n* = 3 biologically independent mice or human samples. **e**, Bright-field images of human endocervical organoids. Cells isolated from endocervical tissue were grown in Matrigel with different factors. Wnt signalling was essential for the formation of columnar organoids, whereas its absence drove the formation of stratified squamous organoids. Magnified images of organoids indicated with an arrow are included as insets. **f**, Columnar and stratified human organoids derived from the endocervix, containing p63^**−**^ (columnar) and p63^**+**^ (stratified) cells. Both cell types expressed the epithelial marker CDH1. Images in **a**,**e**,**f** are representative of organoid cultures from biologically independent experiments derived from five donors. **g**, Percentage of columnar and squamous stratified organoids formed from human endocervical cells in the presence of different growth factors. *n* ≥ 30 organoids. **h**, Expression analysis of differentially regulated genes in human ectocervical versus endocervical organoids. Wnt-related genes were expressed at higher levels in endocervical organoids, whereas Wnt-regulator genes were expressed at higher levels in ectocervical organoids. Columns represent individual donors. Colour bar represents z-scored gene expression. **i**–**k**, Confocal images of human ectocervical stratified and endocervical columnar organoids immunolabelled for p63 and loricrin (**i**), KRT5, KRT8 and KRT19 (**j**), and p63, KRT5 and KRT17 (**k**). Data representative of independent biological samples from three donors. Statistical source data are provided in Source Data Fig. 2. Source data

**Extended Data Fig. 3:**
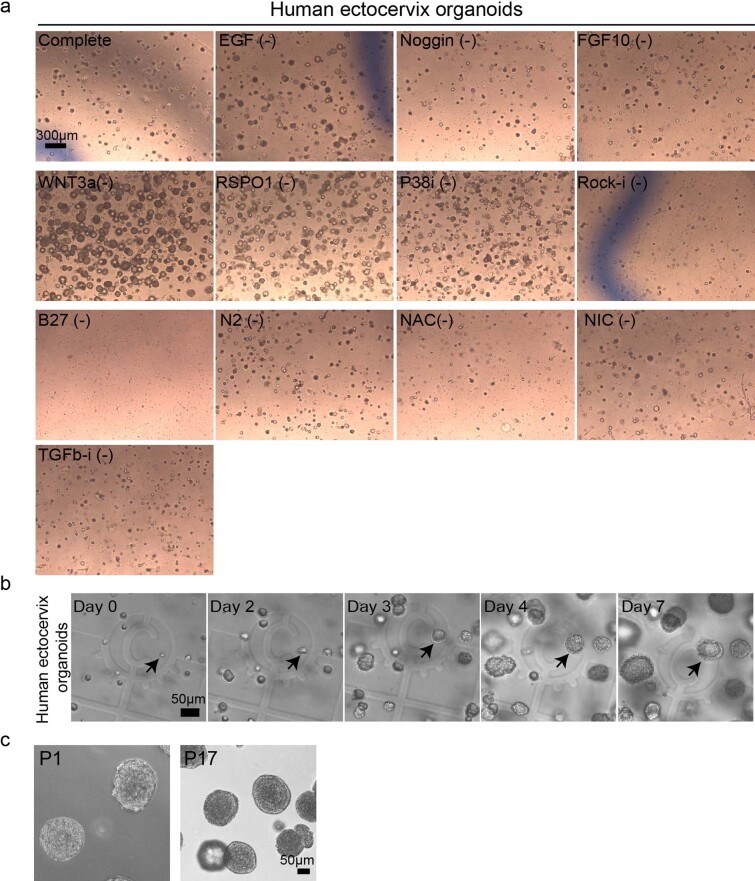
Culture conditions for human and mouse ectocervical organoids derived from single epithelial stem cells. **a-c**, Bright-field images showing (**a**) two-week-old human ectocervical organoids grown in the absence of indicated growth factors from the complete media; (**b**) Time course of organoids grown from single ectocervical stem cells; (**c**) Maintenance of stemness from the passage (P)1 to P17 in mouse ectocervical organoids. Data representative of n = 3 biologically independent mice or human samples.

**Extended Data Fig. 4:**
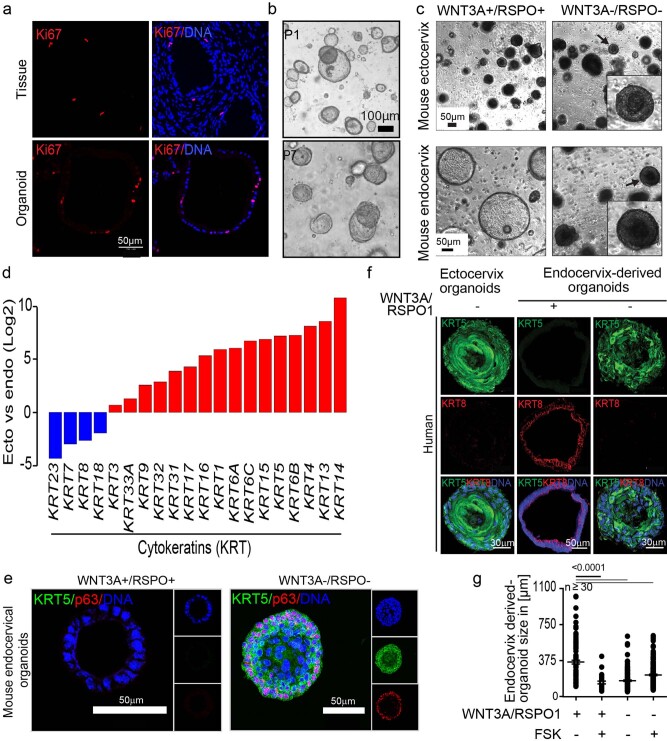
Wnt microenvironment controls the growth of endocervical organoids. **a**, Confocal images show a similar distribution of Ki67 in human endocervical tissue and organoids. **b**, Brightfield images of human endocervical organoids at P1 and P7. (**a-b**) Data representative of n = biologically independent experiments from 3 donors. **c**, Brightfield images of mouse ecto- and endocervical organoids. Cells isolated from ecto- and endocervical tissue were grown in Matrigel with WNT-proficient or -deficient medium. Data representative of n = biologically independent experiments from 3 mice. **d**, Analysis of differential expression of cytokeratins in human ecto- vs endocervical organoids, revealing a distinct expression profile. **e**, Confocal images of endocervix tissue-derived organoids immunolabeled for KRT5 and p63; nuclei in blue. Data representative of n = biologically independent experiments from 3 mice. **f**, Confocal images of organoids-derived from human ectocervix or endocervix that were grown in the presence or absence of WNT3A and RSPO1 and immunolabeled for KRT5 and KRT8; nuclei in blue. Data representative of n = biologically independent experiments from 3 donors. **g**, Effect of growth factors on endocervical organoid size. Data represented as mean ± s.e.m. n = shown are the number of organoids quantified from a representative of 3 independent biological replicates. Statistical significance was determined using a two-tailed Student’s t-test, and p-values are shown on the graph. Statistical source data are provided in Source Data Extended Data Fig. 4. Source data

To identify which niche-derived signalling maintains these two lineages, we established stem cell-derived organoid models under defined conditions that facilitate long-term propagation. We tested various factors known to play a role in the maintenance of diverse adult stem cells, including the canonical Wnt agonists WNT3A and R-spondin-1 (RSPO1), FGF10, EGF, hydrocortisone, the cAMP pathway agonist forskolin (FSK), the BMP signalling inhibitor noggin, nicotinamide and the TGF-β pathway inhibitor A83-01^20–25^. EGF, FGF10, A83-01 and active BMP signalling were essential for the long-term maintenance of squamous stratified organoids derived from human and mouse ectocervix. By contrast, the presence of WNT3A and RSPO1 was detrimental for both the formation and long-term expansion of ectocervical organoids (Figs. 2a,b and Extended Data Fig. 3a,b). Growth was further increased in the presence of FSK (Fig. 2c). Because cAMP signalling is essential for EGF-mediated neuronal stem cell proliferation^26^, we speculate that FSK also synergizes EGF signalling in ectocervical stem cells. Ectocervical organoids from both humans and mice could be maintained for more than six months (Extended Data Figs. 3c and 6a). They fully recapitulated the in vivo tissue architecture with stratified layers decorated with E-cadherin (CDH1) (Fig. 2d). The outer layer consisted of p63^+^ basal cells that expressed the proliferation marker Ki67; differentiation into parabasal cells was consistent with p63 labelling decreasing towards the lumen (Fig. 2d). Cells derived from human endocervix gave rise to hollow organoids of a simple columnar epithelial layer when cultured in the presence of Wnt-proficient medium containing WNT3A and RSPO1 (Fig. 2e,f). These organoids faithfully resembled the in vivo tissue architecture with sporadic Ki67 staining (Extended Data Fig. 4a). Endocervical organoids could be maintained for more than seven months (Extended Data Fig. 4b).

Of note, endocervix-derived cells cultured in a Wnt-deficient medium (that is, without WNT3A and RSPO1) gave rise to p63^+^, KRT5^+^ stratified organoids, resembling ectocervix organoids (Fig. 2e–g and Extended Data Fig. 4c,e–g). Conversely, ectocervix-derived cells gave rise to only squamous organoids independently of Wnt (Fig. 2a). Since formation of columnar organoids was Wnt-dependent, unlike that of squamous organoids, we investigated the source of Wnt signals in the cervix. Microarray analysis of organoids shows that the transcriptional regulation of Wnt in endocervical cells diverged from that in the ectocervix; Wnt agonists were upregulated in the columnar epithelium, whereas the Wnt antagonists *DKK1* and *KREMEN1* were upregulated in the squamous epithelium (Fig. 2h and Supplementary Table 5). Further, transcriptional profiling and confocal microscopy of ecto- and endocervix-derived human organoids revealed distinct patterns of keratin expression in congruence with data from the respective tissues (Fig. 1e and Extended Data Figs. 1e–h and 4d). Ectocervical organoids expressed KRT5, p63, KRT17 and the luminal cell marker loricrin, whereas endocervical columnar organoids expressed KRT8 and KRT19 (Fig. 2i–k and Extended Data Fig. 4f).

**Fig. 3:**
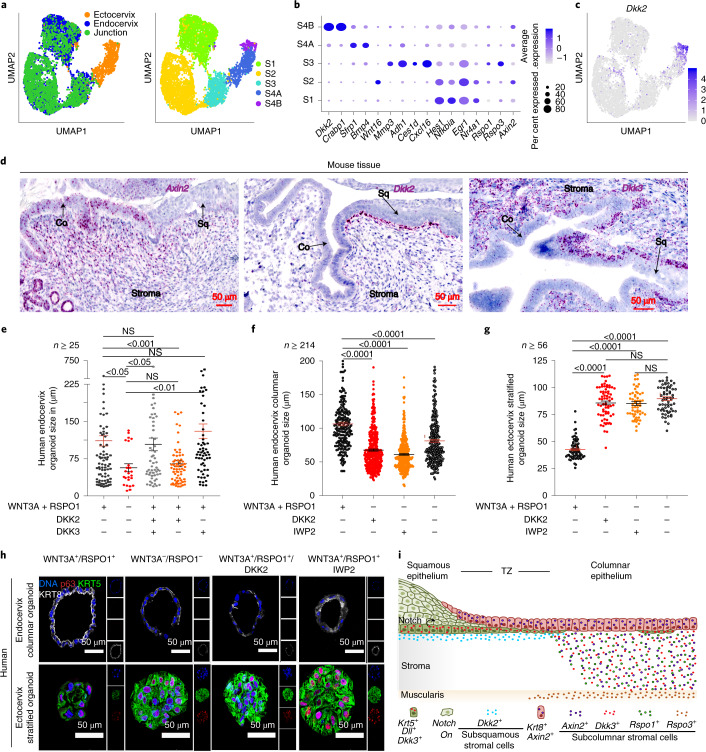
Stromal compartments of ecto- and endocervix show distinct patterns of expression. **a**, UMAP plot of stromal subclusters from healthy mouse ectocervix, endocervix and transition zone tissues coloured by sampled tissue (left) or by cluster annotation (right). **b**, Dot plot showing the expression of marker genes within stromal subclusters. Circle size indicates the percentage of cells in which the gene expression was detected. Fill colour depicts the normalized and scaled mean expression level. **c**, Normalized expression values of *Dkk2* on the UMAP. Colour bar represents normalized gene expression. **d**, smRNA-ISH of mouse transition zone for *Axin2*, *Dkk2* and *Dkk3*; nuclei in blue. Tiled images were acquired with an AxioScan imager and are representative of *n* = 3 biologically independent mice samples. **e**–**h**, Columnar organoids derived from human endocervix (**e**, **f** and **h**, top) and stratified organoids derived from human ectocervix (**g** and **h**, bottom). They were reseeded as single cells in Matrigel and allowed to form organoids in the presence of the indicated factors. *n* is the number of organoids quantified from a representative of three biologically independent experiments. **e**–**g**, Organoid size. Data are mean ± s.e.m. Statistical significance was determined using a two-tailed Student’s *t*-test, *P*-values are shown on the graph. NS, not significant. **h**, Confocal images of organoid sections immunolabelled for KRT5, KRT8 and p63; nuclei in blue. Representative of biologically independent experiments from three donors. **i**, Schematic representation of the distinct epithelial lineages and the underlying tissue microenvironment at the transition zone. Statistical source data are provided in Source Data Fig. 3. Source data

To gain insights into how stromal factors contribute to these two distinct cervical epithelial homeostases, we determined the heterogeneity of stromal populations in the endocervix, ectocervix and transition zone. Unsupervised clustering analysis of combined scRNA-seq data revealed five transcriptionally distinct subclusters of stromal populations (Fig. 3a and Supplementary Table 4). The stromal 1 (S1), S2, and S3 subclusters represented cells from endocervix and transition zone, commonly expressing *Hes1*, *Nfkbia* and *Egr1*. The S2 subcluster highly expressed *Wnt16* and *Axin2*, and the S3 subcluster highly expressed *Mmp3*, *Adh1*, *Ces1d*, *Cxcl16*, *Rspo1* and *Rspo3*. Subclusters S4A and S4B represented ectocervical stromal cells; subcluster S4A showed preferential expression of *Sfrp1* and *Bmp4*, and subcluster S4B expressed *Dkk2* and *Crabp1* (Fig. 3b,c).

**Extended Data Fig. 5:**
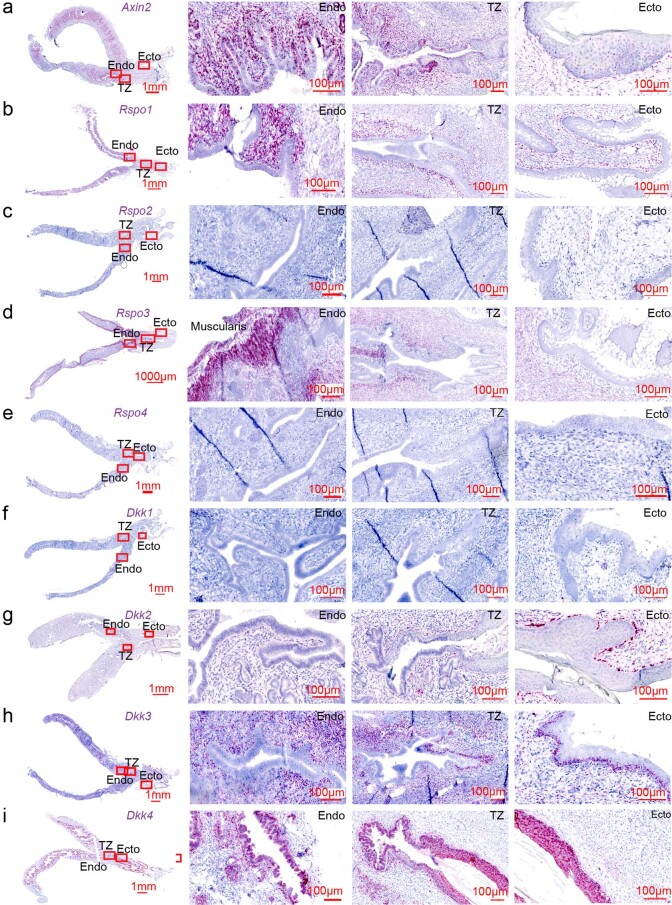
Microenvironmental signaling molecules in the cervix. Tiled brightfield images of entire mouse genital system sections labeled with smRNA-ISH for (**a**) *Axin2*, (**b**) *Rspo1*, (**c**) *Rspo2*, (**d**) *Rspo3*, (**e**) *Rspo4*, (**f**) *Dkk1*, (**g**) *Dkk2*, (**h**) *Dkk3*, and (**i**) *Dkk4*; nuclei in blue. Boxed areas are magnified on the right. Data representative of n = biologically independent experiments from 3 mice.

smRNA-ISH analysis showed that the spatial distribution of Wnt agonists and antagonists in the underlying stroma defined the boundary between the two epithelia, thus confirming the scRNA-seq data (Fig. 3d). *Rspo1* and its downstream target *Axin2* were highly expressed in the stroma beneath the columnar epithelium, and *Rspo3* was expressed in the endocervical muscularis (Fig. 3d and Extended Data Fig. 5a,b,d). Notably, the expression of the gene for the Wnt antagonist *Dkk2* was restricted to the stroma proximal to the ectocervical basal cells, while the squamous epithelium highly expressed *Dkk3* (Fig. 3d and Extended Data Fig. 5g,h). By contrast, in the endocervix, *Dkk3* expression was high throughout the stroma (Fig. 3d and Extended Data Fig. 5h). Expression levels of *Rspo2*, *Rspo4*, *Dkk1* and *Dkk4* did not show notable regional variation (Extended Data Fig. 5c,e,f,i). To further investigate the effects of DKK2 and DKK3, endocervical organoids were reseeded in medium containing WNT3A and RSPO1 plus DKK2 or DKK3, or both DKK2 and DKK3. The Wnt-deficient medium served as a control. The presence of DKK2 resulted in a substantial decrease in columnar organoid size, similar to that found in Wnt-deficient medium, confirming that DKK2 exerts an inhibitory effect. This was prevented when the medium additionally contained DKK3 (Fig. 3e). These findings corroborate earlier studies that, in contrast to other DKK members, DKK3 either has no effect on Wnt signalling or functions as a Wnt agonist^27–30^. Blocking Wnt signalling using inhibitor of Wnt production 2 (IWP2)^31,32^, suppressed endocervical organoid growth but did not affect ectocervical organoids (Fig. 3f,g). This indicates that squamous epithelium does not depend on cell-autonomous Wnt signalling. Moreover, there was no difference in the expression of the lineage-specific markers KRT5, KRT8 and p63 in columnar and squamous organoids upon treatment with DKK2 or IWP2 (Fig. 3h). Together, these data show that the epithelium of the cervix is maintained by two distinct stem cell populations whose fate is determined by opposing Wnt-signalling microenvironments, with a defined switch at the transition zone (Fig. 3i).

### Wnt antagonists, Notch and EGFR signalling maintain ectocervical stemness and differentiation

**Fig. 4:**
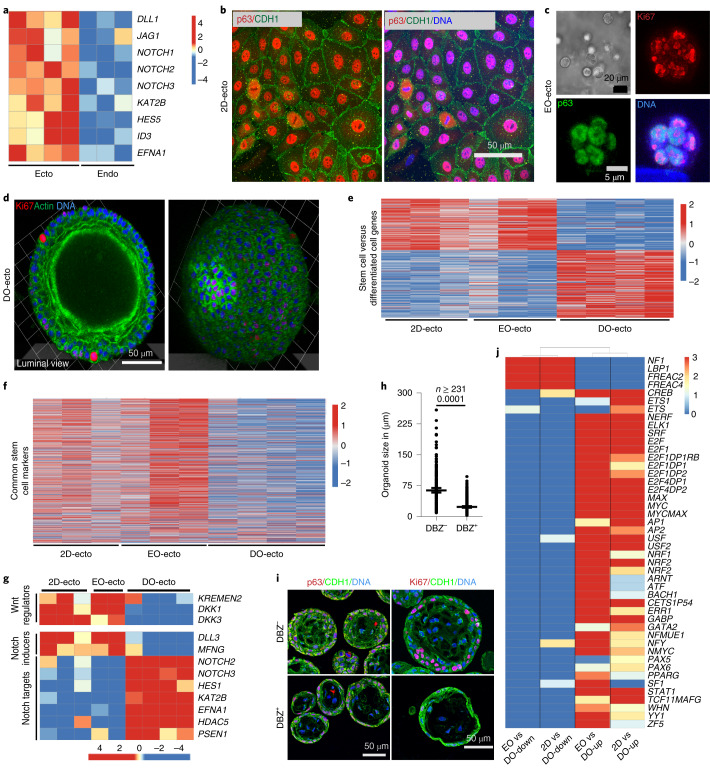
Stemness and differentiation of ectocervix depend on Wnt antagonist, Notch and EGFR signalling. **a**, Expression analysis of differentially regulated genes in human ecto- versus endocervical organoids. Notch-related genes are expressed at higher levels in the ectocervix. Columns represent individual donors. Colour bar represents z-scored gene expression. **b**, Confocal images of 2D human ectocervical stem cell cultures immunolabelled for p63 and CDH1. **c**,**d**, Three-dimensional reconstruction of whole-mount confocal images of three-day-old early ectocervical organoids (EO-ecto) labelled for p63 and Ki67 (**c**) and two-week-old differentiated ectocervical organoids (DO-ecto) labelled for Ki67 and actin (phalloidin) (**d**). Images in **b**–**d** are representative of biologically independent experiments from three donors. **e**,**f**, Heat maps of differentially regulated genes in 2D cultures and corresponding genes in EO-ecto and DO-ecto cultures (**e**) and genes frequently upregulated in stem cells (**f**). Colour bars denote z-scored gene expression. **g**, Heat map of selected differentially expressed genes, showing increased expression of Wnt regulators and Notch inducers in 2D cultures and EO-ecto cultures, compared with the expression of Notch activation-associated genes in DO-ecto cultures. Columns represent individual biological replicates. Colour bar represents z-scored gene expression. **h**, Quantification of the area of human ectocervical organoids grown in the presence or absence of γ-secretase inhibitor (DBZ). Data are mean ± s.e.m.; *n* is the number of organoids quantified from a representative of three biologically independent experiments. **i**, Confocal images of human ectocervical organoids immunolabelled for CDH1, Ki67 or p63. Inhibition of Notch activation by DBZ prevented differentiation and reduced proliferation. Images representative of biologically independent experiments from three donors. **j**, Heat map showing GSEA enrichment (–log_10_(*P*-value)) of genes that share *cis*-regulatory motifs for transcription factors (right). Among the genes upregulated in DO-ecto, there was an enrichment of genes regulated by transcription factors downstream of the RAS-antagonistic NF1 pathway. In 2D cultured cells and EO-ecto cultures, there was an enrichment of genes that are regulated by transcription factors downstream of Notch and EGFR–RAS–MAPK target genes. Colour bar represents the GSEA enrichment score. EO vs. DO-down indicates downregulated genes in EO-Ecto compared to DO-Ecto that are targets of shown transcription factors. EO vs. DO-up, indicates upregulated genes in EO-Ecto compared to DO-Ecto that are targets of shown transcription factors. Same rules apply for 2D vs. DO-down and 2D vs. DO-up. Statistical source data are provided in Source Data Fig. 4. Source data

**Extended Data Fig. 6:**
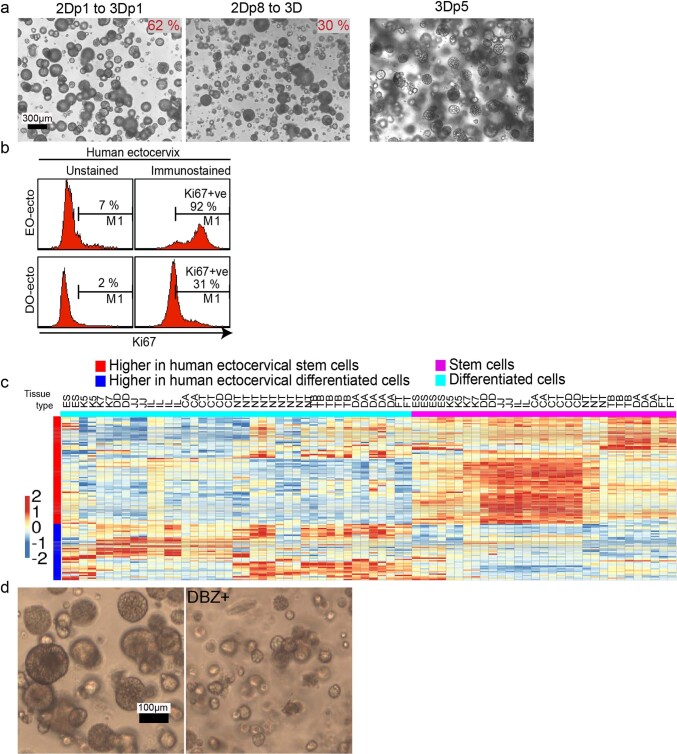
Ectocervical stemness and differentiation. **a**, Brightfield images of human organoids at P1 or P8 from 2D-ectocervical cells and long-term passage (P5) of organoids in 3D. Percentages indicate organoid-forming efficiency. **b**, Percentage of Ki67^+^ proliferating cells in EO-ecto and DO-ecto organoid-derived cells. **c**, Heatmap of genes concordantly up- or downregulated in ectocervical stem cells vs differentiated cells and similar comparisons to the ground state stem cell dataset (see Methods for details). Expression levels in stem cells and their corresponding differentiated cells from 13 different tissue types. DA-Distal Airway; NT-Nasotubular epithelium; TB-Tracheobronchial epithelium; FT-Fallopian tube; CA-Colon ascendens, CD-Colon descendens, CT-Colon transversum, DD-Duodenum, ES-Esophagus, IL-Ileum, JJ-Jejunum, K5-Keratin 5^+^ oesophagal cells, K7-Keratin 7^+^ oesophagal cells. **d**, Phase-contrast images of human ectocervical organoids in the presence or absence of DBZ. Data representative of n = biologically independent experiments from 3 donors.

Next, we sought to identify the mechanisms that control self-renewal and differentiation in human ectocervix. Microarray analysis showed a higher expression of Notch-related genes in ectocervical squamous organoids than in endocervical columnar organoids (Fig. 4a). To find out how differentiation is controlled, we performed a comparative analysis of 2D-grown ectocervical cells (2D-ecto), three-day-old early organoids (EO-ecto), and two-week-old mature organoids (DO-ecto). Cultures of 2D-ecto were positive for CDH1 and p63, and exhibited organoid-forming potential (Fig. 4b and Extended Data Fig. 6a). EO-ecto consisted of 8–16 undifferentiated cells positive for Ki67 and p63 (Fig. 4c and Extended Data Fig. 6b). DO-ecto consisted of several stratified layers, more than two-thirds of which were differentiated cells, and the rest were proliferating cells (Fig. 4d and Extended Data Fig. 6b). Gene expression patterns of 2D-ecto and EO-ecto showed high similarity and displayed a set of differentially expressed genes compared with DO-ecto (Fig. 4e and Supplementary Table 5–8). We performed a comparative analysis with a stem cell signature (that is, frequently upregulated genes in stem cells from diverse tissues^33^). The results confirmed that the expression profiles of early cells (2D-ecto and EO-ecto) showed a high similarity to this stem cell signature, but mature DO-ecto did not (Fig. 4f and Supplementary Table 6–9). This was further supported by comparisons with expression profiles of ground-state stem cells versus their respective differentiated cells (Extended Data Fig. 6c and Supplementary Tables 6–8 and 10). Thus, we conclude that cells from 2D-ecto and EO-ecto correspond to ectocervical stem cells.

A survey of genes upregulated in ectocervical stem cells versus differentiated cells revealed high expression of the Notch ligands delta-like ligand 3 (*DLL3*) and manic fringe (*MFNG*) (Fig. 4g). By contrast, differentiated cells expressed higher levels of *NOTCH2* and *NOTCH3* receptors and their targets, including the transcription factor *HES1* and presenilin 1 (*PSEN1*), a core component of γ-secretase (Fig. 4g). Ectocervical stem cells also expressed *DKK1* and its receptor *KREMEN2* (Fig. 4g). Accordingly, blocking Notch activation using the γ-secretase inhibitor DBZ reduced growth of organoids (Fig. 4h and Extended Data Fig. 6d), which failed to differentiate and stratify (Fig. 4i). Thus, it appears that the ectocervical stem cells act as a source of Notch signals, whereas the differentiated cells act as Notch signal-receiving cells, a trans-activating interaction that facilitates differentiation and epithelial stratification.

Further, gene set enrichment analysis (GSEA) revealed that genes regulated by transcription factors downstream of Notch and EGFR–RAS–MAPK signalling, were highly enriched in ectocervical stem cells, including targets of the transcription factors AP1, CREB, ETS, NERF, ELK1, E2F, SRF, MYC and YY1^34–37^ (Fig. 4j). These two pathways function together to regulate proliferation and differentiation^38–40^, in congruence with the essential role of EGF in the formation of stratified organoids (Fig. 2b and Extended Data Fig. 3a). Conversely, genes belonging to the RAS-antagonistic NF1 pathway^41^ were enriched in differentiated cells. Together, these observations indicate that Wnt antagonists, along with EGFR and Notch-inducing pathways, regulate ectocervical stemness and differentiation.

### Remodelling of the stromal compartment drives the emergence of KRT5^+^ squamous metaplasia

**Extended Data Fig. 7:**
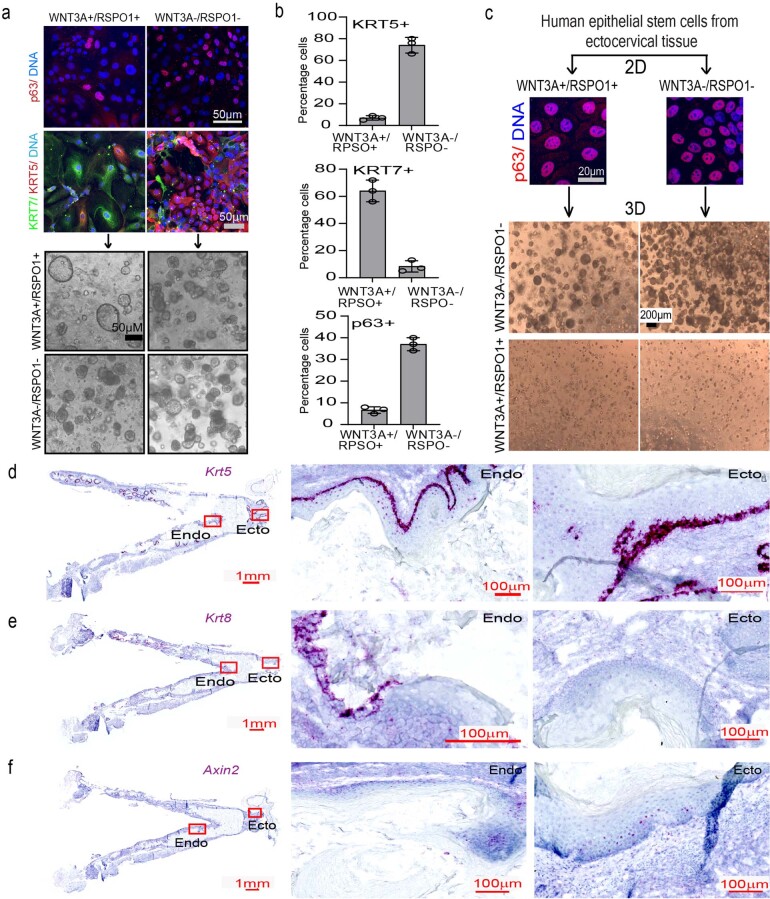
Regulation of squamous metaplasia by the tissue microenvironment. Regulation of squamous metaplasia by the tissue microenvironment. **a-b**, WNT-deficient medium enriches for p63^+^/KRT5^+^ human endocervical cells, grown in 2D, that can only give rise to stratified organoids. WNT-proficient medium results in predominantly KRT7^+^ and few p63^+^/KRT5^+^ cells, which can give rise to columnar or stratified organoids, respectively, depending on culture conditions. **b**, Bar graph depicting the number of cells positive for KRT7, p63 or KRT5. Data represented as mean ± s.e.m from biologically independent experiments of 3 donors. **c**, Confocal images of human ectocervical epithelial cells grown in 2D. p63^+^ cells are present in WNT-deficient medium as well as in WNT-proficient medium, but their growth is reduced. However, organoids are formed only in WNT-deficient medium. **d-f**, Tiled brightfield images of entire female genital system sections from mice fed vitamin A-deficient diet. smRNA-ISH labelling for *Krt5* (**d**), *Krt8* (**e**) and *Axin2* (**f**); nuclei in blue. Boxed areas are magnified on the right. Data representative of n = biologically independent experiments from 3 mice or human samples. Statistical source data are provided in Source Data Extended Data Fig. 7. Source data

Having illuminated the signalling pathways and cellular components involved in stemness and differentiation of the ectocervical squamous epithelium, we next examined their role in the emergence of squamous metaplasia. When primary human endocervix-derived cells were grown in 2D in a Wnt-proficient medium, such cultures contained only a few KRT5^+^ or p63^+^ cells; after a transfer to organoid conditions with the same medium, they gave rise to columnar organoids (Extended Data Fig. 7a,b). However, these 2D cells, when cultured in a Wnt-deficient medium, showed a clear enrichment for KRT5^+^ and p63^+^ cells. After a transfer to organoid conditions, these cells produced only squamous organoids, including the characteristic basal and parabasal p63^+^ cells, even in the presence of WNT3A and/ RSPO1 (Extended Data Fig. 7a,b). By contrast, human ectocervical cells that were first grown as 2D in either Wnt-proficient or -deficient medium gave rise only to squamous organoids after a transfer to a Wnt-deficient medium (Extended Data Fig. 7c). Together, this indicates the presence of squamous stem cells in the endocervix that can give rise to KRT5^+^p63^+^ cells given a suitable microenvironment.

**Fig. 5:**
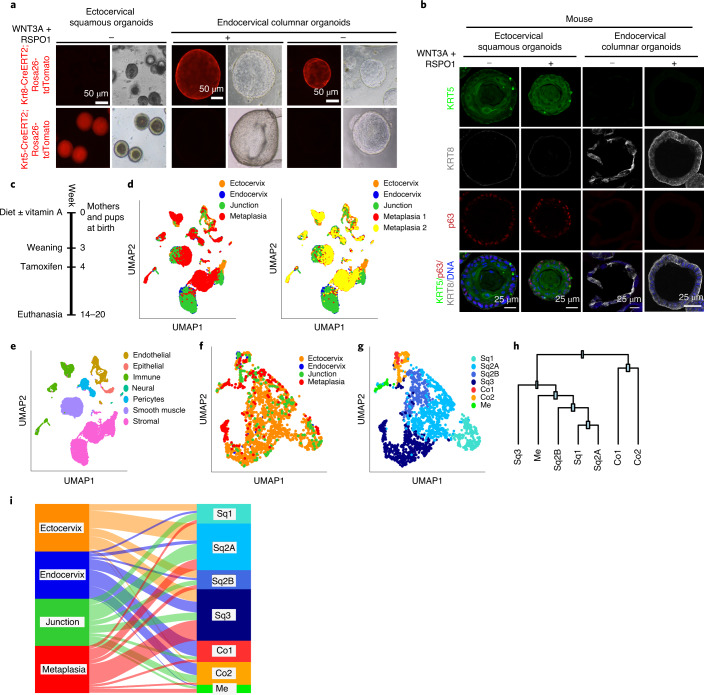
Two distinct stem cells from the endocervix give rise to columnar or squamous stratified lineages depending on the microenvironment. **a**, Organoids derived from lineage-traced *Krt8-Cre*^*ERT2*^*;Rosa26-tdTomato* and *Krt5-Cre*^*ERT2*^*;Rosa26-tdTomato* mice were grown in Wnt-proficient or -deficient medium. Data are representative of biologically independent experiments from three mice. **b**, Cells isolated from the ecto- and endocervix of tamoxifen-treated *Krt8-Cre*^*ERT2*^*;Rosa26-tdTomato* mice were grown in Wnt-proficient or -deficient medium to form organoids. Confocal images of organoids immunolabelled for KRT8, KRT5 and p63; nuclei in blue. Data are representative of biologically independent experiments from three mice. **c**, Treatment scheme for the vitamin A-deficient diet lineage-tracing study in mice. **d**–**i**, Combined scRNA-seq analysis of datasets derived from healthy mouse ectocervix, endocervix, transition zone and endocervix tissues with squamous metaplasia induced by vitamin A-deficient diet. **d**, UMAP of the endocervix, ectocervix, transition zone and metaplasia, as indicated by colours (left). Metaplasia samples were acquired in two independent technical replicates to check for batch effects—both replicates (1 and 2) have been projected on the UMAP (right). **e**, UMAP of cellular subclusters; single cells are colour coded by cluster annotation. **f**,**g**, UMAP of epithelial subclusters colour coded by the tissue of origin (**f**) or by cluster annotation (**g**). **h**, Phylogenetic tree of subclusters indicating inter-cell distances between the average value of cells in each subcluster in terms of gene expression space. **i**, Sankey diagram showing the contribution of epithelial cells from each tissue type to the subclusters shown in **g**. Colours and labels indicate sampled tissue as in **f** (left) and subclusters as in **g** (right).

We aimed to test whether the squamous and columnar organoids originate from distinct lineage-specific stem cells, or rather a transdifferentiation of columnar to squamous epithelial cells occurs in the absence of Wnt factors. We induced lineage tracing in *Krt5-Cre*^*ERT2*^*;Rosa26-tdTomato* and *Krt8-Cre*^*ERT2*^*;Rosa26-tdTomato* mice. Five weeks later, epithelial cells were isolated from the ecto- and endocervix of both genotypes and grown as organoids in Wnt-deficient or -proficient media. Endocervical organoids derived from *Krt8-Cre*^*ERT2*^*;Rosa26-tdTomato* mice were found to be labelled, whereas matched ectocervical squamous organoids were not. However, when grown in Wnt-deficient medium, endocervical lineage-labelled cells did not give rise to any labelled KRT5^+^p63^+^ squamous organoids, excluding transdifferentiation ability of these cells (Fig. 5a,b).

Conversely, ectocervical squamous organoids derived from *Krt5-Cre*^*ERT2*^*;Rosa26-tdTomato* mice were labelled, whereas matched columnar endocervical organoids were not (Fig. 5a). Our results show that the postnatal columnar and stratified cervical epithelia are derived from two different lineages, each of which respond to a particular microenvironment. Changing the Wnt signals does not induce the transdifferentiation of columnar cells to the stratified cells or vice versa.

**Fig. 6:**
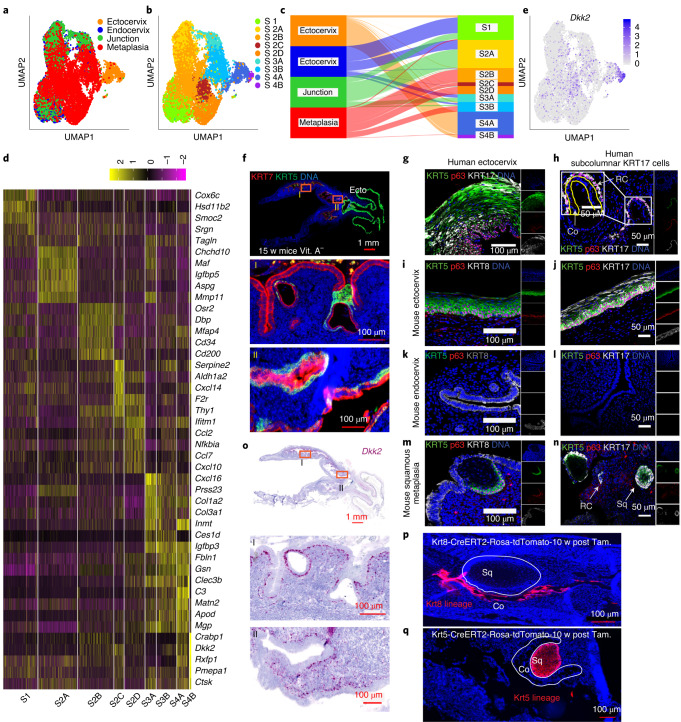
Endocervical stroma undergoes extensive remodelling during metaplasia. **a**–**e**, Combined scRNA-seq analysis of datasets derived from healthy mouse ectocervix, endocervix, transition zone and endocervix tissues with squamous metaplasia induced by vitamin A-deficient diet. UMAP of stromal subclusters coloured by sampled tissue (**a**) or cluster annotation (**b**). **c**, Sankey diagram showing the contribution of stromal cells from each tissue type to the clusters shown in **b**. Colours and labels for sampled tissue as in **a** (left) and subclusters as in **b** (right). **d**, Heat map of the top 5 genes expressed in each stromal subcluster shown in **b**. Colour bar denotes fold change from mean gene expression across all samples. **e**, Normalized expression values of *Dkk2* on the UMAP. **f**, Tissue sections from the genital system of a mouse fed with a vitamin A-deficient diet for 15 weeks; labelling with antibodies against KRT7 and KRT5. Middle and bottom: magnified view of boxed regions, showing an outgrowth of subcolumnar KRT5^+^ stem cells that gave rise to a squamous metaplastic epithelium in the endocervix. Boxed areas labelled as I and II are magnified at the bottom. **g**,**h**, Confocal images showing KRT17, p63 and KRT5 in human ectocervical stratified epithelial tissue (**g**) and subcolumnar cells in endocervix (**h**); RC indicates reserve cells, nuclei in blue. **i**–**l**, Confocal images of cells positive for KRT8, p63, KRT5 or KRT17 in mouse ectocervical stratified epithelium (**i**,**j**), mouse endocervical tissue (**k**,**l**) and mouse endocervix with squamous metaplasia (**m**,**n**); nuclei in blue. **o**, smRNA-ISH of tissue from a mouse fed with a vitamin A-deficient diet. The expression of *Dkk2* is enhanced in the endocervical stroma. Boxed areas labelled I and II are magnified (middle and bottom). **p**,**q**, Lineage tracing in *Krt8-Cre*^*ERT2*^*;Rosa26-tdTomato* (**p**) and *Krt5-Cre*^*ERT2*^*;Rosa26-tdTomato* (**q**) mice fed with a vitamin A-deficient diet revealed that squamous metaplasia arising in the endocervix is negative for Krt8-tdTomato (**p**) and positive for Krt5-tdTomato (**q**) lineage markers. Data shown in **f**–**q** are representative of biologically independent experiments from three mice or human samples. Tiled images shown in **f**,**o**–**q** were acquired with an AxioScan imager.

To analyse metaplasia development in vivo, we used a model of mice fed with a vitamin A-deficient diet, in which abnormal foci of squamous metaplasia develop in the endocervix^13^ (Fig. 5c). To identify changes in the epithelial and stromal compartments, we performed scRNA-seq and clustering analysis of cells isolated from the endocervix, ectocervix, transition zone and endocervix metaplasia (that is, metaplastic foci) (Fig. 5c–e and Supplementary Table 11). The epithelial populations were assigned to the Sq, Co and Me subclusters (Fig. 5g and Supplementary Table 12), as done for the healthy tissue (Fig. 1c). A phylogenetic tree, generated on the basis of the expression profiles of each epithelial subcluster, revealed that the squamous subpopulations (Sq1, Sq2A, Sq2B and Sq3) and Me cells (Me) were transcriptionally similar but distinct from the columnar subpopulations (Co1 and Co2), as shown by the distance between nodes (Fig. 5h). We next generated a Sankey diagram to analyse the transcriptional contribution of epithelial cells from each tissue region to different subclusters. The endocervix metaplasia demonstrated amplification of expression profiles similar to the squamous and myoepithelial types compared with a healthy endocervix (Fig. 5i). To characterize the alterations in the stromal population, we performed unsupervised clustering of stromal cells from the endocervix, ectocervix, transition zone and endocervix metaplasia (Fig. 6a), and defined stromal subclusters according to gene expression (Fig. 6b and Supplementary Table 13). The stromal compartment of endocervix metaplasia had a distinct clustering profile from those of the healthy endocervix, ectocervix and transition zone (Figs. 3a and 6b). In agreement, a Sankey diagram revealed the emergence of distinct stromal cell subclusters in the endocervix metaplasia (S2B, S2C and S2D) compared with healthy endocervix and ectocervix (Fig. 6c). We also observed an increased expression of *Dkk2* in a subcluster of the endocervix metaplasia (Fig. 6a,b,d,e), which primarily corresponds to the ectocervical S4B in healthy mice. These data indicate an extensive remodelling of the stroma during metaplastic development.

To further consolidate the lineage properties of stratified and columnar epithelia and spatial changes of the cervical microenvironment during metaplasia, we performed lineage tracing, smRNA-ISH and immunohistochemistry in our metaplasia mouse model. The mice exhibited upregulation of *Dkk2* in the stroma of the endocervix and uterine horns (Fig. 6e,o compared with Fig. 3d and Extended Data Fig. 5g). We observed an emergence of subcolumnar KRT5^+^, KRT17^+^ and p63^+^ cells, which were negative for KRT8 (Fig. 6f–n and Extended Data Fig. 7d). These cells were similar to healthy ectocervical squamous and endocervical subcolumnar reserve cells, which appeared to develop into a metaplastic stratified epithelium. *Axin2*, which is typically expressed in the endocervix, remained unaltered and expression of KRT8 and KRT7 was restricted to the columnar epithelium (Fig. 6f,k–m and Extended Data Fig. 7e,f). By inducing lineage tracing in *Krt5-Cre*^*ERT2*^*;Rosa26-tdTomato* and *Krt8-Cre*^*ERT2*^*;Rosa26-tdTomato* mice fed with a vitamin A-deficient diet, we supported our notion that KRT5^+^ cells gave rise to the endocervix-localized squamous metaplasia, whereas KRT8^+^ cells gave rise to the columnar epithelium as in healthy mice (Fig. 6p,q). We conclude that while Wnt agonists support formation of columnar epithelium, the local upregulation of the Wnt antagonist DKK2 in the stroma drives the proliferation of KRT5^+^ reserve cells, resulting in squamous metaplasia.

### Gene expression patterns of cervical SCC and ADC correlate with squamous and columnar lineage organoid signatures

**Fig. 7:**
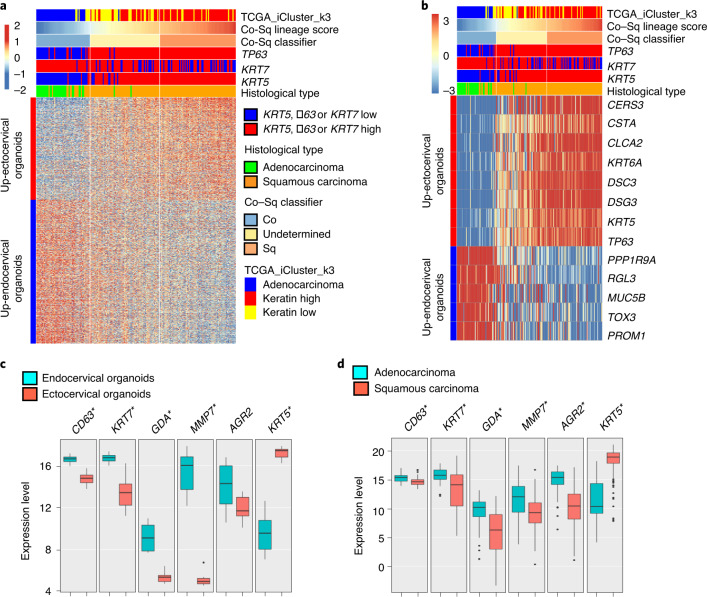
Transcription profiles of cervical SCCs and ADCs correlate with squamous and columnar epithelial lineages. **a**, Gene expression profiles of SCC and ADC correlate well with genes differentially expressed between ecto- and endocervical organoids, respectively. Colour bar denotes z-scored gene expression. **b**, Heat map showing the mean-substracted expression for selected bimodal genes in cancer samples that were differentially expressed in squamous and columnar organoids. Colour bar denotes fold change from mean gene expression across all samples. In **a**,**b**, Up-ectocervical organoids denote genes that are upregulated in ectocerival organoids compared to endocervical organoids; Up-endocervical organoids denote genes that are upregulated in endocerival organoids compared to ectocervical organoids. **c**,**d**, Gene expression profiles of proposed SCJ markers together with *KRT5* in cervical organoids (**c**) and 178 cervical cancer samples (**d**). Expression of these markers was higher in endocervical organoids (*n* = 6) and ADCs (*n* = 34) compared with ectocervical organoids (*n* = 10) and SCCs (*n* = 144), in contrast to *KRT5* expression; Box hinges correspond to first and third quartiles, the centre line corresponds to the median value, and whiskers correspond to the largest or smallest value within 1.5× the interquartile range from the hinges. All other outlying values are shown as individual points. Statistical significance was determined by a two-sided Mann–Whitney *U* test with no adjustments. *P < 0.001, except for *AGR2* in **c** (*P* = 0.073).

**Extended Data Fig. 8:**
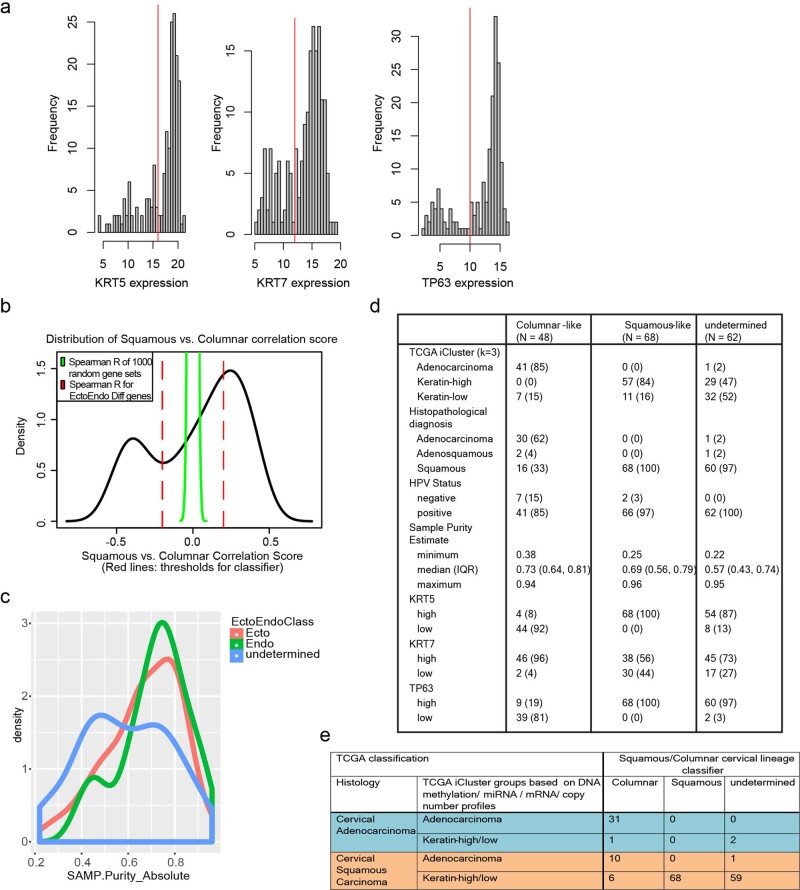
Endo- and ectocervical lineage markers as improved cancer classifiers. **a**, Distribution of mRNA expression values and selected thresholds used to define high and low *KRT5*-*, KRT7*- and *TP63*-expressing cancer samples. **b**, Distribution of Squamous-Columnar correlation scores used for the classification of cancer samples as squamous-like (> 0.2), columnar-like (< -0.2) or undetermined (-0.2 to 0.2). Dotted line: distribution of scores for the same samples with 1,000 random gene sets. **c**, Density plot showing estimated cancer cell purity for samples designated as columnar-like, squamous-like and the remaining undetermined samples. **d**, Summary table characterizing samples designated as columnar-like, squamous-like and remaining cases. Given are the numbers and percentages for each type. **e**, Classification of cancer samples according to histopathological diagnosis, TCGA iCluster classes as well as similarity to squamous and columnar lineages as proposed in this work.

Adult stem cells are susceptible to transformation and often constitute the cells of origin for various cancers^42^. To date, it is unclear from which cell types ADC and SCC originate. Thus far, their possible origin has been ascribed to phenotypic markers that characterize physiological cell types^19,43–46^. We used the gene expression signatures of squamous and columnar cervical organoids to determine the possible cells of origin of cervical cancers. We retrieved publicly available gene expression data—from The Cancer Genome Atlas (TCGA) study^47^—of 178 cervical cancers, including tumour classes based on an integrated clustering of mRNA, miRNA, genomic copy number and methylation data with iCluster, which divides cervical cancers into ADC and keratin-high and keratin-low SCC groups, in which the keratin-low SCC group shows a lower expression of squamous-lineage-specific markers^47^. We selected genes that were differentially expressed between ectocervical squamous and endocervical columnar organoids to classify the cancer samples into squamous-like and columnar-like. We found an agreement between cancers classified as columnar-like or squamous-like and their histological classification as ADC or SCC, respectively; this was also evident from comparison with the TCGA clusters of ADC or keratin-high and keratin-low groups (Fig. 7a and Extended Data Fig. 8d,e). A group of cases with a low tumour content, which showed no apparent similarity with either ectocervical or endocervical signatures, were classified as undetermined (Fig. 7a, Extended Data Fig. 8c and Supplementary Table 14).

Nevertheless, 59 out of 62 of the undetermined cases fell in the TCGA-defined keratin-low or keratin-high SCC clusters. Notably, cancer samples classified as columnar-like were mainly *KRT5*^low^, *KRT7*^high^ and *TP63*^low^, whereas samples classified as squamous-like and undetermined were mainly *KRT5*^high^ and *TP63*^high^ with a mixed *KRT7* status (Fig. 7a and Extended Data Fig. 8d,e). This suggests that the undetermined group may consist of SCCs within or outgrown into the endocervix, leading to the presence of contaminating *KRT7*^+^ endocervical columnar cells in the samples.

**Fig. 8:**
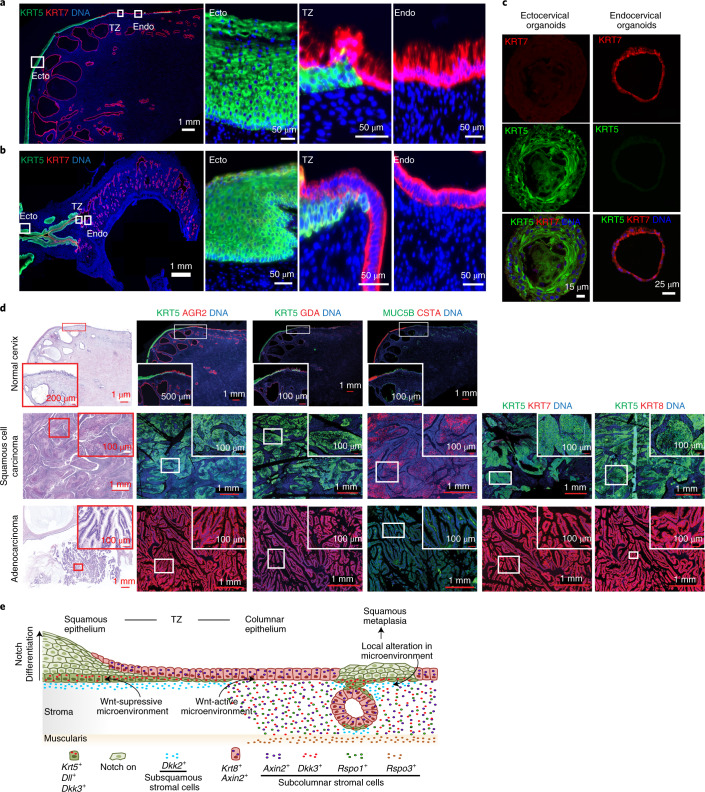
Molecular expression patterns of lineage markers in healthy tissue, organoids and cervical cancers. **a**,**b**, Left: tiled images of cervix tissue sections from human (**a**) and mouse (**b**), including stratified and columnar epithelium and the transition zone, immunolabelled for KRT5 and KRT7; nuclei in blue. Boxed areas are magnified on the right. Images are representative of biologically independent experiments from three mice or human samples. **c**, Confocal images of human ectocervical stratified and endocervical columnar organoids immunolabelled for KRT5 and KRT7; nuclei in blue. Images representative of *n* = 3 donors. **d**, Labelling for bimodally-expressed proteins in the normal cervix, SCC and ADC. Tissue sections from normal tissue, SCC and ADC of the cervix were stained with haematoxylin and eosin or labelled with antibodies against KRT5, KRT7, KRT8, AGR2, GDA, MUC5B and CSTA; nuclei in blue. Magnifications of the boxed areas are shown in insets. Data representative of biologically independent experiments from five humans. **e**, Model depicting the two epithelial lineages and Wnt–Notch microenvironment in the transition zone and during squamous metaplasia.

**Extended Data Fig. 9:**
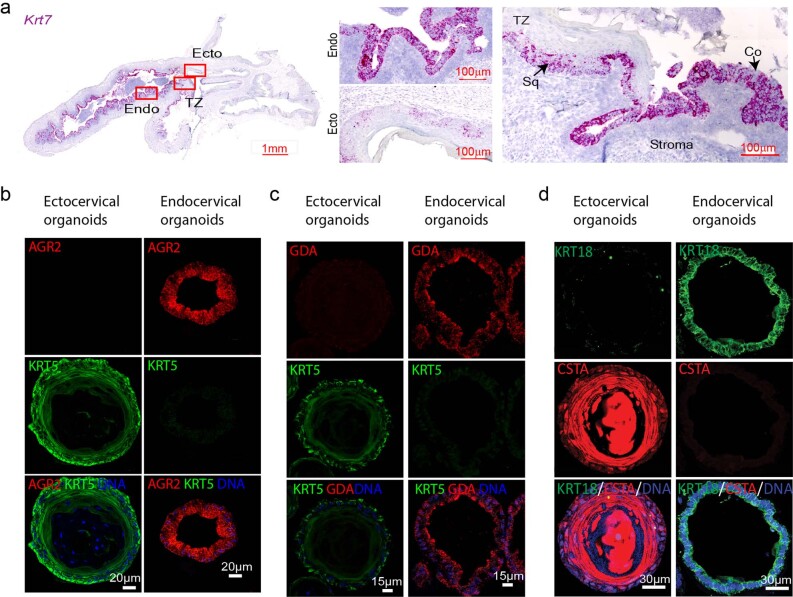
Expression of KRT7 and lineage-specific markers. **a**, Tiled brightfield images of sections showing the entire mouse genital system labelled with smRNA-ISH for *Krt7*. Boxes are magnified on the right. Data representative of n = biologically independent experiments from 3 mice. **b-d**, Confocal images of human ectocervical stratified and endocervical columnar organoids immunolabeled for AGR2, GDA, KRT18, KRT5 and CSTA; nuclei in blue. Images are representative of biologically independent experiments from 3 humans.

It was suggested that a small cell population located in the transition zone (called SCJ cells) expresses KRT7, CD63, GDA, AGR2 and MMP7 and that these cells are the precursors of both SCC and ADC^19,48^. We thus investigated the gene expression levels in organoids and TCGA cervical cancer samples, measuring the proposed SCJ markers^19^ as well as *KRT5*. The expression of these markers was significantly higher in endocervical organoids and ADC than in ectocervical organoids and SCC; the opposite trend was shown for *KRT5* (Fig. 7c,d). Immunohistochemistry and smRNA-ISH of human and mouse cervix tissue sections confirmed that KRT5 was expressed throughout the squamous epithelium. KRT7 was expressed throughout the columnar epithelium and, to a lesser extent, in the squamous epithelium, rather than being restricted to the transition zone alone (Fig. 8a,b and Extended Data Fig. 9a). Ectocervical organoids were positive for the squamous epithelial markers KRT5 and CTSA, whereas endocervical organoids were positive for the columnar epithelial markers KRT7, AGR2, GDA and KRT18 (Fig. 8c and Extended Data Fig. 9b–d). This implies that the reported SCJ cells are not distinct from the endocervical columnar lineage and are not the cells of origin for SCC.

In agreement with the notion that both keratin-high and keratin-low SCC cases derive from the squamous lineage, our analysis also revealed a set of differentially expressed genes between keratin-high and keratin-low, and ADC groups. We observed bimodal gene expression in columnar-like and squamous-like cancers, including *MUC5B, KRT5* and *CSTA*, that differentiates ADC from SCC (Fig. 7b). By contrast, the proposed SCJ markers KRT7, AGR2 and GDA exclusively labelled ADC but not SCC sections (Fig. 8d). In summary, the majority of cervical cancers can be divided into two groups on the basis of molecular signatures: SCCs most probably originate from the KRT5^+^ squamous lineage of the ectocervix, whereas ADCs most probably originate from the KRT7^+^KRT8^+^ columnar lineage of the endocervix.

## Discussion

Adult tissue homeostasis is maintained by niches established by complex interactions between stem cells and their surrounding microenvironment^49^. When tissue integrity is disturbed due to infections or other assaults, the disturbance is usually followed by substantial reorganizations of the niche, facilitating the restoration of tissue homeostasis^50^. The transition zones of the mucosal epithelium constitute critical zones of enhanced susceptibility to infections and carcinogenesis^51–54^. The cervical transition zone appears to be particularly vulnerable to HPV infections, the prime aetiological agent of cervical cancer. HPV is thought to infect the reserve stem cells at the transition zone, and the resulting dysregulation may lead to neoplasia^55^. Pre-existing metaplasia at the cervical transition zone may develop into neoplasia under the influence of HPV infection^10,11^. Thus, revealing the principles of niche homeostasis and its cellular composition is crucial to understanding the effects of intrinsic and extrinsic disturbances, including viral and bacterial infections.

In this study, we show that the cervical squamous and columnar epithelia arise from two distinct lineage-specific stem cell types and define the subpopulations of these lineages. Further, the endo- and ectocervix harbour distinct stromal subpopulations that constitute a microenvironmental gradient maintaining the transition zone. The regulation of stem cell maintenance, differentiation and tissue patterning by signalling gradients is a universal principle in development^56^, in which Wnt signalling is indispensable^20,57^. We demonstrate the presence of a Wnt gradient in the underlying stroma that selectively drives the columnar lineage while imposing quiescence on squamous-lineage-specific stem cells present in the same tissue. In squamous metaplasia, the endocervical stroma undergoes extensive remodelling. Here, we observed an upregulation of the Wnt inhibitor DKK2 in a stromal subpopulation. This transition to a Wnt-repressive microenvironment may activate quiescent squamous-lineage stem cells that invade the columnar epithelia at the transition zone or as metaplastic foci within the endocervix (Fig. 8e). Since oestrogen acts as a cofactor during HPV-driven cervical carcinogenesis^58^, an interplay between the oestrous cycle and Wnt signalling at the transition zone might occur and influence metaplasia and cancer development.

In concordance with previous studies^16–18^, we also found that subcolumnar KRT5^+^ cells are variably present at the transition zone in humans and mice. Furthermore, our data suggest these precursors of squamous metaplasia are present throughout the cervix and become activated in response to a Wnt-repressive environment. We also detected a divergent epithelial subpopulation in the cervix, classified as myoepithelium, which shares gene expression profiles with KRT5^+^ cells and myofibroblasts. These cells probably represent candidate precursors of the squamous lineage.

This study reveals a delineation of cervical ADC and SCC, implying that these cancers also originate from our two identified distinct epithelial lineages. Prophylactic ablation of the SCJ, where HPV infections and neoplasia are often observed, has been proposed to prevent neoplasia development^59^, but such efforts have failed to do so^59–61^. Our results suggest that this preventive ablation alone may not eliminate potential cervical cancer precursors, as new SCJs can develop upon activation of quiescent KRT5^+^ stem cells, which could present target sites for HPV infection and carcinogenesis. Furthermore, our organoid models reveal differences in the regulation of those two epithelial lineages similar to their respective cancers, thus providing an opportunity to study carcinogenesis and to identify specific therapeutic targets for each lineage. Nevertheless, organoids may not fully recapitulate the multifaceted interactions between various cell types and may thus represent an approximation of greater complexity in vivo.

From the molecular and cellular points of view, the present elucidation of the mechanisms that maintain cervical epithelial junctions provides an important conceptual advance. It suggests that homeostasis at these sites is not maintained by the transdifferentiation of one epithelial cell type to another. Instead, the adult tissue resembles a mosaic of different lineage-specific stem cell populations activated by the microenvironment in response to extrinsic or intrinsic signals. This concept of mucosal transition zone homeostasis fits well with other recent observations on mucosal stem cell identity^62^ and provides a basis for future investigations into transition zone homeostasis in other tissues with high clinical relevance.

## Methods

### Antibodies and chemicals

The following antibodies and chemicals were used: mouse anti-p63 (Abcam, ab375), rabbit anti-p63 (Abcam, ab53039), mouse anti-E-cadherin (BD Biosciences, 610181), rabbit anti-Ki67 (Abcam, ab16667), rat anti-Ki67–FITC (eBioscience, 11-5698), rabbit anti-KRT5 (Abcam, ab52635), rabbit anti-KRT5–Alexa488 (Abcam, ab193894), mouse anti-KRT7 (Santa Cruz, sc-23876), rabbit anti-KRT7 (Abcam, ab181598), rabbit anti-KRT7–Alexa555 (Abcam, ab209601), rabbit anti-CSTA (Sigma, HPA001031), rabbit anti-AGR2 (Proteintech, 12275-1-AP), mouse anti-MUC5B (Abcam, ab77995), rabbit anti-GDA (Sigma, HPA019352), Hoechst (Sigma, B2261), rabbit anti-KRT17 (Abcam, ab109725), mouse anti-KRT19 (Abcam, ab7754), mouse anti-KRT18 (Abcam, ab668), donkey anti-rabbit–Alexa Fluor 488 (Jackson Immuno Research, 711-546-152), donkey anti-rabbit–Cy3 (Jackson Immuno Research, 711-166-152), donkey anti-rabbit–Alexa Fluor 647 (Jackson Immuno Research, 647 711-605-152), donkey anti-mouse–Cy5 AffiniPure (Jackson Immuno Research, 715-175-151), Draq5 (Cell Signaling, 4085), γ-secretase inhibitor XX (DBZ) (Calbiochem 565789) and p38 inhibitor SB202190 (Sigma, S7067). Antibody concentrations and the link to validation by the provider are shown in Supplementary Table 15.

### Mice

All procedures involving animals were approved by the national legal, institutional and local authorities at Max Planck Institute for Infection Biology. This study is compliant with all relevant ethical regulations regarding animal research. All animals were maintained in autoclaved micro isolator cages and provided with sterile drinking water and chow ad libitum. Four- to twenty-week-old female mice were used for this study. Wild-type C57BL/6, *Krt5-Cre*^*ERT2*^ (ref. ^63^) and *Krt8-Cre*^*ERT2*^ (ref. ^64^) mice (Jackson Laboratory) were bred to *Rosa-tdTomato*^65^ mice to generate mice labelled in Cre-expressing cells. For KRT5^+^ or KRT8^+^ lineage analysis, Cre recombinase was induced in female mice by administering tamoxifen (Sigma) intraperitoneally (0.25 mg per g body weight in 50 μl corn oil) at 4 weeks of age for 3 consecutive days. Mice were euthanized at 14–20 weeks of age, and the genital tracts were removed for further analysis. Experiments were performed in at least three biological replicates per condition. Mice were randomly allocated to experimental groups in all experiments.

### Single-cell isolation and sequencing

Following FGT extraction, the endocervix, transition zone and ectocervix from control mice and endocervix from mice with metaplasia were cut out. Tissue samples were washed in sterile PBS (Gibco, 14190-094) and minced with scissors. Minced tissue was processed separately by incubating in 0.5 mg ml^−1^ collagenase II (Calbiochem, 234155) in a shaker (45 min, 37 °C). Tissue and dissociated cells were pelleted (7 min, 1,000*g*, 4 °C), the supernatant was discarded, cells were resuspended in warm TrypLE Express (Gibco, 12604021) and incubated in a shaker (15 min, 37 °C). The pellet was resuspended in Advanced DMEM/F-12 (ADF) medium (Invitrogen) and passed through a 40 µM cell strainer (BD Falcon, 352340). For single-cell sequencing, we combined cells isolated from three mice and processed each tissue separately. Cells were pelleted (7 min, 1,000*g*, 4 °C) and resuspended in PBS containing 0.04% w/v BSA (400 μg ml^−1^) at a concentration of 1,000 cells per μl. A 10x Chromium Controller was used to partition single cells into nanolitre-scale Gel-Bead-In-EMulsions (GEMs) and Single-Cell 3′ Reagent Kit v2 for reverse transcription, cDNA amplification and library construction according to the manufacturer’s protocol. Approximately 13,200 cells per sample were loaded onto the controller. A SimpliAmp Thermal Cycler (Applied Biosystems) was used for amplification and incubations. Libraries were quantified by Qubit^TM^ 3.0 Fluorometer (ThermoFisher), and quality was checked using a 2100 Bioanalyzer with a High Sensitivity DNA-kit (Agilent). Sequencing was performed in paired-end mode with S1 100-cycles kit using Novaseq 6000 sequencer (Illumina). Approximately 400 million reads were allocated per sample with at least 70,000 reads per cell.

### Computational analysis of scRNA-seq data

#### Cellranger pipeline

The Cell Ranger v3.0.1 software suite was obtained from 10x Genomics (https://support.10xgenomics.com/single-cell-gene-expression/software/downloads/latest). Raw sequencing data were first de-multiplexed and quality checked using the ‘mkfastq’ script. For all libraries, alignment and transcript quantification were performed with the standard ‘count’ script against the mm10 mouse genome assembly. All samples were aggregated using the ‘aggr’ script with the default normalization step (by downsampling) enabled. Only the healthy cervix samples were aggregated to form a control dataset under the same settings. The pre-filtered count matrices of the combined datasets were imported into R for further processing. A total of 25,932 cells were detected by Cell Ranger for all samples.

#### Quality control

The expression matrices were filtered, and potential doublets were removed by excluding barcodes with less than 250 genes, more than 4,000 genes, and more than 15,000 unique molecular identifiers. Barcodes with more than 10% of mitochondrial genes were excluded. Genes that were not detected for any barcode were removed. Potential doublets were scrutinized using Scrublet from the scran package (1.14.6)^66^, and marker gene expression was used to assess whether a hybrid transcriptome could mimic the cell type in focus; these analyses did not lead to further removal of cell-associated barcodes.

#### Clustering

Initial clustering was performed using the R package Seurat v2.3.4. Count data was log-normalized before identification of highly variable genes based on the following criteria: 0.0125 < mean of non-zero values < 4 and s.d. > 0.5. Unwanted variation due to library size and proportion of mitochondrial gene content was regressed out during data scaling. Features were selected using PCA: the first 21 principal components were selected for dimensional reduction and clustering of the whole datasets, 8 components were selected for the stromal subsets, and 6 components were selected for the epithelial subsets. A 2D representation was computed using the UMAP algorithm^67^. For clustering, a shared nearest neighbours graph was constructed using the Seurat functions BuildSNN without pruning and FindClusters using the SLM algorithm^68^. UMAPs (Fig. 1b and Extended Data Fig. 1a,b) were derived from analysing a total of 13,773 cells from ectocervix, endocervix and transition zone. From these, 1,626 epithelial cells (Fig. 1d and Extended Data Fig. 1c–h) and 6,380 stromal cells (Fig. 3a,c) were analysed. UMAPs (Fig. 5d,e) were derived from analysing a total of 22,590 cells from ectocervix, endocervix, transition zone and metaplasia samples. From these, 1,833 epithelial cells (Fig. 5f,g and Extended Data Fig. 1c–h) and 11,634 stromal cells (Fig. 6a–e) were analysed.

#### Cell cycle annotation

Cell cycle stage annotation was based on a list of cell cycle markers^69^. The Seurat function CellCycleScoring was used to compute quantitative scores for G2M- and S-phase and assign qualitative labels to each barcode.

#### Differential gene expression analysis

Differential expression analysis was performed between cell types/clusters using the FindAllMarkers function from the R package Seurat using default settings.

#### Batch-effect assessment

Technical variability was assessed by comparing the cell distribution across each technical batch within each transcriptional profile of assigned cell types using UMAPs. Cells derived from the endocervix of metaplasia mice were sequenced as two technical replicates, equally distributed across the identified clusters (Fig. 5d, right).

#### Further analysis

GO enrichment of cluster markers and differentially expressed genes was performed using the R package clusterProfiler. Dendrograms displaying the relationship of clusters were computed with the Seurat function BuildClusterTree.

### Vitamin A-deficient diet

From birth onwards, experimental mice and their mothers were fed with a vitamin A-deficient diet (SAFE, U8978P-0074) or a control diet with added Vitamin A at physiological levels (6 IU g^−1^, SAFE, U8978P-0075), following the described protocol^13^. Three-week-old littermates were weaned and maintained on the vitamin A-deficient or control diets for 14–20 weeks before being killed for further analysis.

### Mouse ecto- and endocervical medium

Endocervical cells were cultured in ADF medium (Invitrogen, 12634) supplemented with 12 mM HEPES, 1% GlutaMax, 1% B27, 1% N2, 50 ng ml^−1^ mouse EGF (Invitrogen, 15630-056, 35050-038, 17504-044, 17502048, PMG8043), 100 ng ml^−1^ mouse noggin, 100 ng ml^−1^ human FGF10 (Peprotech, 250-38-100, 100-26-25), 1.25 mM *N*-acetyl-l-cysteine, 10 mM nicotinamide, 10 µM ROCK inhibitor (Y-27632) (Sigma, A9165-5G, N0636, Y0503), 2 µM TGF-β receptor kinase inhibitor IV (Calbiochem, 616454), 1% penicillin–streptomycin (Gibco, 15140-122) with 25% WNT3A- and 25% R-spondin-1-conditioned medium. Ectocervical cells were cultured in endocervical medium but without 25% WNT3A- and 25% R-spondin 1-conditioned medium.

### Wnt-deficient human ectocervical medium

Ectocervical cells were cultured in ADF medium supplemented with 12 mM HEPES, 1% GlutaMax, 1% B27, 1% N2, 0.5 µg ml^−1^ hydrocortisone (Sigma, H0888-1G), 10 ng ml^−1^ human EGF (Invitrogen, PHG0311), 100 ng ml^−1^ human noggin, 100 ng ml^−1^ human FGF10 (Peprotech, 120-10 C, 100-26-25), 1.25 mM *N*-acetyl-l-cysteine, 10 mM nicotinamide, 2 µM TGF-β receptor kinase Inhibitor IV, 10 µM Y-27632, 10 µM forskolin (Sigma, F6886) and 1% penicillin–streptomycin.

### Wnt-proficient human endocervical medium

Endocervical cells were cultured in ADF medium supplemented with 12 mM HEPES, 1% GlutaMax, 1% B27, 1% N2, 10 ng ml^−1^ human EGF, 100 ng ml^−1^ human noggin, 100 ng ml^−1^ human FGF10, 1.25 mM *N*-acetyl-l-cystein, 10 mM nicotinamide, 2 mM TGF-β receptor kinase inhibitor IV and 10 µM Y-27632 with 25% WNT3A- and 25% R-spondin-1-conditioned medium.

### Epithelial stem cell isolation from human and mouse cervix

Human ecto- and endocervix samples were provided by the Department of Gynecology, Charité University Hospital, Berlin, Germany. Usage for scientific research was approved by their ethics committee (EA1/059/15); informed consent was obtained from all subjects. The study is compliant with all relevant ethical regulations regarding research involving human participants. Tissue biopsies from anonymous donors (Supplementary Table 16) were processed within 2–3 h after removal. Biopsies were sourced from standard surgical procedures. Mouse cervix was removed from euthanized 10- to 20-week-old female wild-type BALB/c mice (Charles River). Tissue samples were washed in sterile PBS (Gibco, 14190-094) and minced with surgical scissors. The minced human cervix tissue was incubated in 0.5 mg ml^−1^ collagenase II (Calbiochem, 234155) (2.5 h, 37 °C). Tissue and dissociated cells were pelleted (5 min, 1,000*g*, 4 °C), cells were resuspended in TrypLE Express (Gibco, 12604021) and incubated in a shaker (15 mins, 37°). Minced mouse cervix tissue was incubated in TrypLE express in a shaker (2.5 h, 37 °C). The pellet was resuspended in ADF medium and passed through a 40 µM cell strainer (BD Falcon, 352340). Cells were pelleted (5 min, 1,000*g*, 40 °C), resuspended in either human ecto- or endocervical medium or mouse cervical medium, and cultured either directly as organoids or as 2D cultures.

### Human epithelial 2D cell culture

Isolated human epithelial stem cells were resuspended in either ecto- or endocervical medium, plated and incubated in collagen-coated flasks. Upon 70–80% confluence, cells were detached using TrypLE Express and centrifuged (5 min, 1,000*g*, 40 °C). Then, cells were cultured as organoids or as stem cell-enriched 2D cultures. The 2D cultures were maintained by seeding 2D cells from P1 into flasks containing lethally irradiated J2-3T3 fibroblast feeder cells in ecto- or endocervical media. The medium was replaced, and irradiated fibroblasts added every 4 d until 60–70% confluence, at this stage, cells were reseeded onto freshly irradiated feeders or cryopreserved.

### Organoid culture and maintenance

Tissue-isolated or 2D culture cells were mixed with 50 µl ice-cold Matrigel (BD, 356231); Matrigel droplets were placed in prewarmed 24-well plates to allow polymerization (10 min, 37 °C). Freshly isolated endocervical cells were seeded at a higher density, as they had approximately 1% organoid-forming efficiency compared with 10% observed with ectocervical epithelial cells. The Matrigel droplet was then overlaid with 500 µl of prewarmed human or mouse ecto- or endocervical medium. Cultures were incubated for 2–3 weeks (37 °C, 5% CO_2_), and the medium was replaced every 4 d. For organoid passaging, organoids in Matrigel drops were first dissolved in 1 ml ice-cold ADF, thoroughly pipetted, transferred to 15 ml tubes, to which an additional 4 ml ice-cold ADF medium was added until the Matrigel was dissolved thoroughly, followed by centrifugation (5 min, 300*g*, 4 °C). Pelleted organoids were incubated in 1 ml TrypLE Express (30 min, 37 °C), followed by mechanical fragmentation with vigorous pipetting using fire-polished glass Pasteur pipettes to generate single cells. For organoid expansion, cells were reseeded into a fresh Matrigel (ectocervical organoids at 1:10 ratio and endocervical organoids at 1:5 ratio). Matrigel was allowed to polymerize as described above.

### Organoid-forming efficiency

Stem cells were counted, and a defined number was resuspended in 50 µl of Matrigel to generate organoids as described above. Two to three weeks later, whole-well images were taken and the number and area of formed organoids were determined using ImageJ to calculate the organoid-forming efficiency.

### Immunofluorescent histochemistry

Organoids were washed with cold PBS 5 times to remove Matrigel, fixed with 4% paraformaldehyde (1 h, room temperature) and washed twice in PBS. Organoids were then subjected to dehydration in an ascending ethanol series and placed in isopropanol and acetone (20 min each). Mouse and human tissues were washed in PBS and fixed with 4% paraformaldehyde (overnight, room temperature). Samples were dehydrated in an ascending ethanol series and placed in isopropanol and xylene (60 min each). Following paraffinization using a Leica TP1020 tissue processor, 5 µm sections were cut on a Microm HM 315 microtome. For immunostaining, paraffin sections were deparaffinized and rehydrated, treated with antigen-retrieval solution (Dako, S1699), and blocked using blocking buffer (1% BSA, 2% FCS in PBS) (1 h, room temperature). Sections were incubated with primary antibodies diluted in blocking buffer (90 min, room temperature), PBS-washed 5 times, incubated with secondary antibodies diluted in blocking buffer along with Hoechst or Draq5 (1 h, room temperature), washed with PBS 5 times, and finally mounted using Mowiol.

Fresh epithelial isolates were cultured in 2D on collagen-coated coverslips and fixed with 4% PFA (30 min, room temperature). Cells were permeabilized and blocked with 0.5% Triton X-100 and 1% BSA in PBS, incubated with primary antibodies diluted in 1% BSA in PBS (1 h, room temperature), washed with PSBT (PBS, 0.1% Tween-20) 3 times, incubated with secondary antibodies diluted in 1% BSA in PBS along with Hoechst or Draq5 (1 h, room temperature), washed with PBST three times, washed with PBS once, and finally mounted using Mowiol.

Images were acquired on a Leica TCS SP8 confocal microscope and processed with Adobe Photoshop; 3D reconstruction was done with Volocity 6.3 software (Perkin Elmer).

### Whole-mount staining

Matrigel was removed by extensive washing with ice-cold PBS (4 × 45 min). Organoids were allowed to settle by gravity to maintain their 3D structure, fixed using pre-warmed 3.7% PFA in PBS (1 h, room temperature), and washed with PBST 3 times. Permeabilization and blocking was performed using 5% donkey serum, 1% FCS, 0.05% Tween-20, 2% Triton X-100, 0.02% sodium azide in PBS (overnight, 4 °C). Organoids were incubated with primary antibodies diluted in blocking buffer (5% donkey serum, 1% FCS, 0.25% Triton X-100, 0.02% sodium azide in PBS) (3–5 d, 4 °C), washed with PBST (3 × 45 min, room temperature), incubated with secondary antibodies diluted in blocking buffer (2 d, 4 °C), washed with PBST (45 min) and washed with PBS containing 5% glycerol (3 × 45 min, room temperature). Organoids were then carefully transferred to ibidi µ-slides (81822) together with some PBS/glycerol solution. *Z*-stack images were acquired and processed as described.

### smRNA-ISH

Hybridizations of paraffin-embedded 10 µm tissue sections with RNAscope 2.5HD Red Reagent kit (Advanced Cell Diagnostics) were performed according to the manufacturer’s protocol, along with positive (PPIB) and negative (DapB) control probes. Tiled bright-field images were obtained with an AxioScan.Z1 tissue imager (Zeiss), processed with Zen 2.3 (Blue edition) and compiled with Adobe illustrator.

### RNA isolation and quality control for microarray analysis

Microarrays were hybridized for cultured cells and organoids. From Wnt-deficient medium: 2D human ectocervical cells in (three biological replicates from two donors and one technical replicate from one of the donors), human EO-ecto (three donors), human DO-ecto (four donors). From Wnt-proficient medium: 2D human endocervical cells (three donors) and human DO-ecto (three donors). Mouse EO-ecto and DO-ecto regardless of medium (two mice per condition). Effect sizes could not be pre-determined; therefore, sample sizes were selected on the basis of availability. Cells and organoids were pelleted and resuspended in 1 ml Trizol (Life Technologies), and RNA was isolated according to the manufacturer’s protocol. RNA was quantified with a NanoDrop 1000 UV-Vis spectrophotometer (Kisker), and quality was assessed by 2100 Bioanalyzer with RNA Nano 6000 microfluidics kit (Agilent Technologies).

### Microarray expression profiling

Single-colour hybridizations were performed on custom whole-genome human 8 × 60k arrays (Design ID048908) and feature extraction was applied to obtain probe intensities (Agilent). The extracted raw data were background-corrected, quantile-normalized and analysed for differential gene expression using R and the BioConductor package LIMMA (Supplementary Tables 6–8). R was used for all statistical analyses unless stated otherwise: unpaired tests were used for microarray gene expression comparisons and Mann–Whitney *U* test was used for comparisons with SCJ marker genes, *P* < 0.05. Microarray data have been deposited at the Gene Expression Omnibus (GEO) under accession number GSE87076. The signature of differentially expressed genes in cultured cells and organoids was based on all genes with false discovery rate <0.05 and log_2_(fold change) <−1.5 or >1.5 for each comparison (2D versus DO-ecto or EO-ecto versus DO-ecto). For each gene, the largest absolute fold change from both comparisons and possible replicate probes was taken.

### Analysis of stem cell-related genes

Raw microarray datasets of adult stem cells cultured on feeder cells and corresponding differentiated cells from an air–liquid interface, Matrigel or self-assembly sphere were downloaded from the GEO (GSE57584, GSE66115, GSE69453, GSE65013, GSE32606, GSE69429, GSE49292) and normalized using the RMA-sketch method (Affymetrix Power Tools). We assessed genes that were differentially expressed between stem cells and corresponding differentiated cell cultures: normal oesophagus, Barrett’s oesophagus, gastric cardia, duodenum, jejunum, ileum, colon ascendens, colon transversum, colon descendens, KRT5^+^ and KRT7^+^ fetal oesophagal cells, fallopian tube, nasal turbinated epithelium, tracheobronchial epithelium and distal airway epithelium. We selected significantly up- or downregulated stem cell-related genes (abs(log fold change) >1, adjusted *P* <0.05) in at least 5 out of 18 comparisons (Supplementary Tables 5, 9 and 10).

### Gene set enrichment analysis

We performed a pre-ranked analysis using GSEA software v2.1.0 and applied *t*-statistics on comparisons of ectocervical organoids (2D versus DO-ecto or EO-ecto versus DO-ecto) to rank probes and enrichment of MSigDB Motif gene sets (c3.all.v5.1.symbols.gmt) under standard settings; the Max_probe method (1,000 permutations) was applied for collapsing probe sets within genes. Motif gene sets (significant in at least 1 gene, FDR <5%) were further analysed. For the heat map visualization, we selected motif gene sets referring to the same transcription factors with the most significant negative log_10_(*P*-value).

### Cervical cancer data

Gene expression data, including public clinical and molecular annotations, were obtained for 178 unique samples published by The TCGA consortium. Gene expression levels were extracted from ‘*.rsem.genes.normalized_results’ files using custom scripts and normalized by applying DESeq2-generated size factors. To classify samples into squamous-like and columnar-like classes, gene expression levels were log_2_-transformed, and *Z*-scores were applied for comparisons. A squamous versus columnar organoid signature was defined on the basis of fold changes between ectocervical squamous and endocervical columnar organoids (2,834 genes, FDR < 0.05, absolute log_2_(fold change) > 1), selecting the probe with the lowest *P*-value for each gene. Spearman correlation coefficients (referred to as Co–Sq scores) were computed between *Z*-scored gene expression values from each cancer sample and the corresponding fold change for the same gene from the squamous versus columnar organoid signature. We defined samples with Co–Sq score >0.2 as squamous-like, those with Co–Sq score <−0.2 as columnar-like, and all others as ‘undetermined’ (Extended Data Fig. 8b). Applying the same procedure to 1,000 random gene sets of the same size and fold changes produced correlation coefficients generally lower than |0.06|. Thresholds for sample classification into *KRT5*^high^ versus *KRT5*^low^, *KRT7*^high^ versus *KRT7*^low^ and *TP63*^high^ versus *TP63*^low^ classes were selected manually to separate the highest cluster from all other samples (Extended Data Fig. 8a). For simplicity, we combined all diagnoses with an adenoma component (ADC and adenosquamous carcinoma) into cervical ADC (Supplementary Table 14).

### Statistics and reproducibility

GraphPad Prism (v.8) was used for statistical calculations and generation of plots. The data are displayed as mean ± s.e.m. *P* < 0.05 was considered to be statistically significant. Each experiment was repeated independently with similar results.

### Reporting Summary

Further information on research design is available in the Nature Research Reporting Summary linked to this article.

## Online content

Any methods, additional references, Nature Research reporting summaries, source data, extended data, supplementary information, acknowledgements, peer review information; details of author contributions and competing interests; and statements of data and code availability are available at 10.1038/s41556-020-00619-0.

## Supplementary information

Reporting Summary

Peer review information

Supplementary Table 1 Marker genes for annotated clusters of three healthy murine cervix datasets. Related to Fig. 1b. Marker genes (rows) associated with the population subsets (column 1) are extracted from Seurat (*P* < 0,01, column 2). Columns 3 to 6 give the default Seurat output (pct1 and pct2 refer to the percentage of cells expressing genes among the studied subset (pct1) and among the whole dataset analysed (pct2)). Ensembl gene number and gene names are given in columns 7 and 8, respectively. Statistical significance was calculated employing the Wilcoxon rank-sum test for genes detected for 10% of barcodes with at least 0.25-fold difference (log scale) across clusters using the Seurat function.

Supplementary Table 2 Marker genes for annotated clusters of the epithelial subsets from three healthy murine cervix datasets. Related to Fig. 1d. Marker genes (rows) associated with the population subsets (column 1) are extracted from Seurat (*P* < 0,01, column 2). Columns 3 to 6 give the default Seurat output (pct1 and pct2 refer to the percentage of cells expressing genes among the studied subset (pct1) and among the whole dataset analysed (pct2)). Ensembl gene number and gene names are given in columns 7 and 8, respectively. Statistical significance was calculated employing the Wilcoxon rank-sum test for genes detected for 10% of barcodes with at least 0.25-fold difference (log scale) across clusters using the Seurat function.

Supplementary Table 3 Gene Ontology categories for annotated clusters of the epithelial subset from three murine healthy cervix datasets. Related to Fig. 1f. Displayed GO terms for each subpopulation on Fig. 1f are labelled in red. Gene ontology (rows) associated to the epithelial subset from three murine healthy cervix datasets are calculated using clusterProfiler. For each distinct cluster (column 1, as identified in UMAP projection (Fig. 1d), GO terms (columns 2 and 3) are classified by decreasing *P*-value. The table reproduces the output of clusterProfiler (columns 3 to 7). Genes associated with each GO term are identified by their Entrez number (columm 8). Gene counts are given column 9. Statistical significance was calculated employing the Wilcoxon rank-sum test for genes detected for 10% of barcodes with at least 0.25-fold difference (log scale) across clusters using the Seurat function.

Supplementary Table 4 Marker genes for annotated clusters of the stromal subset from three murine healthy cervix datasets. Related to Fig. 3a. Marker genes (rows) associated with the population subsets (column 1) are extracted from Seurat (*P* < 0,01, column 2). Columns 3 to 6 give the default Seurat output (pct1 and pct2 refer to the percentage of cells expressing genes among the studied subset (pct1) and among the whole dataset analysed (pct2)). Ensembl gene number and gene names are given in columns 7 and 8, respectively. Statistical significance was calculated employing the Wilcoxon rank-sum test for genes detected for 10% of barcodes with at least 0.25-fold difference (log scale) across clusters using the Seurat function.

Supplementary Table 5 Differentially expressed genes from the heat maps and squamous versus columnar signature. Related to Fig. 4e. Statistical significance was calculated by two-sided moderated *t*-test adjusted for multiple testing by the method of Benjamini–Hochberg (FDR).

Supplementary Table 6 Genes differentially expressed in 2D versus differentiated organoids from human ectocervix. Related to Fig. 4e. Statistical significance was calculated by two-sided moderated *t*-test adjusted for multiple testing by the method of Benjamini–Hochberg (FDR).

Supplementary Table 7 Genes differentially expressed in early versus differentiated organoids from human ectocervix. Related to Fig. 4e. Statistical significance was calculated by two-sided moderated *t*-test adjusted for multiple testing by the method of Benjamini–Hochberg (FDR).

Supplementary Table 8 Genes differentially expressed in differentiated organoids from human ecto- and endocervix. Related to Fig. 4e. Statistical significance was calculated by two-sided moderated *t*-test adjusted for multiple testing by the method of Benjamini–Hochberg (FDR).

Supplementary Table 9 Differentially expressed genes from the heat maps and squamous versus columnar signature. Related to Fig. 4f. Statistical significance was calculated by two-sided moderated *t*-test adjusted for multiple testing by the method of Benjamini–Hochberg (FDR).

Supplementary Table 10 Differentially expressed genes from the heat maps and squamous versus columnar signature. Related to Extended Data Fig. 6c. Statistical significance was calculated by two-sided moderated *t*-test adjusted for multiple testing by the method of Benjamini–Hochberg (FDR).

Supplementary Table 11 Marker genes for annotated clusters of five healthy and metaplastic murine cervix datasets. Related to Fig. 5e. Marker genes (rows) associated with the population subsets (column 1) are extracted from Seurat (*P* < 0.01, column 2). Columns 3 to 6 give the default Seurat output (pct1 and pct2 refer to the percentage of cells expressing genes among the studied subset (pct1) and among the whole dataset analysed (pct2)). Ensembl gene number and gene names are given in columns 7 and 8, respectively. Statistical significance was calculated employing the Wilcoxon rank-sum test for genes detected for 10% of barcodes with at least 0.25-fold difference (log scale) across clusters using the Seurat function.

Supplementary Table 12 Marker genes for annotated clusters of the epithelial subsets from five healthy and metaplastic murine cervix datasets. Related to Fig. 5g. Marker genes (rows) associated with the population subsets (column 1) are extracted from Seurat (*P* < 0.01, column 2). Columns 3 to 6 give the default Seurat output (pct1 and pct2 refer to the percentage of cells expressing genes among the studied subset (pct1) and among the whole dataset analysed (pct2)). Ensembl gene number and gene names are given in columns 7 and 8, respectively. Statistical significance was calculated employing the Wilcoxon rank-sum test for genes detected for 10% of barcodes with at least 0.25-fold difference (log scale) across clusters using the Seurat function.

Supplementary Table 13 Marker genes for annotated clusters of the stromal subsets from five healthy and metaplastic murine cervix datasets. Related to Fig. 6b. Marker genes (rows) associated with the population subsets (column 1) are extracted from Seurat (*P* < 0.01, column 2). Columns 3 to 6 give the default Seurat output (pct1 and pct2 refer to the percentage of cells expressing genes among the studied subset (pct1) and among the whole dataset analysed (pct2)). Ensembl gene number and gene names are given in columns 7 and 8, respectively. Statistical significance was calculated employing the Wilcoxon rank-sum test for genes detected for 10% of barcodes with at least 0.25-fold difference (log scale) across clusters using the Seurat function.

Supplementary Table 14 Classification of cancer samples based on the expression signature of squamous ectocervical and columnar endocervical organoids.

Supplementary Table 15 Table of antibody dilutions and validation links.

Supplementary Table 16 Patient information.

## Data Availability

Microarray and scRNA-seq data that support the findings of this study have been deposited in the GEO under accession codes GSE87076 and GSE128987, respectively. Previously published microarray data that were reanalysed here are available under accession codes GSE57584, GSE66115, GSE69453, GSE65013, GSE32606, GSE69429 and GSE49292. The human cervical cancer data were derived from the TCGA research network (http://cancergenome.nih.gov/). The dataset derived from this resource that supports the findings of this study is available at https://gdc.cancer.gov/about-data/publications/cesc_2017. The quantitative data of this study are available within the paper and its supplementary information files. All other data supporting the findings of this study are available from the corresponding author on reasonable request. Source data are provided with this paper.

## Source data

Source Data Fig. 2 Statistical source data
Source Data Fig. 3 Statistical source data
Source Data Fig. 4 Statistical source data
Source Data Extended Data Fig. 4 Statistical source data
Source Data Extended Data Fig. 7 Statistical source data

## Code Availability

All computational codes used for analyses used can be accessed at https://github.com/MPIIB-Department-TFMeyer/Chumduri_Gurumurthy_et_al._Cervical_squamocolumnar_homeostasis.
