# Supplementary material for: Opposing Wnt signals regulate cervical squamocolumnar homeostasis and emergence of metaplasia
Source: Nat Cell Biol. 2021 Jan 18;23(2):184–97. doi: 10.1038/s41556-020-00619-0 (PMC7878191; doi:10.1038/s41556-020-00619-0)
Supplement: Supplementary file 1 — Reporting Summary [file 41556_2020_619_MOESM1_ESM.pdf]

nature research

Corresponding author(s): Thomas F Meyer and Cindrilla Chumduri

Last updated by author(s): Oct 31, 2020

Reporting Summary

Nature Research wishes to improve the reproducibility of the work that we publish. This form provides structure for consistency and transparency in reporting. For further information on Nature Research policies, see our Editorial Policies and the Editorial Policy Checklist.

Statistics

For all statistical analyses, confirm that the following items are present in the figure legend, table legend, main text, or Methods section.

n/a	Confirmed	
☐	☒	The exact sample size (*n*) for each experimental group/condition, given as a discrete number and unit of measurement
☐	☒	A statement on whether measurements were taken from distinct samples or whether the same sample was measured repeatedly
☐	☒	The statistical test(s) used AND whether they are one- or two-sided<br>*Only common tests should be described solely by name; describe more complex techniques in the Methods section.*
☒	☐	A description of all covariates tested
☐	☒	A description of any assumptions or corrections, such as tests of normality and adjustment for multiple comparisons
☐	☒	A full description of the statistical parameters including central tendency (e.g. means) or other basic estimates (e.g. regression coefficient) AND variation (e.g. standard deviation) or associated estimates of uncertainty (e.g. confidence intervals)
☐	☒	For null hypothesis testing, the test statistic (e.g. *F*, *t*, *r*) with confidence intervals, effect sizes, degrees of freedom and *P* value noted<br>*Give P values as exact values whenever suitable.*
☒	☐	For Bayesian analysis, information on the choice of priors and Markov chain Monte Carlo settings
☒	☐	For hierarchical and complex designs, identification of the appropriate level for tests and full reporting of outcomes
☐	☒	Estimates of effect sizes (e.g. Cohen's *d*, Pearson's *r*), indicating how they were calculated

Our web collection on statistics for biologists contains articles on many of the points above.

Software and code

Policy information about availability of computer code

Data collection	Microarray image data were analyzed and extracted with the Image Analysis/Feature Extraction software G2567AA v. A.11.5.1.1 (Agilent Technologies)<br>Next-generation sequencing data was collected by lllumina NovaSeq 6000 (lOx Chromium libraries).
Data analysis	Graphpad Prism version 8 was used for statistical analysis.<br>Gene set enrichment was performed using the GSEA software from<br>http://software.broadinstitute.org/gsea/downloads.jsp.<br>R-3.4 was obtained from https://cran.r-project.org/<br>R code used to produce analysis results from raw micro array files can be accessed under:<br>https://github.com/MPIIB-Department-TFMeyer/Chumduri_Gurumurthy_et_al._Cervical_squamocolumnar_homeostasis<br>GSEA software v2.l.0 was obtained from http://software.broadinstitute.org/gsea<br>Volocity 6.3 software package (Perkin Elmer)<br>Zen 2.3 (Blue edition) image analysis software<br>The Cell Ranger version 3.0.1 software suite (l0x Genomics) and Seurat Version 2.3.4 were used to analyse single cell sequencing data.<br>Adobe Photoshop and illustrator version 19

For manuscripts utilizing custom algorithms or software that are central to the research but not yet described in published literature, software must be made available to editors and reviewers. We strongly encourage code deposition in a community repository (e.g. GitHub). See the Nature Research guidelines for submitting code & software for further information.

Data

Policy information about availability of data

All manuscripts must include a data availability statement. This statement should provide the following information, where applicable:

- Accession codes, unique identifiers, or web links for publicly available datasets
- A list of figures that have associated raw data
- A description of any restrictions on data availability

Microarray and scRNA–seq data that support the findings of this study have been deposited in the Gene Expression Omnibus (GEO) under accession codes GSE87076 and GSE128987. Previously published microarray data that were re-analysed here are available under accession codes GSE57584, GSE66115, GSE69453, GSE65013, GSE32606, GSE69429, GSE4929219. The human cervical cancer data were derived from the TCGA Research Network: http://cancergenome.nih.gov/. The data-set derived from this resource that supports the findings of this study is available in https://gdc.cancer.gov/about-data/publications/cesc_2017.
The quantitative data of this study are available within the paper and its supplementary information files. All other data supporting the findings of this study are available from the corresponding author on reasonable request. Source data underlying the graphical representations in Figs. 2b,2c,2g, 3e,3f,3g, 4h, Ext. Data Fig 4g, Ext. Data Fig 7b are provided in the Source Data file.

Field-specific reporting

Please select the one below that is the best fit for your research. If you are not sure, read the appropriate sections before making your selection.

☒ Life sciences ☐ Behavioural & social sciences ☐ Ecological, evolutionary & environmental sciences

For a reference copy of the document with all sections, see nature.com/documents/nr-reporting-summary-flat.pdf

Life sciences study design

All studies must disclose on these points even when the disclosure is negative.

Sample size	No statistical methods were used to predetermine sample size. Sample size was based on the availability of the mice with respective genotype and previous experience. Experiments were performed on n = 3 biological replicates except where stated otherwise.
Data exclusions	No data were excluded from the experiments
Replication	All attempts at replication were successful. All graphs represent data with at least two biological replicates, all images represent findings reproduced at least twice in the laboratory.
Randomization	Animal experiments were done on littermates randomly allocated to different experimental groups. Human samples were allocated randomly to different experiments.
Blinding	The investigator was blinded for image analysis and for quantitative analysis.

Reporting for specific materials, systems and methods

We require information from authors about some types of materials, experimental systems and methods used in many studies. Here, indicate whether each material, system or method listed is relevant to your study. If you are not sure if a list item applies to your research, read the appropriate section before selecting a response.

Materials & experimental systems

n/a	Involved in the study	
☐	☒	Antibodies
☒	☐	Eukaryotic cell lines
☒	☐	Palaeontology and archaeology
☐	☒	Animals and other organisms
☐	☒	Human research participants
☒	☐	Clinical data
☒	☐	Dual use research of concern

Methods

n/a	Involved in the study	
☒	☐	ChIP-seq
☒	☐	Flow cytometry
☒	☐	MRI-based neuroimaging

Antibodies

Antibodies used	Mouse-anti-p63 (Abcam,# ab735)<br>Rabbit-anti-p63 (Abcam,# ab53039)<br>Mouse-anti-E-Cadherin (BD Biosciences,# 610181)<br>Rabbit-anti-Ki67 (Abcam,# ab16667)

Rat-anti-Ki67-FITC (eBioscience, # 11-5698)
Rabbit-anti-KRTS (Abcam,# ab52635)
Rabbit-anti-cytokeratin 5-Alexa488 (Abeam,# ab193894)
Mouse-anti-KRT7 (Santa Cruz,# sc-23876)
Rabbit-anti-cytokeratin 7 (Abcam,# ab181598)
Rabbit-anti-cytokeratin 7-Alexa555 (Abcam,# ab209601)
Rabbit-anti-CSTA (Cystatin A) (Sigma, #HPA001031)
Rabbit-anti-AGR2 (Proteintech, # 12275-1-AP)
Mouse-anti-MUCSB (Abcam, # ab77995)
Rabbit-anti-GDA (Sigma, #HPA019352)
Rabbit-anti-Cytokeratin 17 (Abcam, #ab109725)
Mouse-anti-Cytokeratin 19 (Abcam, #ab7754)
Mouse-anti-Cytokeratine 18 (Abcam, #ab668)
Donkey Anti-Rabbit- Alexa Fluor® 488 (Jackson Immuno Research, #711-546-152)
Donkey Anti-Rabbit -Cy™3 (Jackson Immuno Research, #711-166-152)
Donkey Anti-Rabbit - Alexa Fluor® (Jackson Immuno Research, #647 711-605-152)
Donkey Anti-Mouse - Cy™5 AffiniPure (Jackson Immuno Research, #715-175-151)

Validation

All antibodies are commercially available and validation experiments for the respective antibodies were performed by the commercial manufacturer and below we provide the respective link for each antibody:
Mouse-anti-p63
https://www.abcam.com/p63-antibody-4a4-ab735.html
Rabbit-anti-p63
https://www.abcam.com/p63-antibody-ab53039.html
Mouse-anti-E-Cadherin
https://www.bdbiosciences.com/us/applications/research/stem-cell-research/cancer-research/human/purified-mouse-anti-e-cadherin-36e-cadherin/p/610181
Rabbit-anti-Ki67
https://www.abcam.com/ki67-antibody-sp6-ab16667.html
Rat-anti-Ki67-FITC
https://www.thermofisher.com/antibody/product/Ki-67-Antibody-clone-SolA15-Monoclonal/11-5698-80
Rabbit-anti-KRT5
https://www.abcam.com/cytokeratin-5-antibody-ep1601y-cytoskeleton-marker-ab52635.html
Rabbit-anti-cytokeratin 5-Alexa488
https://www.abcam.com/cytokeratin-5-antibody-ep1601y-alexa-fluor-488-ab193894.html
Mouse-anti-KRT7
https://www.scbt.com/p/cytokeratin-7-antibody-rck105
Rabbit-anti-cytokeratin 7
https://www.abcam.com/cytokeratin-7-antibody-epr17078-cytoskeleton-marker-ab181598.html
Rabbit-anti-cytokeratin 7-Alexa555
https://www.abcam.com/cytokeratin-7-antibody-epr17078-alexa-fluor-555-ab209601.html
Rabbit-anti-CSTA (Cystatin A)
https://www.sigmaaldrich.com/catalog/product/sigma/hpa001031?lang=de&region=DE
Rabbit-anti-AGR2
https://www.ptglab.com/products/AGR2-Antibody-12275-1-AP.htm#tested-applications
Mouse-anti-MUC5B
https://www.abcam.com/muc5b-antibody-194e-ab77995.html
Rabbit-anti-GDA
https://www.sigmaaldrich.com/catalog/product/sigma/hpa019352?lang=de&region=DE
Rabbit-anti-Cytokeratin 17
https://www.abcam.com/cytokeratin-17-antibody-ep1623-cytoskeleton-marker-ab109725.html
Mouse-anti-Cytokeratin 19
https://www.abcam.com/cytokeratin-19-antibody-a53-ba2-cytoskeleton-marker-ab7754.html
Mouse-anti-Cytokeratine 18
https://www.abcam.com/cytokeratin-18-antibody-c-04-ab668.html
Rabbit-anti-Loricrin
https://www.abcam.com/loricrin-antibody-ab85679.html
Donkey Anti-Rabbit- Alexa Fluor® 488
https://www.jacksonimmuno.com/catalog/products/711-546-152
Donkey Anti-Rabbit -Cy™3
https://www.jacksonimmuno.com/catalog/products/711-166-152
Donkey Anti-Rabbit - Alexa Fluor® 647
https://www.jacksonimmuno.com/catalog/products/711-605-152
Donkey Anti-Mouse - Cy™5 AffiniPure
https://www.jacksonimmuno.com/catalog/products/715-175-151

Animals and other organisms

Policy information about studies involving animals; ARRIVE guidelines recommended for reporting animal research

Laboratory animals

For the study 4-20 week old female mice were used. The following genetic strains were used:
Wildtype C56BL/6
KRT5CreErt2/Rosa26-tdTomato
KRT8CreErt2/Rosa26-tdTomato

Wild animals	Study did not involve wild animals
Field-collected samples	No field collected samples were used
Ethics oversight	Landesamt für Gesundheit und Soziales (LaGeSo), Berlin, Germany

Note that full information on the approval of the study protocol must also be provided in the manuscript.

Human research participants

Policy information about studies involving human research participants

Population characteristics	Tissues biopsies obtained from female donors majorityly between 40-55-year age were used.
Recruitment	Upon receiving informed consent the biopsies were collected and samples from different donors were randomly assigned to experiments.
Ethics oversight	Human ecto- and endocervix samples were provided by the Department of Gynecology, Charite University Hospital, Berlin, Germany. Scientific usage of the samples was approved by the ethics committee of the Charite University Hospital, Berlin (EAI/059/15); informed consent to use their tissue for scientific research was obtained from all subjects.

Note that full information on the approval of the study protocol must also be provided in the manuscript.
