# Supplementary material for: Opposing Wnt signals regulate cervical squamocolumnar homeostasis and emergence of metaplasia
Source: Nat Cell Biol. 2021 Jan 18;23(2):184–97. doi: 10.1038/s41556-020-00619-0 (PMC7878191; doi:10.1038/s41556-020-00619-0)
Supplement: Supplementary file 2 — Peer review information [file 41556_2020_619_MOESM2_ESM.pdf]

naturerearch

Peer Review Information

Journal: Nature Cell Biology
Manuscript Title: Opposing Wnt signals regulate cervical squamocolumnar homeostasis and emergence of metaplasia
Corresponding author name(s): Thomas Meyer

Reviewer Comments & Decisions:

Decision Letter, initial version:

Date: 25th February 20 06:34:38
Last Sent: 25th February 20 06:34:38
Triggered By: Christine Weber
From: christine.weber@nature.com
To: meyer@mpiib-berlin.mpg.de
CC: edmund.irwin@nature.com
Subject: Decision on Nature Cell Biology submission NCB-M41689
Message: *Please delete the link to your author homepage if you wish to forward this email to co-authors.

Dear Dr. Meyer,

Your manuscript, "Stromal microenvironment controls homeostasis of the cervical squamo-columnar transition-zone and emergence of squamous metaplasia", has now been seen by 3 referees, who are experts in metaplasia, lineage tracing, stem cells (referee 1); cervical cancer (referee 2); and ovarian cancer, CSCs, stem cell niches (referee 3). As you will see from their comments (attached below) they find this work of potential interest, but have raised substantial concerns, which in our view would need to be addressed with considerable revisions before we can consider publication in Nature Cell Biology.

Nature Cell Biology editors discuss the referee reports in detail within the editorial team, including the chief editor, to identify key referee points that should be addressed with priority, and requests that are overruled as being beyond the scope of the current study. To guide the scope of the revisions, I have listed these points below. We are committed to providing a fair and constructive peer-review process, so please feel free to contact me if you would like to discuss any of the referee comments further.

In particular, it would be essential to address the following points:

(A) An improved characterization of the tissue and exact regions used to derive these findings should be provided, as highlighted by referees 1 and 3.

Referee 1 notes:

"1. Figure 1F-G: High-magnification inserts are needed to better appreciate the columnar and squamous phenotype.

Referee 3 notes:

"1. The delineation of endocervix, TZ and ectocervix used by authors is questionable. According to Extended Data Fig. 1, Fig. 5L and M, and Extended Data Fig. 3, the endocervix occupies around one third of the uterine horns. In Extended Data Fig. 5, areas shown as endocervix are located not far from the junction to oviduct! This cannot be accurate. Uterine horns, with an exception of a very small segment near the corpus, are lined by the endometrial epithelium supported by very distinct highly cellular endometrial stroma. Such endometrial stroma is present in many so-called "endocervical" images of this study. TZ seems to be placed too distal (too high in horn) to the actual transition zone. In some images (see e.g., Extended Data Fig. 3E), ectocervix in red box appears to be mostly vagina, although in the upper left corner of box it includes a bit of the ectocervix. Given this situation, it is unclear whether the current study accurately investigates endocervical biology. Were organoids also derived from the same generously defined "endocervix" regions?

2. Authors state that they combined cells from each region from 3 mice. Female reproductive tract undergoes significant changes during estrous cycle. At which stage mouse material has been collected?

3. Based on UMAP map of epithelial cells in Fig 1, it seems that the number of endocervical cells is smaller than that of TZ cells. Given that TZ is a very minute structure, this seems to be surprising. How did authors verify the accuracy of their material dissection?"

(B) Additional experimental proof for the role of Wnt signalling in the derivation of both lineages should be added and the characterization of the organoids should be extended, as noted by referees 1 and 3.

Referee 1 notes:

"2. The authors discussed that the presence of FSK increases organoid growth, however, Fig. 2C does not present statistical comparisons between +/-FSK samples within the same passage. Are there statistical differences between organoids exposed to FSK and those that did not (within the same passage)?

3. Do the endocervical organoids express Krt19 at the transcriptional and protein level (Ext. Data Fig. 2F-H)? This data should be presented given that the study emphases that columnar endocervical cells are characterized by Krt8hi/Krt19hi cells.

4. What was the characterization of squamous/columnar organoid based on? It's hard to believe that the population of organoids was -strictly- either columnar or squamous. A better method of quantification might be number of epithelial layers per organoid, or the distribution of number of p63+ cells per organoid.

5. Fig. 2A is used as reference for the statement on line 164-165 regarding the columnar phenotype of ectocervix organoids in the presence or absence of Wnt. These images do not clearly prove the statement. Higher magnification images are needed.

6. In various instances (such as lines 170) in the manuscript the authors describe DKK3 as a Wnt signaling inhibitor, and then in lines 199-200 described it as to either have no effect on Wnt signaling or to function as an agonist. These statements are contradictory.
The authors need to address why DKK3 supported columnar epithelia by restoring endocervical organoid size (Fig. 3E), and also is upregulated in squamous epithelium (Fig. 2G).

7. Does the addition of DKK2 in the media of endocervical organoids causes stratification? Is there a change in p63+ cell count? Is there any other phenotypical organoid change in addition to organoid size (Fig. 3E) that is comparable to the effect of medium devoid of Wnt3a/RSPO1?

8. The conclusions stated based on Extended Figure 5A-B need to be supported by quantitative methods, such as the # of p63+/KRT5+ cells in Wnt-deficient or proficient medium."

Referee 3 notes:

"4. Authors' conclusions about the opposing roles of WNT signals from the stroma are based on testing WNT growth factors in organoid assays and descriptive tissue phenotyping. These observations are important but offer only a circumstantial evidence. Firm conclusions would require testing of WNT signaling by its direct genetic interrogation (KO or KD of genes in question) in organoid systems and in mouse models."

(C) Lineage relationships and trajectories, as relevant in the derivation of the squamous and columnar epithelia, the role of the stroma in cell fate determination and the cells of origin of SCC, should be studied in more detail, as requested by referees 2 and 3.

Referee 2 notes:

"6. This study seems to suggest that the so called subglandular reserve cells do not exist. The findings that distinct stromal cells are detected in the endocervix, ectocervix and TZ, and that a unique stromal cluster was specifically found in metaplastic mice, seem to suggest that epithelial metaplasia is controlled by the surrounding stromal cells. This agrees with the signalling pathways required to maintain the stemness and differentiation state of the organoids. If this is indeed

the case and epithelial cell fate is determined by the agonists or/and antagonists secreted by stromal cells, then the authors should be able to demonstrate the causal effect of stromal cells using a co-culture system. One would expect that a co-culture of columnar endocervix epithelial organoids with the identified unique stromal cells derived from the ectocervix would convert columnar endocervix to squamous ectocervix and vice versa. Can this be experimentally tested and demonstrated?"

Referee 3 notes:

"5. The study does not present any evidence that squamous and columnar epithelia derive from lineage-specific stem cell populations in vivo. It only shows that squamous and columnar epithelia derive from 2 specific cell lineages. For the same reason, the statement about "outgrowth of quiescent ectocervical stem cells" is questionable.

6. Statement that "SCJ cells are not distinct from the endocervical columnar lineage and not the cells of origin of SCC" is speculative. Accurate in vivo modeling of such disease in required for such strong conclusion."

(D) A better distinction between the human and mouse situation should be provided, as pointed out by referees 1 and 2.

Referee 1 notes:

"9. The authors failed to explain why changes in the Wnt signaling microenvironment caused transdifferentiation of human epithelial organoids (Extended Fig, 5A) but was not observed in mouse organoids (Extended Fig, 5C). If the mouse cervix epithelia cannot recapitulate the mechanisms of transdifferentiation as the human, how is the mouse in vivo model suitable to model metaplasia?"

Referee 2 notes:

"The current manuscript does not distinguish whether certain findings are specific to the mouse tissues, whereas others are unique to human organoids. Many of the conclusions are presented as a generic finding.

1. Single cell RNA-seq of mouse ecto- and endocervix and transition zone identified four squamous, two columnar and one myoepithelial clusters. It was therefore concluded that squamous and columnar epithelium originate from keratin 5 and keratin 8 expressing cells, respectively. These data seem to suggest that the well-studied keratin 17 expressing subglandular reserve cells do not exist. It is important for the authors to explain why their data disagree with a number of studies in the literature. For example, is this a mouse specific phenomenon? It is also important for the authors to clarify the anatomical and structural similarities and differences between the human and mouse cervix, so that readers can have a good understanding of the single cell RNA-seq data from mouse cervical tissues.

2. Single cell RNA-seq of endo- and ectocervix of metaplastic mice showed the

expansion of squamous and myoepithelial cell clusters. What is the cell composition of the TZ in metaplastic mice? Can the authors detect keratin 17+ cells in the metaplastic endocervix? How about a keratin 8/p63/K5 expressing population, similar to that seen in human endocervix organoids grown in WNT-deficient medium (seen in figure 2)?

3. The authors observed that in the absence of WNT, human endocervix cells can give rise to p63+/K5+ stratified organoids, similar to those derived from ectocervix. Again, it would be good to clarify whether this is human specific. Can similar a phenomenon be seen in mouse endocervix organoids?

4. The single cell RNA-seq study revealed that distinctive stroma populations are detected in endocervix, ectocervix and TZ. A unique cluster of stromal cells is also identified in metaplastic mice. It will be important for the authors to investigate whether a similar phenomenon also exists in human endocervix, ectocervix and TZ using smRNA-FISH.

5. Mouse linage tracing experiments confirmed that keratin 5+ cells will give rise to squamous epithelium whereas keratin 8+ cells will generate columnar epithelium in vivo. Single molecule RNA-FISH also confirmed that keratin 5 and keratin 8 are detected in squamous and columnar epithelial cells in human and mouse cervix sections. These results are expected and not surprising. If only a few K5+/p63+/K8+ cells exist in the transition zone, is the linage tracing method sensitive enough to pick them up?”

(E) All other referee concerns pertaining to strengthening existing data, providing controls, methodological details, clarifications and textual changes should also be addressed.

(F) Finally please pay close attention to our guidelines on statistical and methodological reporting (listed below) as failure to do so may delay the reconsideration of the revised manuscript. In particular please provide:

- a Supplementary Figure including unprocessed images of all gels/blots in the form of a multi-page pdf file. Please ensure that blots/gels are labeled and the sections presented in the figures are clearly indicated.

- a Supplementary Table including all numerical source data in Excel format, with data for different figures provided as different sheets within a single Excel file. The file should include source data giving rise to graphical representations and statistical descriptions in the paper and for all instances where the figures present representative experiments of multiple independent repeats, the source data of all repeats should be provided.

We would be happy to consider a revised manuscript that would satisfactorily address these points, unless a similar paper is published elsewhere, or is accepted for publication in Nature Cell Biology in the meantime.

When revising the manuscript please:

- ensure that it conforms to our format instructions and publication policies (see below and www.nature.com/nature/authors/).

- provide a point-by-point rebuttal to the full referee reports verbatim, as provided at the end of this letter.

- provide the completed Editorial Policy Checklist (found here https://www.nature.com/authors/policies/Policy.pdf), and Reporting Summary (found here https://www.nature.com/authors/policies/ReportingSummary.pdf). This is essential for reconsideration of the manuscript and these documents will be available to editors and referees in the event of peer review. For more information see http://www.nature.com/authors/policies/availability.html or contact me.

Nature Cell Biology is committed to improving transparency in authorship. As part of our efforts in this direction, we are now requesting that all authors identified as 'corresponding author' on published papers create and link their Open Researcher and Contributor Identifier (ORCID) with their account on the Manuscript Tracking System (MTS), prior to acceptance. ORCID helps the scientific community achieve unambiguous attribution of all scholarly contributions. You can create and link your ORCID from the home page of the MTS by clicking on 'Modify my Springer Nature account'. For more information please visit please visit www.springernature.com/orcid.

Please submit the revised manuscript files and the point-by-point rebuttal to the referee comments using this link:

[REDACTED]

*This url links to your confidential home page and associated information about manuscripts you may have submitted or be reviewing for us. If you wish to forward this email to co-authors, please delete the link to your homepage.

We would like to receive a revised submission within six months. We would be happy to consider a revision even after this timeframe, however if the resubmission deadline is missed and the paper is eventually published, the submission date will be the date when the revised manuscript was received.

We hope that you will find our referees' comments, and editorial guidance helpful. Please do not hesitate to contact me if there is anything you would like to discuss.

Best wishes,

Christine.

Christine Weber, PhD
Senior Editor
Nature Cell Biology
E-mail: christine.weber@nature.com
Phone: +44 (0)207 843 4924

Reviewers' Comments:

Reviewer #1:
Remarks to the Author:
The authors utilized high-level transcriptional analyses, in vitro organoid technology and a lineage tracing using genetically-modified mice to characterize the drivers of cervical epithelial organization and the underlying stroma, specifically at the endo- and ectocervix as well as the transition zone. In addition, the authors elucidated the potential mechanism of squamous metaplasia driven by the endocervical stroma. This work is novel as it combines the power of transcriptional analyses, in vitro organoid culture and in vivo model to elucidate the independent contributions of distinct cervical epithelia and the underlying stroma at the cervical transition zone to the organization and metaplasia.

The manuscript could improve substantially given the following recommendations:
1. Figure 1F-G: High-magnification inserts are needed to better appreciate the columnar and squamous phenotype.
2. The authors discussed that the presence of FSK increases organoid growth, however, Fig. 2C does not present statistical comparisons between +/-FSK samples within the same passage. Are there statistical differences between organoids exposed to FSK and those that did not (within the same passage)?
3. Do the endocervical organoids express Krt19 at the transcriptional and protein level (Ext. Data Fig. 2F-H)? This data should be presented given that the study emphases that columnar endocervical cells are characterized by Krt8hi/Krt19hi cells.
4. What was the characterization of squamous/columnar organoid based on? It's hard to believe that the population of organoids was -strictly- either columnar or squamous. A better method of quantification might be number of epithelial layers per organoid, or the distribution of number of p63+ cells per organoid.
5. Fig. 2A is used as reference for the statement on line 164-165 regarding the columnar phenotype of ectocervix organoids in the presence or absence of Wnt. These images do not clearly prove the statement. Higher magnification images are needed.
6. In various instances (such as lines 170) in the manuscript the authors describe DKK3 as a Wnt signaling inhibitor, and then in lines 199-200 described it as to either have no effect on Wnt signaling or to function as an agonist. These statements are contradictory.
The authors need to address why DKK3 supported columnar epithelia by restoring endocervical organoid size (Fig. 3E), and also is upregulated in squamous epithelium (Fig. 2G).
7. Does the addition of DKK2 in the media of endocervical organoids causes stratification? Is there a change in p63+ cell count? Is there any other phenotypical organoid change in addition to organoid size (Fig. 3E) that is comparable to the effect of medium devoid of Wnt3a/RSPO1?
8. The conclusions stated based on Extended Figure 5A-B need to be supported by quantitative methods, such as the # of p63+/KRT5+ cells in Wnt-deficient or proficient medium.
9. The authors failed to explain why changes in the Wnt signaling

microenvironment caused transdifferentiation of human epithelial organoids (Extended Fig, 5A) but was not observed in mouse organoids (Extended Fig, 5C). If the mouse cervix epithelia cannot recapitulate the mechanisms of transdifferentiation as the human, how is the mouse in vivo model suitable to model metaplasia?

Minor edits:
1. There are multiple error bars that need to be fixed, either because labeling is missing or because the authors used "uM" as opposed to "um". Example: scale bar on Fig 1H-I.

Reviewer #2:
Remarks to the Author:
Most cervical cancers arise at transition or transformation zone (TZ), which is located at the squamous and columnar junction (SCJ) of the cervix, and are characterised by the presence of epithelial metaplasia, an expansion or inward growth of squamous epithelial cells to replace columnar epithelial cells. This leads to the hypothesis that epithelial metaplasia may originate from a special progenitor cell population in the TZ. A number of studies have suggested that keratin 17-expressing subglandular reserve cells could be one such progenitor population. In addition to cervical cancers, many other human cancers and pathological conditions - such as esophageal adenocarcinoma and Barrett's esophagus - also occur at a TZ, with features of columnar epithelial cells outgrowing squamous epithelium. These examples illustrate the importance of identifying the unique cell population, the signalling pathways, and the microenviromental niche that enable and facilitate the occurrence of metaplasia.

In this manuscript, the authors used a combination of state of the art technologies (single cell RNA-seq, organoid culture and mouse linage tracing) to study key signalling pathways and cell types that control the homeostasis of the cervical squamo-columnar junction and the potential causes of squamous metaplasia of the cervix. The conclusions from this study were derived from analysing combined RNA expression data derived from single cell RNA sequencing of control healthy mouse endocervix, ectocervix, transition zone, and endocervix from mice with metaplasia, and microarray analysis of human squamous ectocervical and columnar endocervical organoids. Mouse linage tracing and smRNA-FISH confirmed the finding that keratin 5 or keratin 8 expressing cells give rise to squamous or columnar epithelium in vivo, as expected, and it was concluded that human cervical adenocarcinoma and squamous carcinomas were derived from columnar and squamous epithelial linages respectively.

This is a comprehensive analysis of mouse endo- and ectocervix from control and metaplastic mice using single cell RNA-seqencing analysis and many of the findings are clear and convincing. The linage tracing and smRNA-FISH analyses provide further evidence supporting the findings derived from single cell RNA-seq analysis. Additionally, this manuscript used human ectocervix and endocervix organoids for various analyses.

Although a number of potentially interesting findings have been made, the importance of the claims need further clarification. This is largely due to the fact that some data are derived from human organoids whereas others were derived from single cell RNA seq of mouse tissues. The current manuscript does not distinguish whether certain findings are specific to the mouse tissues, whereas others are unique to human organoids. Many of the conclusions are presented as a generic findings.

1) Single cell RNA-seq of mouse ecto- and endocervix and transition zone identified four squamous, two columnar and one myoepithelial clusters. It was therefore concluded that squamous and columnar epithelium originate from keratin 5 and keratin 8 expressing cells, respectively. These data seem to suggest that the well-studied keratin 17 expressing subglandular reserve cells do not exist. It is important for the authors to explain why their data disagree with a number of studies in the literature. For example, is this a mouse specific phenomenon? It is also important for the authors to clarify the anatomical and structural similarities and differences between the human and mouse cervix, so that readers can have a good understanding of the single cell RNA-seq data from mouse cervical tissues.

2) Single cell RNA-seq of endo- and ectocervix of metaplastic mice showed the expansion of squamous and myoepithelial cell clusters. What is the cell composition of the TZ in metaplastic mice? Can the authors detect keratin 17+ cells in the metaplastic endocervix? How about a keratin 8/p63/K5 expressing population, similar to that seen in human endocervix organoids grown in WNT-deficient medium (seen in figure 2)?

3) The authors observed that in the absence of WNT, human endocervix cells can give rise to p63+/K5+ stratified organoids, similar to those derived from ectocervix. Again, it would be good to clarify whether this is human specific. Can similar a phenomenon be seen in mouse endocervix organoids?

4) The single cell RNA-seq study revealed that distinctive stroma populations are detected in endocervix, ectocervix and TZ. A unique cluster of stromal cells is also identified in metaplastic mice. It will be important for the authors to investigate whether a similar phenomenon also exists in human endocervix, ectocervix and TZ using smRNA-FISH.

5) Mouse linage tracing experiments confirmed that keratin 5+ cells will give rise to squamous epithelium whereas keratin 8+ cells will generate columnar epithelium in vivo. Single molecule RNA-FISH also confirmed that keratin 5 and keratin 8 are detected in squamous and columnar epithelial cells in human and mouse cervix sections. These results are expected and not surprising. If only a few K5+/p63+/K8+ cells exist in the transition zone, is the linage tracing method sensitive enough to pick them up?

6) This study seems to suggest that the so called subglandular reserve cells do not exist. The findings that distinct stromal cells are detected in the endocervix, ectocervix and TZ, and that a unique stromal cluster was specifically found in metaplastic mice, seem to suggest that epithelial metaplasia is controlled by the surrounding stromal cells. This agrees with the signalling pathways required to maintain the stemness and differentiation state of the organoids. If this is indeed

the case and epithelial cell fate is determined by the agonists or/and antagonists secreted by stromal cells, then the authors should be able to demonstrate the causal effect of stromal cells using a co-culture system. One would expect that a co-culture of columnar endocervix epithelial organoids with the identified unique stromal cells derived from the ectocervix would convert columnar endocervix to squamous ectocervix and vice versa. Can this be experimentally tested and demonstrated?

Minor comments

1) The authors should state the numbers of single cells sequenced and analysed in each figure.

2) A more comprehensive overview of the field will be needed for the introduction. The authors also should discuss reasons for the discrepancies between their findings and previously published work.

Reviewer #3:
Remarks to the Author:
The manuscript Chumduri et al attempts to describe how stromal microenvironment controls homeostasis of the cervical squamo-columnar zone (aka or squamo-columnar junction or transition zone, TZ). It also touches on potential factors regulating the emergence of squamous metaplasia induced by vitamin A deficiency. The topics addressed in this study are important and reported finding may provide some additional information about interactions between squamous and columnar epithelia at the area of their junction. The finding that squamous and columnar epithelia derive from two distinct cell lineages is not entirely surprising. However, authors provide convincing lineage tracing results supporting this conclusion. Unfortunately, there is a number of serious concerns about the study in its present form.

1) The delineation of endocervix, TZ and ectocervix used by authors is questionable. According to Extended Data Fig. 1, Fig. 5L and M, and Extended Data Fig. 3, the endocervix occupies around one third of the uterine horns. In Extended Data Fig. 5, areas shown as endocervix are located not far from the junction to oviduct! This cannot be accurate. Uterine horns, with an exception of a very small segment near the corpus, are lined by the endometrial epithelium supported by very distinct highly cellular endometrial stroma. Such endometrial stroma is present in many so-called "endocervical" images of this study. TZ seems to be placed too distal (too high in horn) to the actual transition zone. In some images (see e.g., Extended Data Fig. 3E), ectocervix in red box appears to be mostly vagina, although in the upper left corner of box it includes a bit of the ectocervix. Given this situation, it is unclear whether the current study accurately investigates endocervical biology. Were organoids also derived from the same generously defined "endocervix" regions?

2) Authors state that they combined cells from each region from 3 mice. Female reproductive tract undergoes significant changes during estrous cycle. At which stage mouse material has been collected?

3) Based on UMAP map of epithelial cells in Fig 1, it seems that the number of endocervical cells is smaller than that of TZ cells. Given that TZ is a very minute structure, this seems to be surprising. How did authors verify the accuracy of their material dissection?

4) Authors' conclusions about the opposing roles of WNT signals from the stroma are based on testing WNT growth factors in organoid assays and descriptive tissue phenotyping. These observations are important but offer only a circumstantial evidence. Firm conclusions would require testing of WNT signaling by its direct genetic interrogation (KO or KD of genes in question) in organoid systems and in mouse models.

5) The study does not present any evidence that squamous and columnar epithelia derive from lineage-specific stem cell populations in vivo. It only shows that squamous and columnar epithelia derive from 2 specific cell lineages. For the same reason, the statement about "outgrowth of quiescent ectocervical stem cells" is questionable.

6) Statement that "SCJ cells are not distinct from the endocervical columnar lineage and not the cells of origin of SCC" is speculative. Accurate in vivo modeling of such disease in required for such strong conclusion.

Minor issues:

1) Has single cell transcriptome experiment been done on a single sample of 3 pooled mice or was it repeated 3 times?

2) How many cells were loaded on 10X Chromium controller?

3) Generation of cell lineage trajectories by Monocle or some similar program could strengthen this study.

GUIDELINES FOR MANUSCRIPT SUBMISSION TO NATURE CELL BIOLOGY

READABILITY OF MANUSCRIPTS – Nature Cell Biology is read by cell biologists from diverse backgrounds, many of whom are not native English speakers. Authors should aim to communicate their findings clearly, explaining technical jargon that might be unfamiliar to non-specialists, and avoiding non-standard abbreviations. Titles and abstracts should concisely communicate the main findings of the study, and the background, rationale, results and conclusions should be clearly explained in the manuscript in a manner accessible to a broad cell biology audience. Nature Cell Biology uses British spelling.

MANUSCRIPT FORMAT – please follow the guidelines listed in our Guide to Authors regarding manuscript formats at Nature Cell Biology.

TITLE – should be no more than 100 characters including spaces, without punctuation and avoiding technical terms, abbreviations, and active verbs..

AUTHOR NAMES – should be given in full.

AUTHOR AFFILIATIONS – should be denoted with numerical superscripts (not symbols) preceding the names. Full addresses should be included, with US states in full and providing zip/post codes. The corresponding author is denoted by: "Correspondence should be addressed to [initials]."

ABSTRACT AND MAIN TEXT – please follow the guidelines that are specific to the format of your manuscript, as listed in our Guide to Authors (http://www.nature.com/ncb/pdf/ncb_gta.pdf) Briefly, Nature Cell Biology Articles, Resources and Technical Reports have 3500 words, including a 150 word abstract, and the main text is subdivided in Introduction, Results, and Discussion sections. Nature Cell Biology Letters have up to 2500 words, including a 180 word introductory paragraph (abstract), and the text is not subdivided in sections.

ACKNOWLEDGEMENTS – should be kept brief. Professional titles and affiliations are unnecessary. Grant numbers can be listed.

AUTHOR CONTRIBUTIONS – must be included after the Acknowledgements, detailing the contributions of each author to the paper (e.g. experimental work, project planning, data analysis etc.). Each author should be listed by his/her initials.

FINANCIAL AND NON-FINANCIAL COMPETING INTERESTS – the authors must include one of three declarations: (1) that they have no financial and non-financial competing interests; (2) that they have financial and non-financial competing interests; or (3) that they decline to respond, after the Author Contributions section. This statement will be published with the article, and in cases where financial and non-financial competing interests are declared, these will be itemized in a web supplement to the article. For further details please see https://www.nature.com/licenceforms/nrg/competing-interests.pdf.

REFERENCES – are limited to a total of 70 for Articles, Resources, Technical Reports; and 40 for Letters. This includes references in the main text and Methods combined. References must be numbered sequentially as they appear in the main text, tables and figure legends and Methods and must follow the precise style of Nature Cell Biology references. References only cited in the Methods should be numbered consecutively following the last reference cited in the main text. References only associated with Supplementary Information (e.g. in supplementary legends) do not count toward the total reference limit and do not need to be cited in numerical continuity with references in the main text. Only published papers can be cited, and each publication cited should be included in the numbered reference list, which should include the manuscript titles. Footnotes are not permitted.

METHODS – Nature Cell Biology publishes methods online. The methods section should be provided as a separate Word document, which will be copyedited and appended to the manuscript PDF, and incorporated within the HTML format of the paper.

Methods should be written concisely, but should contain all elements necessary to allow interpretation and replication of the results. As a guideline, Methods sections typically do not exceed 3,000 words. The Methods should be divided into subsections listing reagents and techniques. When citing previous methods, accurate references should be provided and any alterations should be noted. Information must be provided about: antibody dilutions, company names, catalogue numbers and clone numbers for monoclonal antibodies; sequences of RNAi and cDNA probes/primers or company names and catalogue numbers if reagents are commercial; cell line names, sources and information on cell line identity and authentication. Animal studies and experiments involving human subjects must be reported in detail, identifying the committees approving the protocols. For studies involving human subjects/samples, a statement must be included confirming that informed consent was obtained. Statistical analyses and information on the reproducibility of experimental results should be provided in a section titled "Statistics and Reproducibility".

All Nature Cell Biology manuscripts submitted on or after March 21 2016 must include a Data availability statement at the end of the Methods section. For Springer Nature policies on data availability see http://www.nature.com/authors/policies/availability.html; for more information on this particular policy see http://www.nature.com/authors/policies/data/data-availability-statements-data-citations.pdf. The Data availability statement should include:

• Accession codes for primary datasets (generated during the study under consideration and designated as "primary accessions") and secondary datasets (published datasets reanalysed during the study under consideration, designated as "referenced accessions"). For primary accessions data should be made public to coincide with publication of the manuscript. A list of data types for which submission to community-endorsed public repositories is mandated (including sequence, structure, microarray, deep sequencing data) can be found here http://www.nature.com/authors/policies/availability.html#data.

• Unique identifiers (accession codes, DOIs or other unique persistent identifier) and hyperlinks for datasets deposited in an approved repository, but for which data deposition is not mandated (see here for details http://www.nature.com/sdata/data-policies/repositories).

• At a minimum, please include a statement confirming that all relevant data are available from the authors, and/or are included with the manuscript (e.g. as source data or supplementary information), listing which data are included (e.g. by figure panels and data types) and mentioning any restrictions on availability.

• If a dataset has a Digital Object Identifier (DOI) as its unique identifier, we strongly encourage including this in the Reference list and citing the dataset in the

Methods.

We recommend that you upload the step-by-step protocols used in this manuscript to the Protocol Exchange. More details can found at www.nature.com/protocolexchange/about.

DISPLAY ITEMS – main display items are limited to 6-8 main figures and/or main tables for Articles, Resources, Technical Reports; and 5 main figures and/or main tables for Letters. For Supplementary Information see below.

FIGURES – Colour figure publication costs $600 for the first, and $300 for each subsequent colour figure. All panels of a multi-panel figure must be logically connected and arranged as they would appear in the final version. Unnecessary figures and figure panels should be avoided (e.g. data presented in small tables could be stated briefly in the text instead).

All imaging data should be accompanied by scale bars, which should be defined in the legend.
Cropped images of gels/blots are acceptable, but need to be accompanied by size markers, and to retain visible background signal within the linear range (i.e. should not be saturated). The boundaries of panels with low background have to be demarked with black lines. Splicing of panels should only be considered if unavoidable, and must be clearly marked on the figure, and noted in the legend with a statement on whether the samples were obtained and processed simultaneously. Quantitative comparisons between samples on different gels/blots are discouraged; if this is unavoidable, it should only be performed for samples derived from the same experiment with gels/blots were processed in parallel, which needs to be stated in the legend.

Figures should be provided at approximately the size that they are to be printed at (single column is 86 mm, double column is 170 mm) and should not exceed an A4 page (8.5 x 11"). Reduction to the scale that will be used on the page is not necessary, but multi-panel figures should be sized so that the whole figure can be reduced by the same amount at the smallest size at which essential details in each panel are visible. In the interest of our colour-blind readers we ask that you avoid using red and green for contrast in figures. Replacing red with magenta and green with turquoise are two possible colour-safe alternatives. Lines with widths of less than 1 point should be avoided. Sans serif typefaces, such as Helvetica (preferred) or Arial should be used. All text that forms part of a figure should be rewritable and removable.

We accept files from the following graphics packages in either PC or Macintosh format:

- For line art, graphs, charts and schematics we prefer Adobe Illustrator (.AI), Encapsulated PostScript (.EPS) or Portable Document Format (.PDF). Files should be saved or exported as such directly from the application in which they were made, to allow us to restyle them according to our journal house style.

- We accept PowerPoint (.PPT) files if they are fully editable. However, please

refrain from adding PowerPoint graphical effects to objects, as this results in them outputting poor quality raster art. Text used for PowerPoint figures should be Helvetica (preferred) or Arial.

- We do not recommend using Adobe Photoshop for designing figures, but we can accept Photoshop generated (.PSD or .TIFF) files only if each element included in the figure (text, labels, pictures, graphs, arrows and scale bars) are on separate layers. All text should be editable in 'type layers' and line-art such as graphs and other simple schematics should be preserved and embedded within 'vector smart objects' - not flattened raster/bitmap graphics.

- Some programs can generate Postscript by 'printing to file' (found in the Print dialogue). If using an application not listed above, save the file in PostScript format or email our Art Editor, Allen Beattie for advice (a.beattie@nature.com).

Regardless of format, all figures must be vector graphic compatible files, not supplied in a flattened raster/bitmap graphics format, but should be fully editable, allowing us to highlight/copy/paste all text and move individual parts of the figures (i.e. arrows, lines, x and y axes, graphs, tick marks, scale bars etc.). The only parts of the figure that should be in pixel raster/bitmap format are photographic images or 3D rendered graphics/complex technical illustrations.

All placed images (i.e. a photo incorporated into a figure) should be on a separate layer and independent from any superimposed scale bars or text. Individual photographic images must be a minimum of 300+ DPI (at actual size) or kept constant from the original picture acquisition and not decreased in resolution post image acquisition. All colour artwork should be RGB format.

FIGURE LEGENDS – must not exceed 350 words for each figure to allow fit on a single printed NCB page together with the figure. They must include a brief title for the whole figure, and short descriptions of each panel with definitions of the symbols used, but without detailing methodology.

TABLES – main tables should be provided as individual Word files, together with a brief title and legend. For supplementary tables see below.

SUPPLEMENTARY INFORMATION – Supplementary information is material directly relevant to the conclusion of a paper, but which cannot be included in the printed version in order to keep the manuscript concise and accessible to the general reader. Supplementary information is an integral part of a Nature Cell Biology publication and should be prepared and presented with as much care as the main display item, but it must not include non-essential data or text, which may be removed at the editor's discretion. All supplementary material is fully peer-reviewed and published online as part of the HTML version of the manuscript. Supplementary Figures and Supplementary Notes are appended at the end of the main PDF of the published manuscript.

Supplementary items should relate to a main text figure, wherever possible, and should be mentioned sequentially in the main manuscript, designated as

Supplementary Figure, Table, Video, or Note, and numbered continuously (e.g. Supplementary Figure 1, Supplementary Figure 2, Supplementary Table 1, Supplementary Table 2 etc.).

Unprocessed scans of all key data generated through electrophoretic separation techniques need to be presented in a supplementary figure that should be labelled and numbered as the final supplementary figure, and should be mentioned in every relevant figure legend. This figure does not count towards the total number of figures and is the only figure that can be displayed over multiple pages, but should be provided as a single file, in PDF or TIFF format. Data in this figure can be displayed in a relatively informal style, but size markers and the figures panels corresponding to the presented data must be indicated.

The total number of Supplementary Figures (not including the "unprocessed scans" Supplementary Figure) should not exceed the number of main display items (figures and/or tables (see our Guide to Authors and March 2012 editorial http://www.nature.com/ncb/authors/submit/index.html#suppinfo; http://www.nature.com/ncb/journal/v14/n3/index.html#ed). No restrictions apply to Supplementary Tables or Videos, but we advise authors to be selective in including supplemental data.

Each Supplementary Figure should be provided as a single page and as an individual file in one of our accepted figure formats and should be presented according to our figure guidelines (see above). Supplementary Tables should be provided as individual Excel files. Supplementary Videos should be provided as .avi or .mov files up to 50 MB in size. Supplementary Figures, Tables and Videos much be accompanied by a separate Word document including titles and legends.

GUIDELINES FOR EXPERIMENTAL AND STATISTICAL REPORTING

REPORTING REQUIREMENTS – To improve the quality of methods and statistics reporting in our papers we have recently revised the reporting checklist we introduced in 2013. We are now asking all life sciences authors to complete two items: an Editorial Policy Checklist (found here https://www.nature.com/authors/policies/Policy.pdf) that verifies compliance with all required editorial policies and a reporting summary (found here https://www.nature.com/authors/policies/ReportingSummary.pdf) that collects information on experimental design and reagents. These documents are available to referees to aid the evaluation of the manuscript. Please note that these forms are dynamic 'smart pdfs' and must therefore be downloaded and completed in Adobe Reader. We will then flatten them for ease of use by the reviewers. If you would like to reference the guidance text as you complete the template, please access these flattened versions at http://www.nature.com/authors/policies/availability.html.

STATISTICS – Wherever statistics have been derived the legend needs to provide the n number (i.e. the sample size used to derive statistics) as a precise value (not a range), and define what this value represents. Error bars need to be defined in the legends (e.g. SD, SEM) together with a measure of centre (e.g. mean, median). Box plots need to be defined in terms of minima, maxima, centre, and

percentiles. Ranges are more appropriate than standard errors for small data sets. Wherever statistical significance has been derived, precise p values need to be provided and the statistical test used needs to be stated in the legend. Statistics such as error bars must not be derived from n<3. For sample sizes of n<5 please plot the individual data points rather than providing bar graphs. Deriving statistics from technical replicate samples, rather than biological replicates is strongly discouraged. Wherever statistical significance has been derived, precise p values need to be provided and the statistical test stated in the legend.

Information on how many times each experiment was repeated independently with similar results needs to be provided in the legends and/or Methods for all experiments, and in particular wherever representative experiments are shown.

We strongly recommend the presentation of source data for graphical and statistical analyses as a separate Supplementary Table, and request that source data for all independent repeats are provided when representative experiments of multiple independent repeats, or averages of two independent experiments are presented. This supplementary table should be in Excel format, with data for different figures provided as different sheets within a single Excel file. It should be labelled and numbered as one of the supplementary tables, titled "Statistics Source Data", and mentioned in all relevant figure legends.

Author Rebuttal to Initial comments

Point by point response to the Reviewers' comments

Reviewers' Comments:

Reviewer #1:

Remarks to the Author:

The authors utilized high-level transcriptional analyses, in vitro organoid technology and a lineage tracing using genetically-modified mice to characterize the drivers of cervical epithelial organization and the underlying stroma, specifically at the endo- and ectocervix as well as the transition zone. In addition, the authors elucidated the potential mechanism of squamous metaplasia driven by the endocervical stroma. This work is novel as it combines the power of transcriptional analyses, in vitro organoid culture and in vivo model to elucidate the independent contributions of distinct cervical epithelia and the underlying stroma at the cervical transition zone to the organization and metaplasia.

We appreciate the reviewer's opinion on the novelty and importance of this work.

The manuscript could improve substantially given the following recommendations:
1. Figure 1F-G: High-magnification inserts are needed to better appreciate the columnar and squamous phenotype.

Higher magnification insets of the TZ, where columnar and squamous epithelia meet, were provided in the previous version of the Manuscript in Figure 6E-F and Extended Data Fig. 1I-J. In the revised version, better high-magnification insets are shown in Figure 1G-H in addition to Figure 6E-F.

2. The authors discussed that the presence of FSK increases organoid growth, however, Fig. 2C does not present statistical comparisons between +/-FSK samples within the same passage. Are there statistical differences between organoids exposed to FSK and those that did not (within the same passage)?

We now included the statistical significance in Figure 2C. The data show statistically significant difference in organoid size between -FSK and +FSK within the same passage.

3. Do the endocervical organoids express Krt19 at the transcriptional and protein level (Ext. Data Fig. 2F-H)? This data should be presented given that the study emphases that columnar endocervical cells are characterized by Krt8hi/Krt19hi cells.

Now we have included the immune-stained images of KRT19 protein in human ectocervical and endocervical organoids (Figure 2I). In agreement with the mouse single-cell data (Ext. Data Fig. 1H), the additional data clearly confirm that columnar organoids express KRT19.

4. What was the characterization of squamous/columnar organoid based on? It's hard to believe that the population of organoids was -strictly- either columnar or squamous. A better method of quantification might be number of epithelial layers per organoid, or the distribution of number of p63+ cells per organoid.

As explained in the text (lines 143-154), columnar organoids are distinct from squamous organoids. Under bright field microscope columnar monolayer organoids are hollow and significantly larger, while squamous stratified organoids are dense/dark due to the strictly-consistent multilayered assembly of cells. This contrast-based classification was repeatedly confirmed by many IHC stainings, including for p63, KRT5, KRT7, KRT8, KRT19 and E-Cadherin as shown in Figure 2D and E, Figure 2H-J, Ext. Data Fig. 2H-I, Figure 3H, Ext. Data Figure 5D, Figure 6G and Ext. Data Fig. 6G-I to support our conclusions.

5. Fig. 2A is used as reference for the statement on line 164-165 regarding the columnar phenotype of ectocervix organoids in the presence or absence of Wnt. These images do not clearly prove the statement. Higher magnification images are needed.

Now, we provide the higher magnification images of the organoids as insets. Ectocervical cells always produce stratified epithelium. This is shown in bright field images in Figure 2A. Higher magnification with immunostaining for specific markers were shown in Figure 2E and Ext. Data Fig. 2H in the original Manuscript and now in Ext. Data Fig. 2I. Further, we have also shown that ectocervical organoids maintain the squamous stratified phenotype irrespective of WNT-positive microenvironment, previously in Ext. Data Fig. 5C and now in Ext. Data Fig. 5D.

6. In various instances (such as lines 170) in the manuscript the authors describe DKK3 as a Wnt signaling inhibitor, and then in lines 199-200 described it as to either have no effect on Wnt signaling or to function as an agonist. These statements are contradictory. The authors need to address why DKK3 supported columnar epithelia by restoring endocervical organoid size (Fig. 3E), and also is upregulated in squamous epithelium (Fig. 2G).

We find your remark correct, and in the current version of the MS, we refer to DKK3 as a WNT regulator. The data in Figure 3E show that organoid size is significantly reduced in the presence of DKK2, comparable to medium without WNT3a or RSPO1. This reduction in size was prevented when the medium additionally contained DKK3. In the absence of DKK2, DKK3 further increased organoid size. These results confirm that DKK2 and DKK3 do in fact exhibit opposing effects on the growth of columnar epithelium, with DKK3 promoting growth similar to WNT agonists. In Figure 4G, we change the term “WNT inhibitors” to “WNT regulators”.

Interestingly, while DKK1, -2, and -4 are structurally closely related, DKK3 is more distinct (Niehrs C et al., 2006, PMID: 17143291; Glinka et al., 1998, PMID: 9450748. The former three bind to LRP5/6 with high affinity, inducing endocytosis to inhibit WNT signaling (Bafico et al., 2001, PMID: 11433302; Mao et al., 2001, PMID: 11357136; Semenov et al., 2001, PMID: 11448771. In contrast, DKK3 does not bind LRP6 and does not influence WNT signaling via LRP5/6 route (Mao et al., 2001, PMID: 11357136; Mao and Niehrs et al., 2003, PMID: 12527209; Nakamura RE et al., 2010, PMID: 20370576), but rather regulates TGF-β signaling (Pinho and Niehrset al., 2007, PMID: 17490412). Further, Rei et al., 2010, PMID: 20370576 propose that DKK3 forms complexes with KRM proteins, possibly preventing their surface expression, which would provide a more favorable environment for WNT signaling.

7. Does the addition of DKK2 in the media of endocervical organoids causes stratification? Is there a change in p63+ cell count? Is there any other phenotypical organoid change in addition to organoid size (Fig. 3E) that is comparable to the effect of medium devoid of Wnt3a/RSPO1?

According to our observations, DKK2 evidently induces neither stratification nor p63 expression in medium of endocervical organoids. We have now included

immunostained images of KRT5/p63/KRT8 in Figure 3H. We see that treatment of DKK2 reduced the organoid size similar to endocervical organoids in WNT3/RSPO1-deficient medium. No other visual phenotypic changes were observed.

8. The conclusions stated based on Extended Figure 5A-B need to be supported by quantitative methods, such as the # of p63+/KRT5+ cells in Wnt-deficient or proficient medium.

We have now included the quantitative data in Ext. Data Fig. 5B. In Ext. Data Fig. 5C (previously 5B), all the cells are p63 positive.

9. The authors failed to explain why changes in the Wnt signaling microenvironment caused transdifferentiation of human epithelial organoids (Extended Fig, 5A) but was not observed in mouse organoids (Extended Fig, 5C). If the mouse cervix epithelia cannot recapitulate the mechanisms of transdifferentiation as the human, how is the mouse in vivo model suitable to model metaplasia?

Here, we would like to clarify that identical mechanisms of WNT-regulated lineage specification are true for both mouse and human epithelial cells. Our work, in fact, excludes transdifferentiation of the columnar lineage to the stratified squamous lineage or vice versa. Irrespective of their human or mouse origin, when cells are isolated from endocervix and cultured in WNT-deficient media, they grow in vitro as squamous stratified organoids (Figure 2A and Ext. Data Fig. 2G). In contrast, presence of a WNT microenvironment or supplemented WNT growth factors support the growth of columnar organoids. These results do not represent transdifferentiation, but rather proliferation of the squamous lineage under WNT-deficient conditions and WNT-dependent proliferation of the columnar lineage. Columnar cells cannot transdifferentiate into stratified organoids under WNT-deficient conditions and squamous cells cannot transdifferentiate into columnar organoids under WNT-proficient conditions (Figure 3H, Figure 5A and Ext. Data Fig. 5D). This is further supported by isolation of KRT8-labelled cells that can only form KRT8+ columnar organoids regardless of WNT conditions, while isolation of KRT5-labelled cells resulted in the formation of squamous organoids only at WNT-deficient media (Figure 5A).

It should be emphasized that stem cells residing in the endocervix can give rise to either columnar organoids or stratified organoids depending on existing or absence of WNT signals, respectively (Figure 2E-F and Ext. Data Fig. 2H-J). On the contrary, stem cells residing in the ectocervix predominantly give rise to squamous stratified organoids. In the absence of WNT signals, they grow well, whereas in the presence of WNT signals, they don't; however, under both conditions they maintain similar phenotype (Figure 2C and Ext. Data 2A).

The 2D primary culture of endocervical cells involves maintenance of stem cells that can later form either columnar or squamous organoids. Yet, there is enrichment of KRT7+ cells under WNT-proficient conditions and enrichment of KRT5+ cells under WNT-deficient conditions. On the contrary, 2D primary culture of the ectocervix has no stem cells that can differentiate into columnar organoids, only into squamous organoids (Ext. Data Fig. 5C).

Together, our results indicate the existence of two distinct types of epithelial lineage specific stem cells in the endocervical tissue microenvironment which respond differentially to the Wnt microenvironment and rule out epithelial transdifferentiation involvement as a mechanism governing cervical metaplasia.

Minor edits:

1. There are multiple error bars that need to be fixed, either because labeling is missing or because the authors used “uM” as opposed to “um”. Example: scale bar on Fig 1H-I.

Now we have updated the labeling with μm.

Reviewer #2:

Remarks to the Author:

Most cervical cancers arise at transition or transformation zone (TZ), which is located at the squamous and columnar junction (SCJ) of the cervix, and are characterised by the presence of epithelial metaplasia, an expansion or inward growth of squamous epithelial cells to replace columnar epithelial cells. This leads to the hypothesis that epithelial metaplasia may originate from a special progenitor cell population in the TZ. A number of studies have suggested that keratin 17-expressing subglandular reserve cells could be one such progenitor population. In addition to cervical cancers, many other human cancers and pathological conditions - such as esophageal adenocarcinoma and Barrett’s esophagus - also occur at a TZ, with features of columnar epithelial cells outgrowing squamous epithelium. These examples illustrate the importance of identifying the unique cell population, the signalling pathways, and the microenviromental niche that enable and facilitate the occurrenceof metaplasia.

In this manuscript, the authors used a combination of state of the art technologies (single cell RNA-seq, organoid culture and mouse linage tracing) to study key signalling pathways and cell types that control the homeostasis of the cervical squamo-columnar junction and the potential causes of squamous metaplasia of the cervix. The conclusions from this study were derived from analysing combined RNA expression data derived from single cell RNA sequencing of control healthy mouse endocervix, ectocervix, transition zone, and endocervix from mice with metaplasia, and microarray analysis of human squamous ectocervical and columnar endocervical organoids. Mouse linage tracing and smRNA-FISH confirmed the finding that keratin 5 or keratin 8 expressing cells give rise to squamous or columnar epithelium in vivo, as

expected, and it was concluded that human cervical adenocarcinoma and squamous carcinomas were derived from columnar and squamous epithelial linages respectively.

This is a comprehensive analysis of mouse endo- and ectocervix from control and metaplastic mice using single cell RNA-seqencing analysis and many of the findings are clear and convincing. The linage tracing and smRNA-FISH analyses provide further evidence supporting the findings derived from single cell RNA-seq analysis. Additionally, this manuscript used human ectocervix and endocervix organoids for various analyses.

Although a number of potentially interesting findings have been made, the importance of the claims need further clarification. This is largely due to the fact that some data are derived from human organoids whereas others were derived from single cell RNA seq of mouse tissues. The current manuscript does not distinguish whether certain findings are specific to the mouse tissues, whereas others are unique to human organoids. Many of the conclusions are presented as a generic findings.

1) Single cell RNA-seq of mouse ecto- and endocervix and transition zone identified four squamous, two columnar and one myoepithelial clusters. It was therefore concluded that squamous and columnar epithelium originate from keratin 5 and keratin 8 expressing cells, respectively. These data seem to suggest that the well-studied keratin 17 expressing subglandular reserve cells do not exist. It is important for the authors to explain why their data disagree with a number of studies in the literature. For example, is this a mouse specific phenomenon? It is also important for the authors to clarify the anatomical and structural similarities and differences between the human and mouse cervix, so that readers can have a good understanding of the single cell RNA-seq data from mouse cervical tissues.

In line with the reviewer's notion, we would like to emphasize that distinct progenitor cells (reserve cells) within the endocervical tissue are the ones that give rise to lineage specific squamous metaplasia, rather than the transdifferentiation of columnar KRT8+ epithelial cells.

As suggested in literature, KRT17+ subcolumnar reserve cells also express KRT5 (Martens JE et al, 2009, PMID: 19483623). In corroboration to this, our study identified KRT17 to be highly expressed in ectocervical organoids, which are rich in squamous cells, rather than in endocervical organoids, which are rich in columnar cells (Ext. Data Fig. 2F). Additionally, we have now performed immunostaining for KRT17 in human organoids (Figure 2J), human tissue (Ext. Data Fig. 5J-K) as well as in healthy and metaplastic mouse tissue (Ext. Data Fig. 5M, O and Q.) These data reveal the existence/emergence of KRT17/p63/Krt5 positive subcolumnar reserve cells in both human cervix and mouse metaplasia. No differences can be identified in terms of KRT17 as a marker for subcolumnar reserve cells between mouse and human.

Regarding the identification of sub-columnar KRT5/KRT17 cell in mice, we rarely found such population in mice except for the TZ or SCJ (Ext. Data Fig. 1J and Ext. Data Fig. 5 M, O and Q). In human specimens, we observed variable numbers of these sub-columnar cells (reserve cells) in the TZ and in endocervix amongst the different donors with highest incidence at the TZ. Yet, one should consider a larger TZ in humans as compared to mice. This observation of the distribution of these sub-columnar cells is in

line with previous studies (Martens JE et al, 2009, PMID: 19483623). However, we assume these cells are quiescent or dormant or they are part of stromal cells with a plastic potential to become KRT5 and KRT17 cells. Further, studies in the field describe that quiescent cells are normally identified by their low RNA content and their lack of cell proliferation markers which makes it difficult to identify them unless reactivated and initiate proliferation (Fukada et. al, 2007, PMID: 17600112; Cheung et al, 2013, PMID: 23698583).

Together, our data support the notion that these distinct progenitor cells (reserve cells) in endocervical tissue of human and mouse express KRT5 and KRT17 and remain dormant or quiescent but may be activated and start proliferating in a WNT inhibitory microenvironment in both humans and mice, similarly.

Moreover, we observed KRT7 not to be a distinct cell marker of the TZ, as it is mainly expressed in the endocervical columnar epithelium and sporadically also in the ectocervical epithelium (Figure 6E-F).

Finally, the anatomical and structural differences of the studied species is explained in the legend of Figure 1A: ‘In humans and mice, the female reproductive tract consists of ovaries, fallopian tubes, uterus, cervix and vagina. Anatomical differences between the two species include the bicornuate uterus (uterine horns) in mice while human have single ('simplex') uterus. However, both species possess a single cervix and vagina. While both species exhibit a typical ecto- and endocervix, the squamocolumnar junction in mice, unlike in humans, the endocervical and uterine boundary is not as pronounced.’

2) Single cell RNA-seq of endo- and ectocervix of metaplastic mice showed the expansion of squamous and myoepithelial cell clusters. What is the cell composition of the TZ in metaplastic mice? Can the authors detect keratin 17+ cells in the metaplastic endocervix? How about a keratin 8/p63/K5 expressing population, similar to that seen in human endocervix organoids grown in WNT-deficient medium (seen in figure 2)?

For single cell sequencing, we have taken the metaplastic region as a combined sample from three metaplastic mice. Metaplastic cells predominantly exhibit epithelial cell populations that are similar in part to healthy ectocervical tissue (Figure 5F). However, the various sub-populations of the metaplastic stroma are distinct from the endocervix stroma as well from ectocervix (Figure 5I). From this, we conclude that during metaplasia development extensive stromal remodeling occurs and altered stromal cues may drive squamous-like metaplasia.

We have found KRT17 expression in squamous metaplastic cells in the endocervix as shown by newly-added immunostaining (Ext. Data Fig. 5 M, O and Q). Also, please see the response to Comment 1.

Endocervical epithelial organoids grown in WNT-proficient or -deficient media express KRT8 but not p63/KRT5. However, the endocervical tissue-derived cells grown in the absence of WNT develop into stratified organoids and are p63/KRT5 positive but KRT8 negative. We find that KRT8 and KRT5/p63 are mutually exclusive in the columnar and squamous metaplastic cells (Ext. Data Fig. 5L, N and P). These data corroborate the

data shown by lineage tracing for KRT5 and KRT8 mice (Figure 1I-J) as well as immunostaining (Figure 1G-H) and in situ hybridizations (Ext. Data Fig. 1K-L). From this, we conclude that KRT8+ columnar epithelium that lines the endocervical mucosa does not transdifferentiate to p63/KRT5+ squamous epithelium. Instead, the endocervix tissue consists of progenitors of the p63/KRT5+ lineage that start proliferating under WNT inhibitory microenvironment and eventually replaces the columnar epithelium. Please also see response to Reviewer 1, point 9.

3) The authors observed that in the absence of WNT, human endocervix cells can give rise to p63+/K5+ stratified organoids, similar to those derived from ectocervix. Again, it would be good to clarify whether this is human specific. Can similar a phenomenon be seen in mouse endocervix organoids?

We see similar phenomenon in mouse as well as in human endocervical cells. We have now presented data for mouse endocervical cells that are grown in WNT-proficient or -deficient media (Ext. Data Fig. 2G) similar to human organoids (Figure 2A). Similar to human endocervix-derived cells (Figure 2E and Ext. Data Fig. 2I), mouse endocervix-derived cells also give rise to p63/KRT5+ stratified organoids in WNT-deficient media (Ext. Data Fig. 2H).

4) The single cell RNA-seq study revealed that distinctive stroma populations are detected in endocervix, ectocervix and TZ. A unique cluster of stromal cells is also identified in metaplastic mice. It will be important for the authors to investigate whether a similar phenomenon also exists in human endocervix, ectocervix and TZ using smRNA-FISH.

It would be interesting to check by smRNA-ISH whether the expression of WNT target genes in human samples is similar to mice. However, from our experience the preparation of sample is crucial for smRNA-ISH. The tissues have to be fixed as soon as possible after dissection/isolation to preserve the integrity of RNA so that it is detectable by ISH. For mouse tissue we could control this. However, due to the ongoing COVID-19 situation we do not readily have access to fresh human tissue that would allow rapid tissue preparation, compatible for RNA-ISH. Currently, it is unclear when this situation will change. Also, we believe that this aspect is not in the scope of this manuscript.

As demonstrated by our data, the growth of ectocervix-derived organoids from both human and mouse does not depend on WNT while the endocervix derived organoids from human and mouse have similar requirements of WNT agonist. This clearly indicates that the stromal WNT microenvironment is similar in both systems. Further, experiments with human organoids using recombinant DKK2 (shown in Figure 3E-H) has clearly demonstrated that presence of DKK2 in the organoid medium significantly decreases the endocervical organoid size, while having no effect on ectocervical organoids. We have also added additional data in the revised manuscript to further demonstrate the role of cell-autonomous WNT signaling in human organoid system.

Now we inhibit the cell-autonomous WNT signaling by using IWP2, a well characterized selective small molecule inhibitor of WNT signaling. IWP2 inhibits the processing and secretion of WNTs. It inactivates PORCN, a membrane-bound O-acyltransferase (MBOAT), and selectively inhibits palmitoylation of WNT, and it blocks WNT-dependent phosphorylation of LRP6 receptor and DVL2, and β-catenin accumulation (Baozhi Chen et al, 2009, PMID: 19125156). To further corroborate the role of cell-autonomous WNT signaling in our organoid system, we treated organoids with IWP2. The ectocervical and endocervical organoids were grown with or without IWP2 in the medium, after which their growth ability was quantified. Data are now presented as graph (Figure 3F-G) and representative IHC images are included (Figure 3H). The data clearly demonstrate that the treatment of ectocervical organoids with IWP2 does not influence their growth and is comparable to ectocervical organoids grown in the absence of WNT3A and Rspo1. In contrast, ectocervical organoids grown in the presence of WNT3A and Rspo1 are significantly smaller. On the other hand, endocervical organoids treated with IWP2 are significantly smaller in size compared to control endocervical organoids grown in media containing WNT3A and Rspo1. These new results are in line with the treatment of organoids with the WNT inhibitor DKK2 (Figure 3E).

Taken together, our data clearly demonstrate that the ectocervical and endocervical epithelia are controlled by opposing WNT signaling.

5) Mouse linage tracing experiments confirmed that keratin 5+ cells will give rise to squamous epithelium whereas keratin 8+ cells will generate columnar epithelium in vivo. Single molecule RNA-FISH also confirmed that keratin 5 and keratin 8 are detected in squamous and columnar epithelial cells in human and mouse cervix sections. These results are expected and not surprising. If only a few K5+/p63+/K8+ cells exist in the transition zone, is the linage tracing method sensitive enough to pick them up?

Genetic lineage tracing systems using the Cre-LoxP systems (reviewed in Kretzschmar et al, PMID: 22265400) are indeed shown to be capable of labelling stem cells that are quite few in number in different systems, e.g., adult hair follicle stem cells by Morris et al 2004, PMID: 15024388 and Lgr5 positive stem cells of different tissue, including intestine and stomach (Barker et al 2007, PMID: 17934449, Barker et al 2010, PMID: 20085740).

Our analysis with IHC, sm-RNA FISH etc., as presented in the original MS did not show any KRT5+ cells that are positive for KRT8. Further, we believe that even if there were very few KRT5+/p63+/KRT8+ cells we would have been able to capture them in one of the two different lineage tracing mice we used. However, this was not the case in our analysis. Our data, thus, strongly argue against the possibility of KRT8+ cells developing into to squamous stratified epithelia or KRT5+ cells giving rise to the columnar epithelia.

Also, please see the response to Comment 2.

6) This study seems to suggest that the so called subglandular reserve cells do not exist. The findings that distinct stromal cells are detected in the endocervix, ectocervix and TZ, and that a unique stromal cluster was specifically found in metaplastic mice, seem to suggest that epithelial metaplasia is controlled by the surrounding stromal cells. This agrees with the signalling pathways required to maintain the stemness and differentiation state of the organoids. If this is indeed the case and epithelial cell fate is determined by the agonists or/and antagonists secreted by stromal cells, then the authors should be able to demonstrate the causal effect of stromal cells using a co-culture system. One would expect that a co-culture of columnar endocervix epithelial organoids with the identified unique stromal cells derived from the ectocervix would convert columnar endocervix to squamous ectocervix and vice versa. Can this be experimentally tested and demonstrated?

Firstly, we would like to clarify that in line with the reviewer, we indeed believe the existence of such reserve progenitor cells. We propose that they can give rise to squamous metaplasia rather than that columnar cells could transdifferentiate into stratified epithelia. See reply to Comment 1 for detailed explanation.

While the reviewer agrees with the overall interpretation of our findings based on the presented data, he/she addresses an additional approach that could potentially serve to further corroborate these findings by establishing a stroma – epithelial coculture model. However, besides the huge challenges of such a new assay system, we would argue that it might not have any advantages over the already applied experimental approaches:

1. As pointed out in response to Comment 1, we would like to emphasize once more that our existing data clearly demonstrates that two distinct stem cells give raise to columnar and stratified lineages of the cervix. Further, we have also demonstrated that transdifferentiation of KRT8+ columnar endocervical epithelium to KRT5+ stratified epithelium and vice versa does not occur in the cervix.
2. The endocervix harbors both the columnar and the stratified squamous lineage-specific stem cells and the dominance of one over the other is determined by the microenvironment controlled by the spatially-defined stromal subpopulations. Additionally, the origin of the squamous progenitor cells (reserve cells) in the endocervix is still not clear. Our data shows that metaplasia is associated with an increase in myoepithelial cells that show characteristics of squamous epithelium (Figure 5E) as well as significant changes in the composition of stromal cells (Figure 5I). However, it remains unclear if any of these non-epithelial cells are indeed the cell of origin for the squamous progenitor (reserve cell).
3. Furthermore, our data shows that both ectocervix and endocervix have different sub-populations of stromal cells that express unique set of genes (Figure 5I).

Therefore, for performing appropriate co-culture experiments, one would first have to isolate and culture and characterize each of these stromal sub-populations from the ectocervix and endocervix. Next, one has to combine them in the right proportions and in correct spatial order for them to exert the effect similar to the in vivo effect. This would require considerable effort and the current 3D cell co-culture technological systems are

not at a stage to perform such spatially-defined culturing experiment. Although very interesting, establishing such a system is considered to be out of the scope of this manuscript.

Minor comments

1) The authors should state the numbers of single cells sequenced and analysed in each figure.

The number of cells sequenced is now stated in the methods section in lines 611 and 627. The number of cells analysed for each figure is now stated in methods section in lines 640-645.

2) A more comprehensive overview of the field will be needed for the introduction. The authors also should discuss reasons for the discrepancies between their findings and previously published work.

We have now modified the introduction and discussion further to provide a better overview.

Reviewer #3:

Remarks to the Author:

The manuscript Chumduri et al attempts to describe how stromal microenvironment controls homeostasis of the cervical squamo-columnar zone (aka or squamo-columnar junction or transition zone, TZ). It also touches on potential factors regulating the emergence of squamous metaplasia induced by vitamin A deficiency. The topics addressed in this study are important and reported finding may provide some additional information about interactions between squamous and columnar epithelia at the area of their junction. The finding that squamous and columnar epithelia derive from two distinct cell lineages is not entirely surprising. However, authors provide convincing lineage tracing results supporting this conclusion. Unfortunately, there is a number of serious concerns about the study in its present form.

We are aligned by the proposition of two distinct cell lineages. Yet, there is controversy in the field regarding this biology, wherein some claim for transdifferentiation of one cervical lineage to another (Herfs M et al., 2012, PMID: 22689991; Jian M et al., 2017, PMID: 29019984). Our study, therefore, puts a conclusive end to this controversy.

1) The delineation of endocervix, TZ and ectocervix used by authors is questionable. According to Extended Data Fig. 1, Fig. 5L and M, and Extended Data Fig. 3, the endocervix occupies around one third of the uterine horns. In Extended Data Fig. 5, areas shown as endocervix are located not far from the junction to oviduct! This cannot be accurate. Uterine horns, with an exception of a very small segment near the corpus, are lined by the endometrial epithelium supported by very distinct highly cellular endometrial stroma. Such endometrial stroma is present in many so-called "endocervical" images of this study. TZ seems to be placed too distal

(too high in horn) to the actual transition zone. In some images (see e.g., Extended Data Fig. 3E), ectocervix in red box appears to be mostly vagina, although in the upper left corner of box it includes a bit of the ectocervix. Given this situation, it is unclear whether the current study accurately investigates endocervical biology. Were organoids also derived from the same generously defined "endocervix" regions?

The endometrium and the endocervix are different tissues, despite the fact they share some similar phenotypic features, such as a columnar epithelium. Thank you for emphasizing it. Although some histologies were done in the uterine tissue as examples for the endocervix, we now show images taken from the endocervix (more info below). Nonetheless, we would like to point out that endocervical tissue samples and organoids were unambiguously derived from the endocervix or TZ and not from the uterine horns. Thus, all single-cell analyses and organoid comparisons matched their respective tissues in an unbiased manner. The histology was an exception. This is how we perform the dissection:

Once the female reproductive tract (FRT) is dissected out from the mouse, we separate the ectocervix, TZ and endocervix based on the physical characteristic of the tissue as follows. The ectocervix is first located based on its proximity to the uterine horn. The tissue texture of the ectocervix is hard, dense and fibrous unlike the columnar endocervix and uterine horns. In mice, there is no clear anatomical definition of where the endocervix ends and uterine epithelium begins. Therefore, we chose the region close to the place of uterine horns bifurcation and dissect endocervical tissue to avoid contamination from uterine horn. Since, this is a smaller segment of the tissue there is also relatively small number of cells, which is also reflected in relatively small cell number sequenced in endocervix compared to TZ and ectocervical regions. Distal to the ectocervix lies the vagina which is hollow and not hard. We dissect out an area that physically overlaps with the defined ectocervix and the endocervix that extends towards the uterine horns. Thus, the adjoined TZ is included within the obtained tissue sample (Figure 1A). Further, the vaginal part was left out from the ectocervical bulb region.

Regardless of anatomical tissue definitions, the sm-RNA ISH is similar in the endocervix and uterine horns, at least with respect to the expression of analyzed genes (Ext. Data Fig. 1 K-L, Ext. Data Fig. 3). Further, IHC and lineage tracing analysis also revealed that columnar epithelium of both endocervix and uterine horns express similar markers (Figure 1J, Ext. Data Fig. 1I-J). Interestingly, we observed the emergence of metaplasia throughout the uterine horn starting from the TZ (Figure 5L-O, Ext. Data Fig. 5 G-I). This is in line with the previous studied employing Vitamin A deficient diet in mouse (Ponnamperuma et al., 1999,PMID: 10500019) and prolonged estrogen treatment in rats that resulted in the squamous metaplasia of the uterus (Girtlin N., 1954, PMID: 13218326).

To be unbiased, we have presented histology images of the entire mouse FRT except for the fallopian tube and the ovaries. Further, we have presented magnifications of certain regions from the endo, TZ and ectocervix as examples to illustrate the differences in the microenvironment. As pointed out by the reviewer, in certain instances the endocervix magnifications were previously in the area of the uterus. In the revised

version, we selected better anatomical locations for these magnifications, representing the endocervix, TZ and the ectocervix. Please see new insets (for example, in Ext. Data Fig. 3C-E and Ext. Data Fig. 5 G-I).

Essential aspects of these clarifications have now been included in the legend to Figure 1A.

2) Authors state that they combined cells from each region from 3 mice. Female reproductive tract undergoes significant changes during estrous cycle. At which stage mouse material has been collected?

As stated in the methods section for scRNA sequencing, we have combined cells from three mice for each tissue region. Tissues were collected at the age of 14-weeks-old irrespective of the estrous cycle. This is also true for mice fed with vitamin A-deficient diet. This time point is fixed; therefore, we assume that fluctuations in the estrous cycle are less likely to occur, but we cannot rule out such differences. Since our mice experiments span several estrous cycles, we have not evaluated if vitamin A-deficient diet has an influence on the estrous cycle and vice versa and, if the development of metaplasia depends on the stage of estrous cycle. While our hypothesis and study design do not intend to address the role of the estrous cycle we cannot interpret on this further.

3) Based on UMAP map of epithelial cells in Fig 1, it seems that the number of endocervical cells is smaller than that of TZ cells. Given that TZ is a very minute structure, this seems to be surprising. How did authors verify the accuracy of their material dissection?

Our immunostaining or in-situ hybridizations experiments confirm the accuracy of our careful way of dissecting out the tissue (see description under Comment 1).

Once the TZ is dissected out, it contains also some cells from overlapping regions of the ectocervix and endocervix. We would like to point out that data points in the UMAP may overlap each other resulting in seemingly weaker representation of cells within a cluster than there really are.

4) Authors' conclusions about the opposing roles of WNT signals from the stroma are based on testing WNT growth factors in organoid assays and descriptive tissue phenotyping. These observations are important but offer only a circumstantial evidence. Firm conclusions would require testing of WNT signaling by its direct genetic interrogation (KO or KD of genes in question) in organoid systems and in mouse models.

As nicely suggested by the reviewer, the employment of gene KO/KD would facilitate the detailed understanding of the contribution of each of these WNT regulators in tissue homeostasis, epithelial differentiation capacities and the process of metaplasia. Given the involvement of different WNT regulators in our study, one would have to generate

distinct sets of gene KO/KD in organoids or construct mouse models with inducible KO of the gene of interest. The inducible gene KO would have to be restricted to epithelial or stromal subpopulations in order to decisively understand their role in cervical TZ homeostasis and metaplasia development. Alternatively, one could establish a co-culture model of genetically manipulated stromal cells, on top of which endocervical/ectocervical organoids grow. Such a co-culture model does not yet exist. Given that the development of such models, followed by robust analysis, would require a massive amount of work and time, we believe that it is well beyond the scope of this manuscript. We aim to address these aspects using genetic interrogation in future studies.

Having said that, we do not consent with the reviewer's notion, depicting the presented evidence as circumstantial. Rather, within the respective constraints of the experimental approaches applied, they are conclusive and even causally informative. Besides identifying those WNT regulators and validating their spatial expression in the squamocolumnar junction and its underlying stroma, we have demonstrated that they actually influence the development of squamous and columnar epithelium in vitro using human and mouse organoids. Sets of experiments on organoids that evaluate the influence of WNT signals or their absence are presented throughout the manuscript. Furthermore, we demonstrate that in a mouse model of metaplasia, there are alterations in the WNT microenvironment, leading to an invasion of squamous epithelium within the endocervix and uterine horn.

Nonetheless, following your advice to target WNT signaling, instead of targeting individual genes we used a small molecule inhibitor of WNT signaling termed IWP2. IWP2 inhibits the processing and secretion of WNTs. It inactivates Porcn, a membrane-bound O-acyltransferase, and selectively inhibits palmitoylation of WNT, and it blocks WNT-dependent phosphorylation of Lrp6 receptor and Dvl2, and β-catenin accumulation (Chen et al, 2009, PMID: 19125156). To further corroborate the role of cell-autonomous WNT signaling in our organoid system, we treated organoids with IWP2. The ectocervical and endocervical organoids were grown with or without IWP2 in the medium, after which their growth ability was quantified. Data are now presented as graph (Figure 3F-G) and representative IHC images are included (Figure 3H). The data clearly demonstrate that the treatment of ectocervical organoids with IWP2 does not influence their growth and is comparable to ectocervical organoids grown in the absence of WNT3A and RSPO1. In contrast, ectocervical organoids grown in the presence of WNT3A and RSPO1 are significantly smaller. On the other hand, endocervical organoids treated with IWP2 are significantly smaller in size compared to control endocervical organoids grown in media containing WNT3A and RSPO1. These new results are in line with the treatment of organoids with the WNT inhibitor DKK2 (Figure 3E).

Taken together, our data clearly demonstrate that the ectocervical and endocervical epithelia are controlled by opposing WNT signals.

5) The study does not present any evidence that squamous and columnar epithelia derive from lineage-specific stem cell populations in vivo. It only shows that squamous and columnar epithelia derive from 2 specific cell lineages. For the same reason, the statement about "outgrowth of quiescent ectocervical stem cells" is questionable.

This concept of single stem cell-derived organoids is well accepted in the field of stem cell biology. Organoids originate from stem cells of the derived primary tissue; under favorable conditions they self-renew, differentiate and self-organize into a 3D cellular structure that mimics the respective tissue (Simian et al, 2017, PMID: 28031422). Differentiated cells, therefore, do not form organoids.

The fact that we can grow two distinct types of epithelial organoids, stratified squamous and columnar, under unmistakable different media settings, strongly imply two distinct stem cell populations. Once we change these media conditions, they are unable to transdifferentiate into the reciprocal type of epithelial lineage. It is also evident that a few ectocervical-like stem cells within the endocervix can form stratified organoids (Figure 2E right-hand side and Ext. Data 2H right hand side), which are similar to organoids derived from the ectocervix and dissimilar as compared to endocervical organoids. These are believed to originate from the subcolumnar reserve stem cells (see answers to Reviewer 2 and Ext. Data Fig. 5K). They can give rise to the emerging metaplasia (Ext. Data Fig. 5Q), which arises as an outgrowth into the endocervix (Figure 5L-O and Ext. Data 5P-Q). Finally, we demonstrate that these two lineages do not transdifferentiate either in vivo using the lineage tracing experiments (Figure 1I-J) or in vitro using organoids derived from the in vivo labelled cells (Figure 5A). Thus, columnar and squamous organoids represent the two lineage-specific stem cells with distinct characteristics.

Since we have not analyzed the cell cycle of the stem cells in vivo, we removed the term “quiescent” from the text.

6) Statement that "SCJ cells are not distinct from the endocervical columnar lineage and not the cells of origin of SCC" is speculative. Accurate in vivo modeling of such disease in required for such strong conclusion.

The study by Herfs et al., 2012 proposed the SCJ cells as the cell of origin for both SCC and cervical adenocarcinomas based on IHC of KRT7, CD63, GDA, MMP 7 and AGR2. The authors of this study observed the SCJ cells to be exclusively in the TZ in the adult human cervix and the same markers were then found by IHC in both type of cancers. However, our IHC and smRNA-ISH of healthy human and mouse cervix demonstrated that these cells are not restricted to the SCJ alone. These markers commonly characterize endocervical cells and not only the TZ (Figure 6E-F and Ext. Data Fig. 6F and J). Further, our unbiased analysis of comparing endocervical and ectocervical organoid-derived signatures to cervical cancers also clearly shows that these genes are highly expresses in cervical adenocarcinomas as compared to SCC (as shown in Figure 6A-B). Accordingly, it is highly unlikely that the SCJ cells are the origin of both cancers.

However, to make it clear for the reader, we have now addressed this in the text of the revised MS. In lines 360-362, 370-371 and 404-405, we have cautiously added that it is 'indicative of the cell of origin of these cancers.

Minor issues:

1) Has single cell transcriptome experiment been done on a single sample of 3 pooled mice or was it repeated 3 times?

Each tissue region, either from the ectocervix, endocervix or the TZ, represents pooling of three different mice. However, each region sample was sequenced separately.

2) How many cells were loaded on 10X Chromium controller?

Approximately 13,200 cells per sample were loaded on to the controller. The same is now mentioned in the methods section of the manuscript.

3) Generation of cell lineage trajectories by Monocle or some similar program could strengthen this study.

Trajectory analysis: Our analysis already captures the differentiation (trajectory) of the squamous epithelium. In contrast, the columnar epithelium forms a distinct cluster where differentiation could not be captured due to the low number of cells.

We have applied monocle and slingshot to the subset of healthy squamous epithelium and successfully recovered the differentiation. However, we did not see added value in applying these algorithms to the whole dataset since we could very well separate these biological signals using the UMAP algorithm and SLM clustering.

Author Rebuttal to Initial comments 2

Reviewer #1

Remarks to the Author
The authors have satisfactorily addressed the initial concerns.

Thank you for your support. Hopefully, this article ends as a publication in *Nature Cell Biology*.

Reviewer #2

Remarks to the Author
I have now read the revised manuscript and I am pleased to say that the authors have carried out extensive revision. The manuscript is much improved and all my queries have been clarified and I am happy to recommend its publication in NCB.

Thank you for your support. Hopefully, this article ends as a publication in *Nature Cell Biology*.

Reviewer #3

Remarks to the Author
The manuscript has been significantly improved. However, some concerns remain:

1) Fig.1A: As compared to their original Ext. Fig. 1A, the authors moved the border between endocervix and endometrium towards TZ in the mouse. The drawing of female reproductive tract in Fig. 1A is identical to that in paper by Tiffany Brake and Paul Lambert (Fig. 3; PNAS 102: 2490-2495, 2005). However, newly assigned anatomical demarcations differ from the original. These new boundaries are also different from other studies (e.g., by Elson et al., Cancer Res 60: 1267–1275, 2000). To avoid further confusion, authors need to provide some references or explanations how they have arrived to their new anatomical demarcations.

- We appreciate your positive feedback.

- The initial drawing of the mouse female reproductive tract has been adapted from PNAS 102: 2490-2495, 2005. However, this article did not distinguish the subsections of endo-ecto, and TZ of the cervix. Therefore, we have defined these borders based on the physical characteristic of the tissue (response to Referee 3, comment 1, from the previous point by point response) as well as our unbiased analysis of the female genital tract immunostainings for epithelial marker E-cadherins that enable visualization of the squamous stratified region and the TZ (data not included). The immediate columnar epithelium adjacent to the TZ is dissected out as endocervix. The purpose of this cartoon is to simplify understanding by the readers. It was further slightly modified to reflect how the tissue was carefully dissected for single-cell analysis.

- We have now included an explanation with a citation to the article in PNAS in our methods section on page 24-25: "The female reproductive tract and cervix illustration shown in Fig. 1A was outlined based on a previous scheme (PMID: 15699322). Further, ectocervix, TZ and endocervix borders were defined based on the physical characteristic of the tissue as well as the epithelial marker E-cadherins that facilitate visualization of ectocervical squamous stratified, endocervix columnar epithelium and the TZ (data not shown). The ectocervix was first located based on its proximity to the uterine horn. The tissue texture of the ectocervix is hard, dense and fibrous, unlike the columnar endocervix and uterine horns. In mice, there is no clear anatomical definition of where the endocervix ends and uterine epithelium begins. Therefore, we chose the region close to the place of uterine horns bifurcation and a smaller segment of the endocervical tissue to avoid contamination from the uterine horn. Distal to the ectocervix lies the vagina, which is hollow and not hard. We dissected out an area that physically overlaps with the defined ectocervix and the endocervix that extends towards the uterine horns. Thus, the adjoined TZ is included within the obtained tissue sample (Fig. 1A). Further, the vaginal part was left out from the ectocervical bulb region.

2) Fig. 5 M Ext Figure 3 B, D, H, and 6 F: "Endocervix" images still show highly cellular endometrial stroma characteristic for endometrium and not endocervix.

- We have now included also an additional region near the TZ, as shown in Figure 5L and M. The endocervical magnifications have been changed to a region nearer to the TZ, as shown in Ext. Data Figure 3B, D, H, and 6F.

3) In their response to comment #5 by reviewer #3, authors seem to equalize organoid cell culture to physiological conditions in vivo. Organoid cell cultures are important tool in stem cell biology. Unfortunately, it is well established that, even under the most optimal conditions, ex vivo cells behave differently from those in vivo. Thus, certain caution should be applied during interpretation of organoid studies.

- We agree with the referee that the in vivo conditions are far more complex than those in vitro, considering the interplay between various cell types. Therefore, to complement the organoid cultures, we have also demonstrated that the cervix harbors two distinct epithelial lineage stem cells by employing in vivo lineage-tracing techniques and single-cell analyses. Both these approaches unambiguously showed that the squamous lineage and the columnar lineage are driven by distinct stem cells and that the metaplastic cells resemble the squamous lineage. Additionally, the IHC and RNA-ISH data complement the organoid growth factor requirement data, as they clearly demonstrate the divergent signals underneath these two epithelia. Thus, our approach of utilizing different complementary technologies has yielded a more realistic picture of epithelial homeostasis at the cervical transition zone.

5) line 89: "adult epithelial stem cell lineages". What are those? Cell lineages usually consist of stem cells and their more differentiated progeny.

- We have now altered the sentence into "Our study unravels the cellular subsets of the cervix and reveals two committed adult epithelial stem cells, giving rise to squamous and columnar epithelial lineages."

In addition, it is a limitation that the estrous cycle was not taken into account during the analysis. This should be discussed in the manuscript and caveats noted.

- As mentioned in our previous response to comment #2 from reviewer #3, the murine estrous cycle repeats every 3-4 days. In our study, all the experimental vitamin A-deficient diet treated mice have been analyzed 14 weeks after their birth. According to this analysis, the metaplasia development was connected clearly to the diet and alteration in the Wnt signaling. Within our experimental setup, we have not analyzed the effect of Vitamin A-deficient diet and altered Wnt signaling on the estrous cycle.
- Previously, Elson et al., Cancer Res 60: 1267–1275, 2000, have observed that prolonged treatment of K14-HPV16 transgenic mice with estrogen leads to multifocal glandular squamous metaplasia in the lower uterus, which progress into high-grade dysplasia and invasive cancers. Thus, they corroborate estrogen as a cofactor in the development of cervical cancer.
- Inhibition of retinoic acid signaling might directly or indirectly influence the estrous cycle through cross-talk with Wnt signaling. It would be interesting to understand further the relation between retinoic acid, Wnt signaling and estrous cycle during the development of squamous metaplasia. However, to make any statement regarding the role of the estrous cycle in the maintenance of cervical squamocolumnar junction in healthy mice and during

metaplasia development, further careful and systematic analysis is warranted. This is beyond the scope of this manuscript.

- However, we have now discussed this point in the the manuscript on page 16: "Estrogen, one of the key regulators of the estrous cycle, acts as a cofactor during the HPV-driven cervical carcinogenesis (PMID: 15699322). It is likely that there is an interplay between the estrous cycle and Wnt signaling at the TZ; it is important to understand how it affects metaplasia and cancer development."

Loading controller with 13,200 cells per sample might lead to over 5% in cell doublets according to 10X genomics. Details should be provided how the inclusion of cell doublets was prevented.

- To remove doublets, we first excluded, in silico, the cell barcodes associated with a high number of transcripts (the thresholds are already indicated in the Methods section). Secondly, we used the tool Scrublet from the scran package (1.14.6) (Wolock et al., Cell Systems, 2019; PMID: 30954476) to calculate a numeric score to identify doublets. Third, we rely on marker gene expression to assess if a hybrid transcriptome could mimic the cell type in focus. For example, in the case of our Dkk2-positive stromal subpopulation, there might be a subpopulation of cells with high expression of Dkk2 that could mimic the Dkk2-positive stromal phenotype when forming a doublet with a stromal cell. However, no such population exists in the datasets, where we have substantial Dkk2 expression in the stroma (Datasets IDs: G10, G12, H1).

- We have now included the information in the Methods section of the manuscript on page 26: "The expression matrices were filtered and potential doublets were removed by excluding barcodes with fewer than 250 genes, more than 4,000 genes and more than 15.000 Unique Molecule Identifiers (UMIs). Additionally, barcodes with more than 10 percent mitochondrial genes detected were excluded. Genes that were not detected for any barcode were also removed. Potential doublets were also scrutinized using Scrublet from the scran package (1.14.6) (Wolock et al 2019 Cell Systems; PMID: 30954476) and we used marker gene expression to assess if the cell type in focus could be mimicked by a hybrid transcriptome, but these analysis did not lead to further removal of cell-associated barcodes."

Decision Letter, first revision:

Date: 4th September 20 11:17:23
Last Sent: 4th September 20 11:17:23
Triggered By: Christine Weber
From: christine.weber@nature.com
To: tfm@mpiib-berlin.mpg.de
CC: edmund.irwin@nature.com
Subject: Decision on Nature Cell Biology submission NCB-M41689A

Message: *Please delete the link to your author homepage if you wish to forward this email to co-authors.

Dear Thomas,

Thank you for responding to the referee comments on your revised manuscript, "Opposing Wnt signals regulate cervical squamocolumnar homeostasis and emergence of metaplasia". As communicated to you previously, referees 1 and 2 now recommend publication of the manuscript, whereas referee 3 has noted some persisting concerns. We remain very interested in this study, but we believe that these concerns should be addressed in full before we can consider publication in Nature Cell Biology.

In particular, it will be essential to address the concerns raised by referee 3 as outlined in your latest rebuttal. However, please note that we cannot accept statements that refer to data not shown in the manuscript (point 1). Please ensure that additional images are provided for full clarification of how anatomical structures were defined. For point 3, please also acknowledge relevant caveats and limitations in the text.

Once we receive these revisions, we will return them to referee 3 for a final assessment and further consideration of this manuscript will depend on the referee being fully satisfied.

As before, please pay close attention to our guidelines on statistical and methodological reporting (listed below) as failure to do so may delay the reconsideration of the revised manuscript.

We therefore invite you to take these points into account when revising the manuscript. In addition, when preparing the revision please:

- ensure that it conforms to our format instructions and publication policies (see below and www.nature.com/nature/authors/).

- provide an updated source data table.

- provide a point-by-point rebuttal to the full referee reports verbatim, as provided at the end of this letter.

- provide an updated Editorial Policy Checklist (found here https://www.nature.com/authors/policies/Policy.pdf) and Reporting Summary (found here https://www.nature.com/authors/policies/ReportingSummary.pdf). This is essential for reconsideration of the manuscript and these documents will be available to editors and referees in the event of peer review. For more information see http://www.nature.com/authors/policies/availability.html or contact me.

Please submit the revised manuscript files and the point-by-point rebuttal to the referee comments using this link:

[REDACTED]

*This url links to your confidential home page and associated information about manuscripts you may have submitted or be reviewing for us. If you wish to forward this email to co-authors, please delete the link to your homepage.

We would like to receive the revision within six weeks. If submitted within this time period, reconsideration of the revised manuscript will not be affected by related studies published elsewhere, or accepted for publication in Nature Cell Biology in the meantime. We would be happy to consider a revision even after this timeframe, but in that case we will consider the published literature at the time of resubmission when assessing the file.

We hope that you will find our referees' comments, and editorial guidance helpful. Please do not hesitate to contact me if there is anything you would like to discuss.

With best wishes,

Christine.

Christine Weber, PhD
Senior Editor
Nature Cell Biology
E-mail: christine.weber@nature.com
Phone: +44 (0)207 843 4924

Reviewers' Comments:

Reviewer #1:
Remarks to the Author:
The authors have satisfactorily addressed the initial concerns.

Reviewer #2:
Remarks to the Author:
I have now read the revised manuscript and I am pleased to say that the authors have carried out extensive revision. The manuscript is much improved and all my queries have been clarified and I am happy to recommend its publication in NCB.

Reviewer #3:
Remarks to the Author:
The manuscript has been significantly improved. However, some concerns remains:

1) Fig.1A: As compared to their original Ext. Fig. 1A, the authors moved the border between endocervix and endometrium towards TZ in the mouse. The drawing of female reproductive tract in Fig. 1A is identical to that in paper by Tiffany Brake

and Paul Lambert (Fig. 3; PNAS 102: 2490-2495, 2005). However, newly assigned anatomical demarcations differ from the original. These new boundaries are also different from other studies (e.g., by Elson et al., Cancer Res 60: 1267–1275, 2000). To avoid further confusion, authors need to provide some references or explanations how they have arrived to their new anatomical demarcations.

2) Fig. 5 M Ext Figure 3 B, D, H, and 6 F: "Endocervix" images still show highly cellular endometrial stroma characteristic for endometrium and not endocervix.

3) In their response to comment #5 by reviewer #3, authors seem to equalize organoid cell culture to physiological conditions in vivo. Organoid cell cultures are important tool in stem cell biology. Unfortunately, it is well established that, even under the most optimal conditions, ex vivo cells behave differently from those in vivo. Thus, certain caution should be applied during interpretation of organoid studies.

5) line 89: "adult epithelial stem cell lineages". What are those? Cell lineages usually consist of stem cells and their more differentiated progeny.

In addition, it is a limitation that the estrous cycle was not taken into account during the analysis. This should be discussed in the manuscript and caveats noted.

Loading controller with 13,200 cells per sample might lead to over 5% in cell doublets according to 10X genomics. Details should be provided how the inclusion of cell doublets was prevented.

GUIDELINES FOR SUBMISSION OF NATURE CELL BIOLOGY ARTICLES

READABILITY OF MANUSCRIPTS – Nature Cell Biology is read by cell biologists from diverse backgrounds, many of whom are not native English speakers. Authors should aim to communicate their findings clearly, explaining technical jargon that might be unfamiliar to non-specialists, and avoiding non-standard abbreviations. Titles and abstracts should concisely communicate the main findings of the study, and the background, rationale, results and conclusions should be clearly explained in the manuscript in a manner accessible to a broad cell biology audience. Nature Cell Biology uses British spelling.

ARTICLE FORMAT

TITLE – should be no more than 100 characters including spaces, without punctuation and avoiding technical terms, abbreviations, and active verbs..

AUTHOR NAMES – should be given in full.

AUTHOR AFFILIATIONS – should be denoted with numerical superscripts (not symbols) preceding the names. Full addresses should be included, with US states in full and providing zip/post codes. The corresponding author is denoted by: "Correspondence should be addressed to [initials]."

ABSTRACT – should not exceed 150 words and should be unreferenced. This paragraph is the most visible part of the paper and should briefly outline the background and rationale for the work, and accurately summarize the main results and conclusions. Key genes, proteins and organisms should be specified to ensure discoverability of the paper in online searches.

TEXT – the main text consists of the Introduction, Results, and Discussion sections and must not exceed 3500 words including the abstract. The Introduction should expand on the background relating to the work. The Results should be divided in subsections with subheadings, and should provide a concise and accurate description of the experimental findings. The Discussion should expand on the findings and their implications. All relevant primary literature should be cited, in particular when discussing the background and specific findings.

ACKNOWLEDGEMENTS – should be kept brief. Professional titles and affiliations are unnecessary. Grant numbers can be listed.

AUTHOR CONTRIBUTIONS – must be included after the Acknowledgements, detailing the contributions of each author to the paper (e.g. experimental work, project planning, data analysis etc.). Each author should be listed by his/her initials.

FINANCIAL AND NON-FINANCIAL COMPETING INTERESTS – the authors must include one of three declarations: (1) that they have no financial and non-financial competing interests; (2) that they have financial and non-financial competing interests; or (3) that they decline to respond, after the Author Contributions section. This statement will be published with the article, and in cases where financial and non-financial competing interests are declared, these will be itemized in a web supplement to the article. For further details please see https://www.nature.com/licenceforms/nrg/competing-interests.pdf.

REFERENCES – are limited to a total of 70 in the main text and Methods combined,. They must be numbered sequentially as they appear in the main text, tables and figure legends and Methods and must follow the precise style of Nature Cell Biology references. References only cited in the Methods should be numbered consecutively following the last reference cited in the main text. References only associated with Supplementary Information (e.g. in supplementary legends) do not count toward the total reference limit and do not need to be cited in numerical continuity with references in the main text. Only published papers can be cited, and each publication cited should be included in the numbered reference list, which should include the manuscript titles. Footnotes are not permitted.

METHODS – Nature Cell Biology publishes methods online. The methods section should be provided as a separate Word document, which will be copyedited and

appended to the manuscript PDF, and incorporated within the HTML format of the paper.

Methods should be written concisely, but should contain all elements necessary to allow interpretation and replication of the results. As a guideline, Methods sections typically do not exceed 3,000 words. The Methods should be divided into subsections listing reagents and techniques. When citing previous methods, accurate references should be provided and any alterations should be noted. Information must be provided about: antibody dilutions, company names, catalogue numbers and clone numbers for monoclonal antibodies; sequences of RNAi and cDNA probes/primers or company names and catalogue numbers if reagents are commercial; cell line names, sources and information on cell line identity and authentication. Animal studies and experiments involving human subjects must be reported in detail, identifying the committees approving the protocols. For studies involving human subjects/samples, a statement must be included confirming that informed consent was obtained. Statistical analyses and information on the reproducibility of experimental results should be provided in a section titled "Statistics and Reproducibility".

All Nature Cell Biology manuscripts submitted on or after March 21 2016, must include a Data availability statement as a separate section after Methods but before references, under the heading "Data Availability". For Springer Nature policies on data availability see http://www.nature.com/authors/policies/availability.html; for more information on this particular policy see http://www.nature.com/authors/policies/data/data-availability-statements-data-citations.pdf. The Data availability statement should include:

• Accession codes for primary datasets (generated during the study under consideration and designated as "primary accessions") and secondary datasets (published datasets reanalysed during the study under consideration, designated as "referenced accessions"). For primary accessions data should be made public to coincide with publication of the manuscript. A list of data types for which submission to community-endorsed public repositories is mandated (including sequence, structure, microarray, deep sequencing data) can be found here http://www.nature.com/authors/policies/availability.html#data.

• Unique identifiers (accession codes, DOIs or other unique persistent identifier) and hyperlinks for datasets deposited in an approved repository, but for which data deposition is not mandated (see here for details http://www.nature.com/sdata/data-policies/repositories).

• At a minimum, please include a statement confirming that all relevant data are available from the authors, and/or are included with the manuscript (e.g. as source data or supplementary information), listing which data are included (e.g. by figure panels and data types) and mentioning any restrictions on availability.

• If a dataset has a Digital Object Identifier (DOI) as its unique identifier, we strongly encourage including this in the Reference list and citing the dataset in the Methods.

We recommend that you upload the step-by-step protocols used in this manuscript

to the Protocol Exchange. More details can found at www.nature.com/protocolexchange/about.

DISPLAY ITEMS – main display items are limited to 6-8 main figures and/or main tables. For Supplementary Information see below.

FIGURES – Colour figure publication costs $395 per colour figure. All panels of a multi-panel figure must be logically connected and arranged as they would appear in the final version. Unnecessary figures and figure panels should be avoided (e.g. data presented in small tables could be stated briefly in the text instead).

All imaging data should be accompanied by scale bars, which should be defined in the legend.
Cropped images of gels/blots are acceptable, but need to be accompanied by size markers, and to retain visible background signal within the linear range (i.e. should not be saturated). The boundaries of panels with low background have to be demarked with black lines. Splicing of panels should only be considered if unavoidable, and must be clearly marked on the figure, and noted in the legend with a statement on whether the samples were obtained and processed simultaneously. Quantitative comparisons between samples on different gels/blots are discouraged; if this is unavoidable, it has be performed for samples derived from the same experiment with gels/blots were processed in parallel, which needs to be stated in the legend.

Figures should be provided at approximately the size that they are to be printed at (single column is 86 mm, double column is 170 mm) and should not exceed an A4 page (8.5 x 11"). Reduction to the scale that will be used on the page is not necessary, but multi-panel figures should be sized so that the whole figure can be reduced by the same amount at the smallest size at which essential details in each panel are visible. In the interest of our colour-blind readers we ask that you avoid using red and green for contrast in figures. Replacing red with magenta and green with turquoise are two possible colour-safe alternatives. Lines with widths of less than 1 point should be avoided. Sans serif typefaces, such as Helvetica (preferred) or Arial should be used. All text that forms part of a figure should be rewritable and removable.

We accept files from the following graphics packages in either PC or Macintosh format:

- For line art, graphs, charts and schematics we prefer Adobe Illustrator (.AI), Encapsulated PostScript (.EPS) or Portable Document Format (.PDF). Files should be saved or exported as such directly from the application in which they were made, to allow us to restyle them according to our journal house style.

- We accept PowerPoint (.PPT) files if they are fully editable. However, please refrain from adding PowerPoint graphical effects to objects, as this results in them outputting poor quality raster art. Text used for PowerPoint figures should be Helvetica (preferred) or Arial.

- We do not recommend using Adobe Photoshop for designing figures, but we can

accept Photoshop generated (.PSD or .TIFF) files only if each element included in the figure (text, labels, pictures, graphs, arrows and scale bars) are on separate layers. All text should be editable in 'type layers' and line-art such as graphs and other simple schematics should be preserved and embedded within 'vector smart objects' - not flattened raster/bitmap graphics.

- Some programs can generate Postscript by 'printing to file' (found in the Print dialogue). If using an application not listed above, save the file in PostScript format or email our Art Editor, Allen Beattie for advice (a.beattie@nature.com).

Regardless of format, all figures must be vector graphic compatible files, not supplied in a flattened raster/bitmap graphics format, but should be fully editable, allowing us to highlight/copy/paste all text and move individual parts of the figures (i.e. arrows, lines, x and y axes, graphs, tick marks, scale bars etc). The only parts of the figure that should be in pixel raster/bitmap format are photographic images or 3D rendered graphics/complex technical illustrations.

All placed images (i.e. a photo incorporated into a figure) should be on a separate layer and independent from any superimposed scale bars or text. Individual photographic images must be a minimum of 300+ DPI (at actual size) or kept constant from the original picture acquisition and not decreased in resolution post image acquisition. All colour artwork should be RGB format.

FIGURE LEGENDS – must not exceed 350 words for each figure to allow fit on a single printed NCB page together with the figure. They must include a brief title for the whole figure, and short descriptions of each panel with definitions of the symbols used, but without detailing methodology.

TABLES – main tables should be provided as individual Word files, together with a brief title and legend. For supplementary tables see below.

SUPPLEMENTARY INFORMATION – Supplementary information is material directly relevant to the conclusion of a paper, but which cannot be included in the printed version in order to keep the manuscript concise and accessible to the general reader. Supplementary information is an integral part of a Nature Cell Biology publication and should be prepared and presented with as much care as the main display item, but it must not include non-essential data or text, which may be removed at the editor's discretion. All supplementary material is fully peer-reviewed and published online as part of the HTML version of the manuscript. Supplementary Figures and Supplementary Notes are appended at the end of the main PDF of the published manuscript.

Supplementary items should relate to a main text figure, wherever possible, and should be mentioned sequentially in the main manuscript, designated as Supplementary Figure, Table, Video, or Note, and numbered continuously (e.g. Supplementary Figure 1, Supplementary Figure 2, Supplementary Table 1, Supplementary Table 2 etc.).

Unprocessed scans of all key data generated through electrophoretic separation

techniques need to be presented in a supplementary figure that should be labeled and numbered as the final supplementary figure, and should be mentioned in every relevant figure legend. This figure does not count towards the total number of figures and is the only figure that can be displayed over multiple pages, but should be provided as a single file, in PDF or TIFF format. Data in this figure can be displayed in a relatively informal style, but size markers and the figures panels corresponding to the presented data must be indicated.

The total number of Supplementary Figures (not including the "unprocessed scans" Supplementary Figure) should not exceed the number of main display items (figures and/or tables (see our Guide to Authors and March 2012 editorial http://www.nature.com/ncb/authors/submit/index.html#suppinfo; http://www.nature.com/ncb/journal/v14/n3/index.html#ed). No restrictions apply to Supplementary Tables or Videos, but we advise authors to be selective in including supplemental data.

Each Supplementary Figure should be provided as a single page and as an individual file in one of our accepted figure formats and should be presented according to our figure guidelines (see above). Supplementary Tables should be provided as individual Excel files. Supplementary Videos should be provided as .avi or .mov files up to 50 MB in size. Supplementary Figures, Tables and Videos much be accompanied by a separate Word document including titles and legends.

GUIDELINES FOR EXPERIMENTAL AND STATISTICAL REPORTING

REPORTING REQUIREMENTS – To improve the quality of methods and statistics reporting in our papers we have recently revised the reporting checklist we introduced in 2013. We are now asking all life sciences authors to complete two items: an Editorial Policy Checklist (found here https://www.nature.com/authors/policies/Policy.pdf) that verifies compliance with all required editorial policies and a Reporting Summary (found here https://www.nature.com/authors/policies/ReportingSummary.pdf) that collects information on experimental design and reagents. These documents are available to referees to aid the evaluation of the manuscript. Please note that these forms are dynamic 'smart pdfs' and must therefore be downloaded and completed in Adobe Reader. We will then flatten them for ease of use by the reviewers. If you would like to reference the guidance text as you complete the template, please access these flattened versions at http://www.nature.com/authors/policies/availability.html.

STATISTICS – Wherever statistics have been derived the legend needs to provide the n number (i.e. the sample size used to derive statistics) as a precise value (not a range), and define what this value represents. Error bars need to be defined in the legends (e.g. SD, SEM) together with a measure of centre (e.g. mean, median). Box plots need to be defined in terms of minima, maxima, centre, and percentiles. Ranges are more appropriate than standard errors for small data sets. Wherever statistical significance has been derived, precise p values need to be provided and the statistical test used needs to be stated in the legend. Statistics such as error bars must not be derived from n<3. For sample sizes of n<5 please plot the individual data points rather than providing bar graphs. Deriving statistics

from technical replicate samples, rather than biological replicates is strongly discouraged. Wherever statistical significance has been derived, precise p values need to be provided and the statistical test stated in the legend.

Information on how many times each experiment was repeated independently with similar results needs to be provided in the legends and/or Methods for all experiments, and in particular wherever representative experiments are shown.

We strongly recommend the presentation of source data for graphical and statistical analyses as a separate Supplementary Table, and request that source data for all independent repeats are provided when representative experiments of multiple independent repeats, or averages of two independent experiments are presented. This supplementary table should be in Excel format, with data for different figures provided as different sheets within a single Excel file. It should be labelled and numbered as one of the supplementary tables, titled "Statistics Source Data", and mentioned in all relevant figure legends.

Author Rebuttal, first revision:

Reviewer #1

Remarks to the Author
The authors have satisfactorily addressed the initial concerns.

- Thank you for your support.

Reviewer #2

Remarks to the Author
I have now read the revised manuscript and I am pleased to say that the authors have carried out extensive revision. The manuscript is much improved and all my queries have been clarified and I am happy to recommend its publication in NCB.

- Thank you for your support.

Reviewer #3

Remarks to the Author
The manuscript has been significantly improved. However, some concerns remain:

1) Fig.1A: As compared to their original Ext. Fig. 1A, the authors moved the border between endocervix and endometrium towards TZ in the mouse. The drawing of female reproductive tract in Fig. 1A is identical to that in paper by Tiffany Brake and Paul Lambert (Fig. 3; PNAS 102: 2490-2495, 2005). However, newly assigned anatomical demarcations differ from the original. These new boundaries are also different from other studies (e.g., by Elson et al., Cancer Res 60: 1267–1275,

2000). To avoid further confusion, authors need to provide some references or explanations how they have arrived to their new anatomical demarcations.

- We appreciate your positive feedback.

- The initial drawing of the mouse female reproductive tract has been adapted from PNAS 102: 2490-2495, 2005. However, this article did not distinguish the subsections of endo-, ecto-, and TZ of the cervix. Therefore, we have defined these borders based on the physical characteristic of the tissue (response to Referee 3, comment 1, from the previous point by point response) as well as our unbiased analysis of the female genital tract immunostainings for epithelial marker E-cadherins that enable visualization of the squamous stratified region and the TZ (newly included data in: Fig. 1A bottom panels). Our new drawing now depicts the structure of the mouse genital tract more realistically, in accordance with the fluorescent picture of the same figure and many other figures provided in this manuscript (e.g. Fig.6F, Extended Data Fig.1J-L, Extended Data Fig.3). The blue demarcations include the TZ and served to physically dissect this region from the endo- and ectocervical tissue, e.g. for single-cell sequence analysis.

- We have now included an explanation of the anatomical demarcation in the Figure 1 A legend. "Mouse FGT schematic was outlined based on immunostainings with epithelial marker E-cadherin (bottom panels) and further microscopic images of different markers as shown in Fig.6F, Extended Data Fig.1J-L and Extended Data Fig.3."

2) Fig. 5 M Ext Figure 3 B, D, H, and 6 F: "Endocervix" images still show highly cellular endometrial stroma characteristic for endometrium and not endocervix.

- We have now included also an additional region near the TZ, as shown in Figure 5L and M. The endocervical magnifications have been changed to a region nearer to the TZ, as shown in Ext. Data Figure 3B, D, H, and 6F.

3) In their response to comment #5 by reviewer #3, authors seem to equalize organoid cell culture to physiological conditions in vivo. Organoid cell cultures are important tool in stem cell biology. Unfortunately, it is well established that, even under the most optimal conditions, ex vivo cells behave differently from those in vivo. Thus, certain caution should be applied during interpretation of organoid studies.

- We agree with the referee that the in vivo conditions are far more complex than those in vitro, considering the interplay between various cell types. Therefore, to complement the organoid cultures, we have also demonstrated that the cervix harbors two distinct epithelial lineage stem cells by employing in vivo lineage-tracing techniques and single-cell analyses. Both these approaches unambiguously showed that the squamous lineage and the columnar lineage are driven by distinct stem cells and that the metaplastic cells resemble the squamous lineage. Additionally, the IHC and RNA-ISH data complement the organoid growth factor requirement data, as they clearly demonstrate the divergent signals underneath these two epithelia. Thus, our approach of utilizing different complementary technologies has yielded a more realistic picture of epithelial homeostasis at the cervical transition zone.

Now we have included the following sentences to bring to the reader's notice about the possible in vitro and in vivo differences on Page 17.

"Nevertheless, organoids may not fully recapitulate the multifaceted interactions between various cell types in a tissue and represent an approximation of an even greater complexity in vivo."

5) line 89: "adult epithelial stem cell lineages". What are those? Cell lineages usually consist of stem cells and their more differentiated progeny.

- We have now altered the sentence into "Our study unravels the cellular subsets of the cervix and reveals two committed adult epithelial stem cells, giving rise to squamous and columnar epithelial lineages."

In addition, it is a limitation that the estrous cycle was not taken into account during the analysis. This should be discussed in the manuscript and caveats noted.

- As mentioned in our previous response to comment #2 from reviewer #3, the murine estrous cycle repeats every 3-4 days. In our study, all the experimental vitamin A-deficient diet treated mice have been analyzed 14 weeks after their birth. According to this analysis, the metaplasia development was connected clearly to the diet and alteration in the Wnt signaling. Within our experimental setup, we have not analyzed the effect of Vitamin A-deficient diet and altered Wnt signaling on the estrous cycle.
- Previously, Elson et al., Cancer Res 60: 1267–1275, 2000, have observed that prolonged treatment of K14-HPV16 transgenic mice with estrogen leads to multifocal glandular squamous metaplasia in the lower uterus, which progress into high-grade dysplasia and invasive cancers. Thus, they corroborate estrogen as a cofactor in the development of cervical cancer.
- Inhibition of retinoic acid signaling might directly or indirectly influence the estrous cycle through cross-talk with Wnt signaling. It would be interesting to understand further the relation between retinoic acid, Wnt signaling and estrous cycle during the development of squamous metaplasia. However, to make any statement regarding the role of the estrous cycle in the maintenance of cervical squamocolumnar junction in healthy mice and during metaplasia development, further careful and systematic analysis is warranted. This is beyond the scope of this manuscript.

- However, we have now discussed this point in the the manuscript on page 16: "Estrogen, one of the key regulators of the estrous cycle, acts as a cofactor during the HPV-driven cervical carcinogenesis (PMID: 15699322). It is likely that there is an interplay between the estrous cycle and Wnt signaling at the TZ; it is important to understand how it affects metaplasia and cancer development."

Loading controller with 13,200 cells per sample might lead to over 5% in cell doublets according to 10X genomics. Details should be provided how the inclusion of cell doublets was prevented.

- To remove doublets, we first excluded, in silico, the cell barcodes associated with a high number of transcripts (the thresholds are already indicated in the Methods section). Secondly, we used the tool Scrublet from the scran package (1.14.6) (Wolock et al., Cell Systems, 2019; PMID: 30954476) to calculate a numeric score to identify doublets. Third, we rely on marker gene expression to assess if a hybrid transcriptome could mimic the cell type in focus. For example, in the case of our Dkk2-positive stromal subpopulation, there might be a

subpopulation of cells with high expression of Dkk2 that could mimic the Dkk2-positive stromal phenotype when forming a doublet with a stromal cell. However, no such population exists in the datasets, where we have substantial Dkk2 expression in the stroma (Datasets IDs: G10, G12, H1).

- We have now included the information in the Methods section of the manuscript on page 26: "The expression matrices were filtered and potential doublets were removed by excluding barcodes with fewer than 250 genes, more than 4,000 genes and more than 15.000 Unique Molecule Identifiers (UMIs). Additionally, barcodes with more than 10 percent mitochondrial genes detected were excluded. Genes that were not detected for any barcode were also removed. Potential doublets were also scrutinized using Scrublet from the scran package (1.14.6) (Wolock et al 2019 Cell Systems; PMID: 30954476) and we used marker gene expression to assess if the cell type in focus could be mimicked by a hybrid transcriptome, but these analysis did not lead to further removal of cell-associated barcodes."

Decision Letter, second revision:

Date: 2nd October 20 13:17:16
Last Sent: 2nd October 20 13:17:16
Triggered By: Christine Weber
From: christine.weber@nature.com
To: tfm@mpiib-berlin.mpg.de
CC: ncb@springernature.com
Subject: Decision on Nature Cell Biology submission NCB-M41689B
Message: *Please delete the link to your author homepage, if you wish to forward this email to your co-authors.

Dear Thomas,

Thank you for submitting your revised manuscript, "Opposing Wnt signals regulate cervical squamocolumnar homeostasis and emergence of metaplasia" (NCB-M41689B). It has been evaluated again by our original reviewer 3 and the comments are below. I am pleased to say that in light of these comments, we shall be happy, in principle, to publish it in Nature Cell Biology, pending minor revisions to comply with our editorial and formatting guidelines.

Below you will find detailed guidelines for preparing your final submission. To avoid delays in the formal acceptance and publication of your manuscript, please follow these instructions closely and do not hesitate to contact me if you need clarification on any of these points.

We ask that you submit your revised manuscript files and all requested forms

within four weeks, using the link below. Please let us know if you foresee any issues with submitting within this timeframe.

[REDACTED]

Nature Cell Biology offers a transparent peer review option for new original research manuscripts submitted from 1st December 2019. We encourage increased transparency in peer review by publishing the reviewer comments, author rebuttal letters and editorial decision letters if the authors agree. Such peer review material is made available as a supplementary peer review file. **Please state in the cover letter 'I wish to participate in transparent peer review' if you want to opt in, or 'I do not wish to participate in transparent peer review' if you don't.** Failure to state your preference will result in delays in accepting your manuscript for publication.

Please note: we allow redactions to authors' rebuttal and reviewer comments in the interest of confidentiality. If you are concerned about the release of confidential data, please let us know specifically what information you would like to have removed. Please note that we cannot incorporate redactions for any other reasons. Reviewer names will be published in the peer review files if the reviewer signed the comments to authors, or if reviewers explicitly agree to release their name. For more information, please refer to our FAQ page.

In recognition of the time and expertise our reviewers provide to Nature Cell Biology's editorial process, we would like to formally acknowledge their contribution to the external peer review of your manuscript entitled "Opposing Wnt signals regulate cervical squamocolumnar homeostasis and emergence of metaplasia". For those reviewers who give their assent, we will be publishing their names alongside the published article.

When preparing your final files, please keep in mind that Nature Cell Biology is read by cell biologists from diverse backgrounds, many of whom are not native English speakers. Authors should aim to communicate their findings clearly, explaining technical jargon that might be unfamiliar to non-specialists, and avoiding non-standard abbreviations. Titles and abstracts should concisely communicate the main findings of the study, and the background, rationale, results and conclusions should be clearly explained in the manuscript in a manner accessible to a broad cell biology audience. Nature Cell Biology uses British spelling.

In particular, please ensure that you address the following editorial points:

TEXT FORMATTING:

(1) NCB Articles can have up to 3500 words in the main text, including a <150 word abstract, and Introduction, Results (divided in subsections) and Discussion sections. Please reduce the length of the main text and abstract (currently 5543 and 161 words, respectively) to conform to these limits, and provide the main text together with the main figure legends as a single Word file. Should you find it difficult to shorten the main text any further, we can permit an extension of the word limit to 4500 words.

(2) All key protein and gene names should be mentioned in the title and/or abstract to ensure optimal retrieval of the paper in database searches.

(3) Please refrain from using words such as new/novel/first, when referring to the scientific findings.

FIGURES AND TABLES:

(4) All key data should be presented in the main figures, with Extended Data only presenting supportive information. There is a limit of 6-8 display items for NCB Articles and all key data should be presented in these figures. Please restructure the current figures into 8 main and up to 10 extended data figures, using main figures to present the most important data. Please ensure that all figures adhere to a maximum page size of roughly 180mm wide x 200mm high to fit standard A4 page format and use a font size of no smaller than 7pt Arial or Helvetica throughout the figures. No peer-reviewed data should be removed.

(5) To ensure that the figure legends can fit on a single, print page together with the figure, the main legends should not exceed 350 words, and extended data legends should not exceed 300 words. Figure legends should be concise, and should begin with a brief title. The legends should describe what is presented in the figure and detail all relevant statistical information. It is best to avoid inappropriate methodological detail and any interpretation or discussion of figures in the legends.

(6) Scale bars need to be shown for all images and should be defined in the legends.

(7) Each figure must be submitted individually, with all the panels for each in a single file. Please do not embed figures in the manuscript file. Our preferred image formats listed below:
--Vector: ai, eps, pdf
--Raster (Bitmap): psd, tiff

(8) Figures must be submitted in a layered format with labels, text, lines/shapes and images individually selectable and on separate layers with all file-editing capabilities intact.

(9) The minimum resolution for colour figures, including photographic images, is 300 dpi. For grayscale, the minimum is 600 dpi, including blots and black & white photographs. Line art, such as graphs and illustrations, must be a minimum of 1200 dpi.

SUPPLEMENTARY INFORMATION:

All Supplementary Information must be submitted in accordance with the instructions in the attached Inventory of Supporting Information, and should fit into one of three categories:

natureresearch

1. EXTENDED DATA: Extended Data are an integral part of the paper and only data that directly contribute to the main message should be presented. These figures will be integrated into the full-text HTML version of your paper and will be appended to the online PDF. There is a limit of 10 Extended Data figures, and each must be referred to in the main text. Each Extended Data figure should be of the same quality as the main figures, and should be supplied at a size that will allow both the figure and legend to be presented on a single legal-sized page. Each figure should be submitted as an individual .jpg, .tif or .eps file with a maximum size of 10 MB each. All Extended Data figure legends must be provided in the attached Inventory of Accessory Information, not in the figure files themselves.

2. SUPPLEMENTARY INFORMATION: Supplementary Information is material that is essential background to the study but which is not practical to include in the printed version of the paper (for example, video files, large data sets and calculations). Each item must be referred to in the main manuscript and detailed in the attached Inventory of Accessory Information. Tables containing large data sets should be in Excel format, with the table number and title included within the body of the table. All textual information and any additional Supplementary Figures (which should be presented with the legends directly below each figure) should be provided as a single, combined PDF. Please note that we cannot accept resupplies of Supplementary Information after the paper has been formally accepted unless there has been a critical scientific error.

All Extended Data must be called out in your manuscript and cited as Extended Data 1, Extended Data 2, etc. Additional Supplementary Figures (if permitted) and other items are not required to be called out in your manuscript text, but should be numerically numbered, starting at one, as Supplementary Figure 1, not SI1, etc.

3. SOURCE DATA: We encourage you to provide source data for your figures whenever possible. Full-length, unprocessed gels and blots must be provided as source data for any relevant figures, and should be provided as individual PDF files for each figure containing all supporting blots and/or gels with the linked figure noted directly in the file. Statistics source data should be provided in Excel format, one file for each relevant figure, with the linked figure noted directly in the file. For imaging source data, we encourage deposition to a relevant repository, such as figshare (https://figshare.com/) or the Image Data Resource (https://idr.openmicroscopy.org).

REPORTING GUIDELINES:

We want to ensure that the methods and statistics reporting in our papers are of the highest quality. To that end, please submit an updated version of the Reporting Summary that collects information on experimental design and reagents.

The Reporting Summary will be published with the manuscript as part of the Supplementary Material, so please ensure that it is completed as closely as possible.

The Reporting Summary can be found here:
https://www.nature.com/documents/nr-reporting-summary.pdf

Please also submit an updated Editorial Policy Checklist, which will not be published. As such, please ensure that all pertinent information is also included in the manuscript itself.

The Policy Checklist can be found here:
https://www.nature.com/authors/policies/Policy.pdf

Please note that these forms are dynamic 'smart pdfs' and must be downloaded and completed in Adobe Reader. If you would like to reference the guidance text as you complete the template, please access these flattened versions at http://www.nature.com/authors/policies/availability.html.

STATISTICS REPORTING AND REPRODUCIBILITY:

(10) Wherever statistics have been derived (e.g. error bars, box plots, statistical significance) the legend needs to provide and define the n number (i.e. the sample size used to derive statistics) as a precise value (not a range), using the wording "n=X biologically independent samples/animals/independent experiments" etc. as applicable.

(11) Error bars need to be defined in the legends (e.g. SD, SEM) together with a measure of centre (e.g. mean, median).

(12) All box plots need to be defined in the legends in terms of minima, maxima, centre, and percentiles, and should be accompanied by their precise n number defined as noted above.

(13) Wherever statistical significance has been derived, precise p values need to be provided and the type of statistical test used needs to be defined in the legend.

(14) Statistics such as error bars cannot be derived from n<3, and must be removed from all such cases. We strongly discourage deriving statistics from technical replicates, and they should be removed from all such cases, unless there is a clear scientific justification for why providing this information is important. Conflating technical and biological variability, e.g. by pooling technically replicate samples across independent experiments is strongly discouraged.

(15) For sample sizes of n<10 please plot the individual data points rather than providing bar graphs.

(16) Please state in the legends how many times each experiment was repeated independently with similar results. This is needed for all experiments, but is particularly important wherever representative experiments are shown. If space in the legends is limiting, this information can be included in the "Statistics and Reproducibility" section.

(17) We require the presentation of source data for graphical and statistical analyses as separate Source Data files, and request that source data for all independent repeats are provided when representative experiments of multiple

independent repeats, or averages of two independent experiments are presented.

(18) The Methods should include a separate section titled “Statistics and Reproducibility” with general information on how the statistical analyses of the data were conducted, and general information on the reproducibility of experiments.

METHODS:

(19) There should be a single Methods section provided as a separate Word file, and providing all methodological details necessary for others to replicate the experiments. We generally recommend that the Methods do not largely exceed ~3000 words.

Please take special note of the following points:

(20) When citing previous methods, accurate references should be provided and any alterations should be noted. References only cited in the Methods section should be provided at the end of the Methods. Those cited in the main text should be included in the references in the main manuscript file.

(21) Complete information must be provided about: antibody dilutions, company names, catalogue numbers and clone numbers for monoclonal antibodies; sequences of RNAi and cDNA probes/primers or company names and catalogue numbers if reagents are commercial; cell line names, sources and information on cell line identity and authentication. If this information is lengthy, it may be provided as separate Supplementary Table(s) in Excel format and should be referenced in the Reporting Summary and the Methods.

(22) Animal studies and experiments involving human subjects must be reported in detail, identifying the committees approving the protocols.

-Information on animal species, strains, ages, gender (even if not selected for gender), needs to be included for all relevant experiments. If no animals were used please state that no animals were used. An ethics statement affirming that the study is compliant with all relevant ethical regulations regarding animal research must also be included.

- For studies involving human subjects/samples, a statement must be included confirming that informed consent was obtained, and that the study is compliant with all relevant ethical regulations regarding research involving human participants. Information on human research participants (age, gender, genotypic information, diagnosis and treatment categories), needs to be included. If no human participants were involved please state in the Reporting Summary that the study did not involve human research participants.

(23) Please provide information on any software used in data analysis in the Reporting Summary. Please also include a section in the methods titled “code availability” providing information on how the computational code used in the study can be obtained.

This journal strongly supports public availability of data. Please place the data used in your paper into a public data repository, or alternatively, present the data as Supplementary Information. If data can only be shared on request, please explain why in your Data Availability Statement, and also in the correspondence with your editor. Please note that for some data types, deposition in a public repository is mandatory - more information on our data deposition policies and available repositories appears below.

(24) Deposition in approved public repositories is mandatory for all sequencing and microarray data generated for the present study (designated as "primary accessions"). Providing the database name and accession numbers in the "Data Availability" section, (see next point), and ensuring that primary datasets are publicly available, is required for formal acceptance of the manuscript publication. Database names and accession numbers (or other unique identifiers such as URLs, DOIs) are also required for secondary datasets (i.e. published datasets reanalysed in the present study, designated as "referenced accessions"). See http://www.nature.com/authors/policies/availability.html for more information.

(25) All Nature Cell Biology manuscripts should include a Data availability statement at the end of the Methods section. For Springer Nature policies on data availability see the links below:
http://www.nature.com/authors/policies/availability.html
For more information on this particular policy see:
http://www.nature.com/authors/policies/data/data-availability-statements-data-citations.pdf.

The Data availability statement should include:
--Accession codes for primary datasets (generated during the study under consideration and designated as "primary accessions") and secondary datasets (published datasets reanalysed during the study under consideration, designated as "referenced accessions"). For primary accessions data should be made public to coincide with publication of the manuscript.
A list of data types for which submission to community-endorsed public repositories is mandated (including sequence, structure, microarray, deep sequencing data) can be found here: http://www.nature.com/authors/policies/availability.html#data.

--At a minimum, please include a statement confirming that all relevant data are available from the authors, mention any restrictions on availability and/or are included with the manuscript. If included in the manuscript, list where this data can be found

-- If a dataset has a DOI as its unique identifier, we strongly encourage including this in the Reference list and citing the dataset in the Methods.

An example "Data Availability" statement follows, but please modify as appropriate to ensure that it applies to the data included in this study. Including this information is mandatory for publication of the manuscript:

"Data availability

Microarray and scRNA–seq data that support the findings of this study have been

deposited in the Gene Expression Omnibus (GEO) under accession codes GSE87076 and GSE128987.

Previously published microarray data that were re-analysed here are available under accession code GSEZZZZZ [reference number].

The human cervical cancer data were derived from the TCGA Research Network: http://cancergenome.nih.gov/. The data-set derived from this resource that supports the findings of this study is available in [repository information and link, or details of supplementary information files].

All other data supporting the findings of this study are available from the corresponding author on reasonable request."

LEGAL AND POLICY POINTS:

Before we can move ahead with acceptance and publication of your paper we must receive formal written permission to use, edit, publish and distribute your work. Please note that you will retain copyright for your paper, but that you are granting Nature Research certain rights and permissions for the full term of copyright and any extension thereto, as outlined in the License to Publish form.

Please complete, sign, and return the form linked below. Our legal department demands that we no longer accept "e-signatures" on any legal document, so please return the form with a handwritten signature, or an embedded image of a handwritten signature. All legal forms must be signed by the corresponding author.

The form can be found here:
http://www.nature.com/documents/snl-ltp.docx

If the corresponding author is either a Crown government employee (including Great Britain and Northern Ireland, Canada and Australia), or a US Government employee a Government LTP, linked below:

UK Government: http://www.nature.com/documents/snl-ltp-crown.docx
US Government: http://www.nature.com/documents/snl-ltp-govus.docx

All contributing authors must be listed on the License to Publish. If you cannot fit the names in the provided field, please instead write "see attached page" and include a second page with the full author list.

It is your responsibility to obtain the right to use any items (figures, tables, images, videos or text boxes) that are reproduced (or adapted) from material for which you do not hold copyright and to give proper attribution to the creators of that work. This includes work that has previously been published elsewhere.

If you do not hold the copyright for any such item (in whole or part) that is included in your paper, you must also complete and return the Third Party Rights Table linked below:

http://www.nature.com/documents/thirdpartyrights-origres.doc

The Nature Research Journals are committed to providing authors with the highest quality figures to accompany their printed work, and we use the latest techniques to achieve this reproduction. As such, we charge authors a fee of €350/ £307/ $395 per colour figure.
Please sign and return the form linked below to assist us in billing for these costs:
https://www.nature.com/documents/nr-rj-colour-figure-form.pdf

FINANCIAL AND NON-FINANCIAL COMPETING INTERESTS -- Nature Research defines Competing Interest (CI) as financial and non-financial interests (including but not limited to funding, employment, stocks, shares, patents, personal or professional relationships with individuals or institutions, unpaid membership advocacy) that could or could be perceived to directly undermine the objectivity, integrity and value of a publication, or could be seen as having an influence on the judgments and actions of authors with regard to objective data presentation, analysis and interpretation.
Please thoroughly review our policy on Competing Interests (linked below) and include a detailed statement in your final manuscript file, and in our manuscript tracking system. Please ensure the statements are identical in both. Be specific about how each point stated relates to the research, list applicable author initials, and/or patent numbers.

Conflict of Interest Policy:
https://www.nature.com/nature-research/editorial-policies/competing-interests

If there are no competing interests, a negative statement must be included.

Please don't hesitate to let me know if I can help in any way.

With best wishes,

Christine.

Christine Weber, PhD
Senior Editor
Nature Cell Biology
E-mail: christine.weber@nature.com
Phone: +44 (0)207 843 4924

Reviewers' Comments:

Reviewer #3:
Remarks to the Author:
Authors have satisfactory addressed all remaining concerns.

naturerésearch

Final Decision Letter:

Date: 26th November 20 21:55:39

Last Sent: 26th November 20 21:55:39

Triggered By: Zhe Wang

From: zhe.wang@nature.com

To: tfm@mpiib-berlin.mpg.de

CC: ncb@springernature.com

BCC: rjsproduction@springernature.com;rjsart@springernature.com

Subject: Decision on Nature Cell Biology submission NCB-M41689C

Message: Dear Dr Meyer,

I am pleased to inform you that your manuscript, "Opposing Wnt signals regulate cervical squamocolumnar homeostasis and emergence of metaplasia", has now been accepted for publication in Nature Cell Biology.

Thank you for sending us the final manuscript files to be processed for print and online production, and for returning the manuscript checklists and other forms. Your manuscript will now be passed to our production team who will be in contact with you if there are any questions with the production quality of supplied figures and text.

Before the manuscript is sent to our printers, we will make changes in the text that may be necessary either to make it conform with house style or to make it intelligible to our wide readership. We look particularly carefully at the titles of all papers to ensure that indexing will be accurate and that they are not unreasonably long. We will ask your approval before the copy is finalized, and you will soon receive the edited proofs. Please check the text and figures carefully. Once your manuscript is typeset you will receive a link to your electronic proof via email within 20 working days, with a request to make any corrections within 48 hours. If you have queries at any point during the production process then please contact the production team at rjsproduction@springernature.com. Once your paper has been scheduled for online publication, the Nature press office will be in touch to confirm the details. An online order form for reprints of your paper is available at https://www.nature.com/reprints/author-reprints.html. All co-authors, authors' institutions and authors' funding agencies can order reprints using the form appropriate to their geographical region.

Publication is conditional on the manuscript not being published elsewhere and on there being no announcement of this work to any media outlet until the online publication date in Nature Cell Biology.

The Author's Accepted Manuscript (the accepted version of the manuscript as submitted by the author) may only be posted 6 months after the paper is published, consistent with our self-archiving embargo. Please note that the Author’s Accepted Manuscript may not be released under a Creative Commons license. For Nature Research Terms of Reuse of archived manuscripts please see: http://www.nature.com/authors/policies/license.html#terms
If you have posted a preprint on any preprint server, please ensure that the preprint details are updated with a publication reference, including the DOI and a URL to the published version of the article on the journal website.

To assist our authors in disseminating their research to the broader community, our SharedIt initiative provides you with a unique shareable link that will allow anyone (with or without a subscription) to read the published article. Recipients of the link with a subscription will also be able to download and print the PDF.

As soon as your article is published, you will receive an automated email with your shareable link.

If you have not already done so, we strongly recommend that you upload the step-by-step protocols used in this manuscript to the Protocol Exchange (www.nature.com/protocolexchange), an open online resource established by Nature Protocols that allows researchers to share their detailed experimental know-how. All uploaded protocols are made freely available, assigned DOIs for ease of citation and are fully searchable through nature.com. Protocols and the Nature and Nature research journal papers in which they are used can be linked to one another, and this link is clearly and prominently visible in the online versions of both papers. Authors who performed the specific experiments can act as primary authors for the Protocol as they will be best placed to share the methodology details, but the Corresponding Author of the present research paper should be included as one of the authors. By uploading your Protocols to Protocol Exchange, you are enabling researchers to more readily reproduce or adapt the methodology you use, as well as increasing the visibility of your protocols and papers. You can also establish a dedicated page to collect your lab Protocols. Further information can be found at www.nature.com/protocolexchange/about

You can use a single sign-on for all your accounts, view the status of all your manuscript submissions and reviews, access usage statistics for your published articles and download a record of your refereeing activity for the Nature journals.

Please feel free to contact us if you have any questions.

With kind regards,

nature research

Zhe Wang, PhD
Senior Editor
Nature Cell Biology

Tel: +44 (0) 207 843 4924
email: zhe.wang@nature.com
